# miRNA-206-3p alleviates LPS-induced acute lung injury via inhibiting inflammation and pyroptosis through modulating TLR4/NF-κB/NLRP3 pathway

**DOI:** 10.1038/s41598-024-62733-5

**Published:** 2024-05-24

**Authors:** Mengchi Chen, Jingfeng Zhang, Hongyuan Huang, Zichen Wang, Yong Gao, Jianghua Liu

**Affiliations:** 1 The Second Affiliated Hospital of Guangxi Medical University, Nanning, 530000 Guangxi China; 2 Health Management Center of The Sixth Affiliated Hospital, School of Medicine, South China University of Technology, Foshan, 528200 Guangdong China; 3 The First Affiliated Hospital of Guangxi Medical University, Nanning, 530000 Guangxi China; 4 School of Nursing, Guangxi Medical University, Nanning, 530000 Guangxi China

**Keywords:** ALI, miRNA-206-3p, Pyroptosis, Inflammation pathway, NLRP3, Cell biology, Molecular biology, Diseases, Medical research, Pathogenesis

## Abstract

Acute lung injury (ALI) is life-threatening. MicroRNAs (miRNAs) are often abnormally expressed in inflammatory diseases and are closely associated with ALI. This study investigates whether miRNA-206-3p attenuates pyroptosis in ALI and elucidates the underlying molecular mechanisms. ALI mouse and cell models were established through lipopolysaccharide (LPS) treatment for 24 h. Subsequently, the models were evaluated based on ultrasonography, the lung tissue wet/dry (W/D) ratio, pathological section assessment, electron microscopy, and western blotting. Pyroptosis in RAW264.7 cells was then assessed via electron microscopy, immunofluorescence, and western blotting. Additionally, the regulatory relationship between miRNA-206-3p and the Toll-like receptor (TLR)4/nuclear factor (NF)-κB/Nod-like receptor protein-3 (NLRP3) pathway was verified. Finally, luciferase reporter gene and RNA pull-down assays were used to verify the targeting relationship between miRNA-206-3p and TLR4. miRNA206-3p levels are significantly decreased in the LPS-induced ALI model. Overexpression of miRNA-206-3p improves ALI, manifested as improved lung ultrasound, improved pathological changes of lung tissue, reduced W/D ratio of lung tissue, release of inflammatory factors in lung tissue, and reduced pyroptosis. Furthermore, overexpression of miRNA-206-3p contributed to reversing the ALI-promoting effect of LPS by hindering TLR4, myeloid differentiation primary response 88 (MyD88), NF-κB, and NLRP3 expression. In fact, miRNA-206-3p binds directly to TLR4. In conclusion, miRNA-206-3p alleviates LPS-induced ALI by inhibiting inflammation and pyroptosis via TLR4/NF-κB/NLRP3 pathway modulation.

## Introduction

Since the COVID-19 pandemic, its associated acute lung injury (ALI; all abbreviations are defined in Table 1) has attracted renewed attention. ALI is a common and critical health issue that is challenging to manage. It is characterized by the cellular damage, such as alveolar epithelial cells and capillary endothelial cells, and results from various direct or indirect injury-associated factors. These factors can induce fluid accumulation in the pulmonary interstitium and alveoli, leading to diffuse pulmonary edema and, subsequently, acute respiratory insufficiency due to low oxygen levels^1^. Once ALI becomes critical, it is commonly known as acute respiratory distress syndrome (ARDS)^2^. Although ventilator support therapy, fluid management, and nutritional support are key treatment strategies for ALI, their therapeutic effectiveness remains limited^3,4^. The annual in-hospital mortality rate of newly diagnosed adult ALI/ARDS patients in the United States is as high as 38.5%, while that of older adults is nearly 60%^5^. Given the high mortality rate and severe financial burden associated with ALI, new therapeutic strategies are urgently required.

**Table 1:** Abbreviations and full names.

Abbreviation	Definition
ALI	Acute lung injury
ASC	Apoptosis-associated speck-like protein containing a caspase recruitment domain
DAPI	4′6- Diamidino-2-phenylindole
DMEM	Dulbecco′s Modified Eagle Medium
ELISA	Enzyme-linked immunosorbent assay
FBS	Fetal bovine serum
GAPDH	Glyceraldehyde 3-phosphate dehydrogenase
GFP	Green fluorescent protein
GSDMD	Gasdermin D
H&E	Hematoxylin and eosin
LPS	Lipopolysaccharide
NS	Normal saline
PBS	Phosphate-buffered saline
PCR	Polymerase chain reaction
PVDF	Polyvinylidene difluoride
RIPA	Radioimmunoprecipitation assay
SDS-PAGE	Sodium dodecyl-sulfate polyacrylamide gel electrophoresis
SD	Standard deviation
SPF	Specific pathogen free
TBST	Tris-buffered saline and Tween

A developing body of evidence strongly suggests that inflammation and pyroptosis have a significant effect on ALI progression^6^. The term *pyroptosis* was coined in 2001 to describe the pro-inflammatory programmed cell death distinct from apoptosis observed in Salmonella-induced macrophages^7^. However, its definition was revised in 2015 to include gasdermin-mediated programmed cell death—distinct from apoptosis^8^. Unlike apoptosis, which is considered a safe mode of cell death, pyroptosis induces inflammation and is triggered by various factors, including bacteria, viruses, toxins, and chemotherapeutics^9^. Importantly, reducing both inflammation and pyroptosis improves outcomes in ALI^10^. Lipopolysaccharide (LPS) —a crucial constituent in the outer membrane of Gram-negative bacteria—is commonly used to induce ALI in experimental models. Accumulation of white blood cells in lung tissue, pulmonary edema, and severe lung inflammation are the characteristics of this in vivo ALI model^11^. Yang et al. conduct a study which demonstrated that corticosteroids can alleviate inflammation and lung injury caused by LPS by modulating Nod-like receptor protein-3 (NLRP3) activation^12^. Meanwhile, Zhang et al. reported that metformin can alleviate LPS-induced ALI by increasing sirtuin 1 expression, which inhibits nuclear factor κB (NF)-κB/NLRP3-mediated pyroptosis^13^. Therefore, focusing on the activation of inflammatory processes and potential targets of pyroptosis may provide novel ideas for overcoming ALI.

Gene regulation is a crucial process encompassing transcription, DNA methylation, and chromatin modification^14^. MicroRNAs (miRNAs) are short non-coding RNA molecules containing 18–23 nucleotides; a single miRNA can interact with many target genes to direct cellular pathways^15^. Numerous studies have identified the indispensable role of miRNAs in various stages of inflammation, ranging from initiation to expansion and resolution. This is achieved through a combination of positive and negative feedback mechanisms in inflammatory lung diseases, including ALI/ARDS^16^. For example, miRNAs, such as miRNA-9, miRNA-127, and miRNA-223, which regulate macrophage polarization, hold the potential for therapeutic intervention in inflammation-related illnesses^17^. However, miRNAs can elicit anti-inflammatory or pro-inflammatory effects, and their targets and functions are complex and largely unknown. Therefore, understanding the mechanisms of action of miRNAs could present a novel therapeutic avenue for managing the pathogenesis of inflammatory diseases.

The current study used a public bioinformatics database to predict abnormal miRNA expression associated with ALI and identify specific miRNAs. miRNA-206-3p, the main target of this study, was found to be downregulated in LPS-induced ALI mice. Overexpression of miRNA-206-3p plays a significant protective role in ALI by suppressing the inflammatory processes and pyroptosis. Furthermore, we found that miRNA206-3p directly targets and binds to TLR4; hence, the TLR4/NF-κB/NLRP3 pathway contributes to the regulatory role of miRNA-206-3p in ALI. This study holds research value and scientific significance for identifying novel molecular therapeutic targets for ALI.

## Material and methods

### Animals

BABL/c male mice (18–22 g) were procured from Beijing SPF Biotechnology Co., Ltd. The study was performed in accordance with the ARRIVE guidelines. All the experimental protocols were approved by the Medical Ethics Committee of the Second Affiliated Hospital of Guangxi Medical University (ethical review number: 202101029). Animal experimental procedures strictly complied with the U.K Animals (Scientific Procedures) Act, 1986 ethical requirements for biomedical research. All mice were raised in a specific pathogen-free (SPF) animal house at 22–25 °C, with abundant food, fresh water, and a 12 h light/dark cycle. The BABL/c male mice were randomly divided into four groups: control, ALI or LPS, LPS + agomir-206-3p, and LPS + agomir-NC.

### Establishment of animal model

The procedure outlined by Chao Cao was adopted to establish the LPS-induced mouse ALI model^18^. The first control group, received 10 mg/kg normal saline (NS) via intratracheal instillation. The second group, i.e., the ALI or LPS group, received an intratracheal instillation of 10 mg/kg LPS (Cat No L2880, Sigma, US). The LPS + agomir-206-3p group was administered a combination of 10 mg/kg LPS and 1 nmol/L agomir-206-3p. The LPS + agomir-NC group, was administered a combination of 10 mg/kg LPS and 1 nmol/L agomir-NC. Agomir-206-3p and agomir-NC were synthesized by RiboBiotech (Guangzhou, China). Agomir-206-3p (1 nmol/each) or agomir-NC (1 nmol/each) was divided into three days of tail intravenous injection until ALI models were induced as described above.

### Pulmonary ultrasonography

After reaching the intervention time, mice in each group were transferred to the ultrasound department for a lung ultrasound scan to obtain lung ultrasound images.

### Wet-to-dry (W/D) ratio of the lungs

When the intervention time endpoint was reached, the mice were humanely sacrificed with an overdose of sodium pentobarbital anesthesia. Their chests were opened, and the lung were extracted. Subsequently, the superior right lung was separated and rinsed with saline, surface moisture was absorbed using absorbent paper, and wet weight was measured. After incubation at 80 °C for 24 h, the dry weight of the sample was determined, and the W/D weight ratio was calculated.

### Histopathological analysis

At the end of the intervention, the right lung was resected in each experimental group, fixed with 10% neutral formalin solution for 24 h, dehydrated with gradient alcohol, cleared, paraffin-embedded, sliced at 4 mm thick, and then stained with hematoxylin and eosin. The images were acquired under a pathological microscope.

### Quantitative real-time PCR (q-RT PCR)

Total RNA was isolated from the lung tissues or cells using Trizol reagent and chloroform. Reverse transcription and qRT-PCR assays for miRNA were performed using corresponding Sangon kits (Sangon Biomedical Technology Co., Ltd.; Shanghai, China). U6 was designated as the internal reference for normalization. Reverse transcription and qRT-PCR assays for other common mRNAs were performed using corresponding TAKARA kits (Takara Biomedical Technology Co., Ltd.; Beijing, China). *Gapdh* was designated as the internal reference for normalization. The primer sequences are listed in Table 2.

**Table 2:** Primer sequences.

Gene	Species	Primer sequence(5′–3′)	Product length/bp
miRNA-206-3p-RT	*Mus musculus*	CCTGTTGTCTCCAGCCACAAAAGAGCA	56
		CAATATTCCAGGAGACAACAGGCCACACA	
miRNA-206-3p	*Mus musculus*	F:CGGGCTGGAATGTAAGGAAG	20
		R:CAGCCACAAAAGAGCACAAT	20
U6-RT	*Mus musculus*	GTCGTATCCAGTGCAGGGTCCGAGG TATTCGCACTGGATACGACAAAAAT	50
U6	*Mus musculus*	F:GAAGATTTAGCATGGCCCCTGC	22
		R:CAGTGCAGGGTCCGAGGT	18
TLR4	*Mus musculus*	F:ATGGCATGGCTTACACCACC	20
		R:GAGGCCAATTTTGTCTCCACA	21
MyD88	*Mus musculus*	F:AGCAGACAGTGGCAGTATGGGTTAG	23
		R:GGGCAGTAGCAGATAAAGGCATCG	24
NF-κB	*Mus musculus*	F:TGAGGCTGAGCGGAGGTGATG	21
		R:AGGAGACAGTGGCAGTATGGGTTAG	25
NLRP3	*Mus musculus*	F:GCTGCGATCAACAGGCGAGAC	21
		R:CCATCCACTCTTCTTCAAGGCTGTC	25
Caspase-1	*Mus musculus*	F:ACAAGGCACGGGACCTATG	19
		R:TCCCAGTCAGTCCTGGAAATG	21
IL-18	*Mus musculus*	F:AGACCTGGAATCAGACAACTTT	22
		R:TCAGTCATATCCTCGAACACAG	22
IL-1β	*Mus musculus*	F:CTCGCAGCAGCACATCAACAAG	22
		R:CCACGGGAAAGACACAGGTAGC	22
GSDMD	*Mus musculus*	F: AGACAATAGACCCCTCCCC	19
		R: TCTGCTGCCGCTTACCTCC	19
GAPDH	*Mus musculus*	F: GGCACAGTCAAGGCTGAGAATG	22
		R: ATGGTGGTGAAGACGCCAGTA	21

### Enzyme-linked immunosorbent assay (ELISA)

The levels of cytokines in lung tissue homogenates and cell culture supernatants were detected with corresponding ELISA kits [including tumor necrosis factor (TNF)-α, interleukin (IL)-6, IL-1β, and IL-18] from Yuanju Biotechnology Co., Ltd (Shanghai, China).

### Cell culture

RAW264.7 macrophages were acquired from Wuhan Procell Life Science and Technology Co. Ltd. The cells were incubated in a stable environment at 37 °C with 5% CO_2_ and cultured in fresh Dulbecco′s modified eagle medium (DMEM) media containing 10% fetal bovine serum (FBS) and 1% penicillin-streptomycin (MeilunBio, Dalian, China). RAW264.7 cells were treated with 10 μg/mL LPS for 24 h to construct an in vitro model of inflammation and pyroptosis.

### Lentivirus transfection

The RAW264.7 cells were transfected with miRNA-206-3p using a lentiviral vector obtained from GeneChem Company (Shanghai, China). Briefly, the cells were cultured in lentivirus-containing medium for 16 h, after which the media was replaced and incubated for an additional 48 h. Subsequently, the cells were screened using puromycin. The signal emitted by green fluorescent protein (GFP) was visualized using a fluorescence microscope, and the effectiveness of gene transfection was confirmed using qRT-PCR analysis.

### Electron microscopic examination of cellular pyroptosis

RAW264.7 cells were fixed using an electron microscope fixative (G1102, Servicebio), rinsed with a buffer solution, treated with 1% osmic acid for fixation, gradually dehydrated with acetone, embedded with an embedding agent, and finally prepared for imaging. An electron microscope was used to evaluate the organelle structure.

### Western blotting

RIPA lysis buffer (RIPA buffer, R0010, Solarbio) and protease inhibitor mixtures were used to prepare lung tissues or cells protein samples. A bicinchoninic acid (BCA) protein assay kit (WB6501, New Cell & Molecular Biotech) was used to determine protein concentrations. Proteins in each group were resolved by electrophoresis on 10% or 12.5% sodium dodecyl-sulfate polyacrylamide gel electrophoresis (SDS-PAGE) gels, transferred onto polyvinylidene fluoride (PVDF) membranes (Millipore, Billerica, MA, USA; cut prior to hybridization with antibodies), blocked with a rapid blocking solution, and washed with TBST. Next, the PVDF membranes were incubated overnight at 4 °C with a primary antibody against TLR4 (Cat No. 66350-1-Ig; 1:1000), NF-κB (Cat No. T55034F; 1:5000), myeloid differentiation Factor 88 (MyD88) (Cat No. 23230–1-AP; 1:1000), NLRP3 (Cat No. MA5-23919; 1:2500), Apoptosis-associated speck-like protein containing a caspase recruitment domain (ASC) (Cat No.340097; 1:1000), Gasdermin-D (GSDMD) and N-terminal domain (GSDMD-N) (Cat No. ab219800; 1:2500), caspase-1 and cleaved caspase-1 (Cat No. 14-9832-82; 1:2500), Interleukin (IL)-18 (Cat No IPB0723; 1:2000), IL-1β (Cat No. P50520-1R1F; 1:1000), β-Actin (Cat No. GB15001-100; 1:2000) or GAPDH (Cat No. 60004-1-Ig; 1:10,000). The PVDF membranes were then incubated with an HRP-conjugated secondary antibody at room temperature for 1 h. To visualize and quantify the bands, a chemiluminescence detection system (Tanon 5200, Shanghai, China) and Image J software were used.

### Immunofluorescence

Referring to the immunofluorescence experimental procedure described by Li et.al^19^, RAW264.7 cells in different groups were fixed, permeated, blocked, and then incubated with NLRP3 (Cat No. MA5-23919; 1:200) and IL-18 (Cat No IPB0723; 1:200) primary antibodies at 4 °C overnight. The fluorescent secondary antibodies (GB23302, GB23303, Servicebio, 1:500) against the corresponding species were then incubated for 1 h and stained with 4′,6-diamidino-2-phenylindole (DAPI) for 10 min. After every step, the cells were thoroughly cleaned using phosphate-buffered saline (PBS) trice. Slides were observed under a fluorescence microscope.

### Luciferase reporter assay

Using a public database, we predicted that the TLR4 segment encompasses binding sites for miRNA-206-3p. Hence, the TLR4-WT and TLR4-MUT vectors were constructed by Guangzhou Ribobio Co., Ltd. Subsequently, 293T cells were co-transfected for 6 h with the reported vectors, miRNA-206-3p mimic and NC mimic, followed by replacement with a fresh medium for 48 h. The luciferase activity was measured using a Dual- Luciferase Reporter Assay Kit (Ribobio Co., Ltd. Guangzhou, China).

### RNA Pull-down

We designed and synthesized the NC Probe and miRNA-206-3p-WT Probe with the following sequences: NC Probe: Biotin, 5′-GACUUGAAGCCUAGCCCGAUCG-3′, miR-206-3p-WT Probe Biotin, 5′-CUUACAUUCCAUAGUGCUGAGA-3′. Normal cultured RAW264.7 cells (2 × 10^6^) were used to extract cytoplasmic proteins. Biotin-labeled RNA probes were then incubated with cytoplasmic protein extracts for pull-down assays. The composite was then treated with magnetic beads, eluted and denatured. Finally, the samples were silver dyed and western blotting was performed.

### Statistical analysis

Statistical analyses and chart generation were performed using GraphPad Prism 9 and SPSS 23.0 software. Data were presented as mean values ± standard deviation (SD) obtained from three distinct experiments. An independent sample *t*-test was conducted to compare two groups. *P* < 0.05 were considered statistically significant^20^.

## Results

### miRNA206-3p is downregulated in LPS-induced ALI in vitro and in vivo

**Figure 1:**
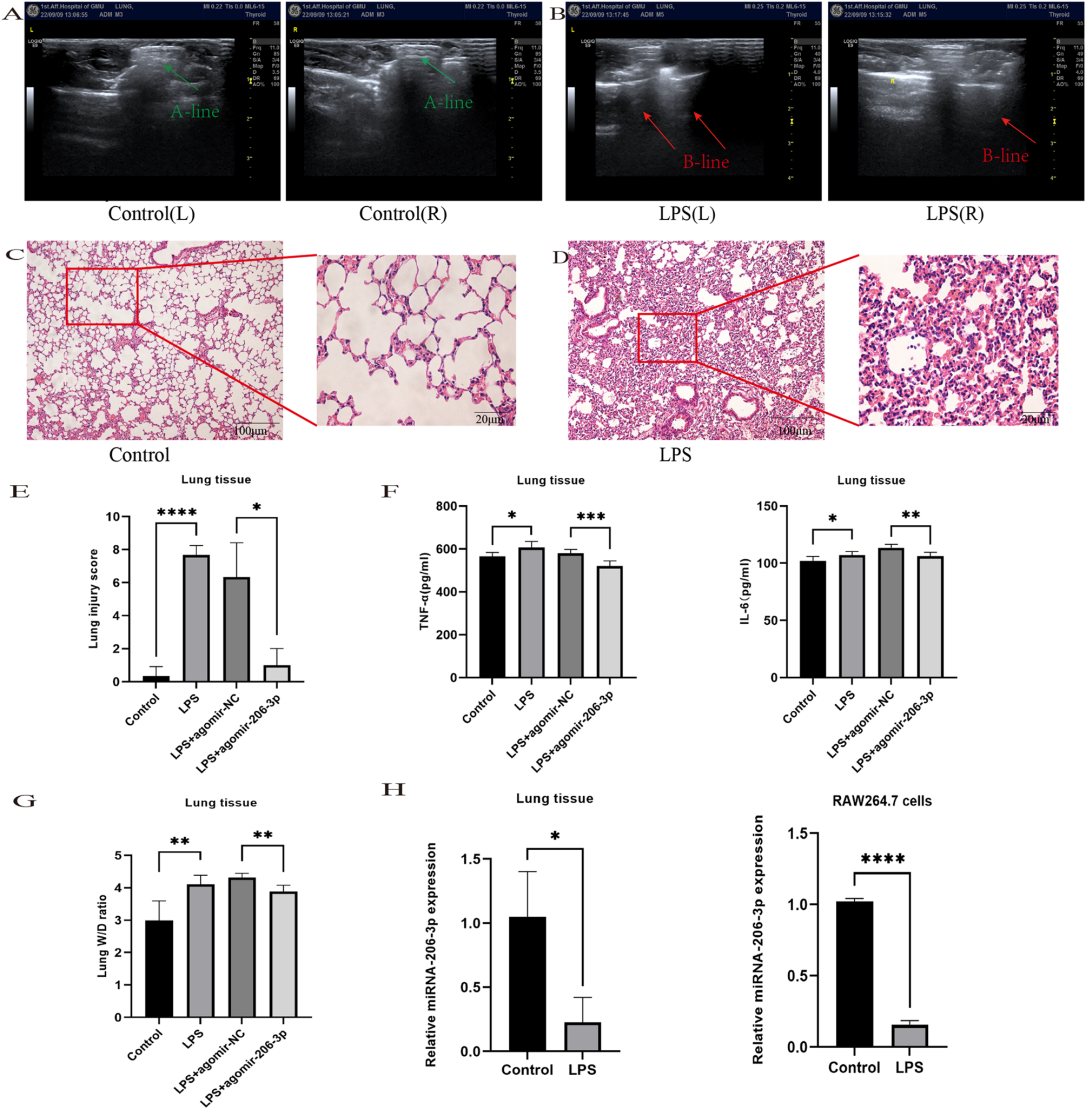
The miRNA-206-3p is downregulated in LPS-induced acute lung injury in mice (LPS: 10 mg/kg, 24 h) and RAW264.7 cells (LPS: 10 μg/mL 24 h). (A, B) Ultrasound images of mouse lungs. (C, D) Histological analysis of lung tissues from each experimental group. (E) Lung injury score (n = 6/group). (F) TNF-α and IL-6 levels in lung tissue were measured after LPS challenge (n = 6/group). (G) Lung wet/dry assay (n = 6/group). (H, I) The miRNA-20 6-3p expression was validated by qRT-PCR in lung tissues of mice (n = 6/group) and RAW264.7 cells (n = 3/group) challenged with LPS. Data represent the mean ± SD of three independent experiments. *P < 0.05, **P < 0.01, ***P < 0.001, ****P < 0.0001.

As described previously^18^, the animal ALI models were established through tracheal infusion of LPS. Pulmonary ultrasound is an important bedside tool for detecting lung conditions. The A-line is a normal pulmonary manifestation, while the B-line is a tail artifact indicating edema in the subpleural interstitial^21^. In the LPS group, edema was observed, as indicated by the B-line marking on lung ultrasound, compared to the control group (Fig. 1A,B). Subsequently, histopathological analysis revealed significant histological damage and apparent inflammatory features, including edema, necrosis, and neutrophil infiltration (Fig. 1C,D). Furthermore, the LPS group exhibited a higher lung injury score and lung W/D ratio than the control group (Fig. 1E,G). The abundance of inflammatory factors (TNF-α and IL-6) was also significantly elevated in the lung tissue of the LPS group (Fig. 1F), suggesting that LPS administration successfully established an ALI animal model.

miRNA-206-3p reportedly has a pivotal role in the progression of various human ailments, including cancers, osteoarthritis, and depression^22^. Meanwhile, the essential role of miRNA-206-3p in regulating the inflammatory response has also been described^23–25^. However, the role of miRNA-206-3p play in ALI remains unknown.

To verify the miRNA-206-3p expression trends in ALI and in vitro inflammatory models, qRT-PCR was performed across all groups. Compared to the normal group, the miRNA-206-3p expression level in the lung tissues of the ALI group decreased significantly (Fig. 1H). Because of the lack of alveolar lavage fluid in mice and the technical limitations of primary cell culture, we selected RAW264.7 cells for in vitro analyses. We found that miRNA-206-3p expression was also downregulated in LPS-treated RAW264.7 cells (Fig. 1H). Accordingly, we hypothesized that miRNA-206-3p plays a crucial role in f ALI progression.

### Upregulation of miRNA-206-3p attenuates LPS-induced ALI

**Figure 2:**
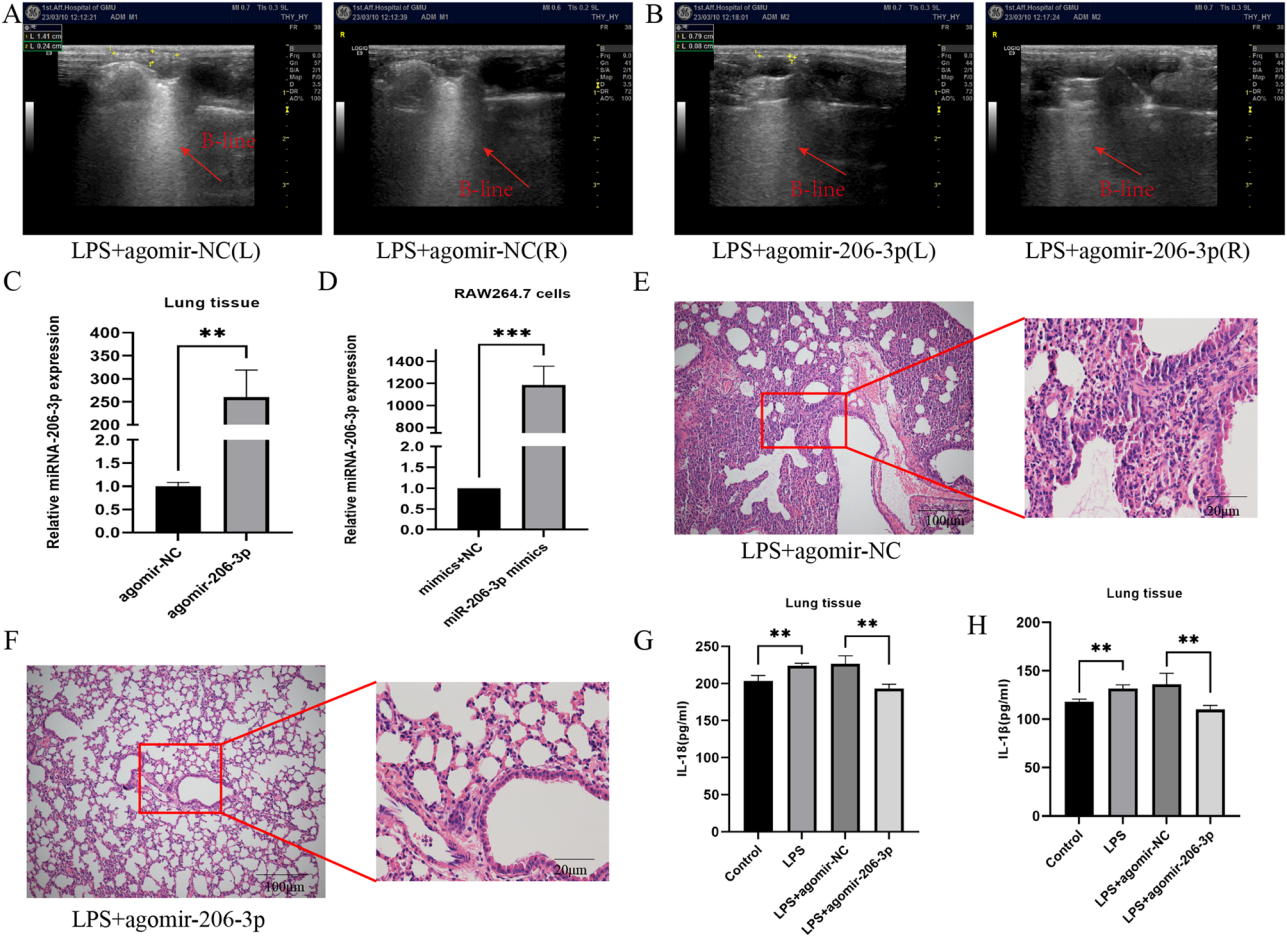
Increased miRNA-206-3p ameliorated LPS-induced ALI in mice (LPS: 10 mg/kg, 24 h) and RAW264.7 cells (LPS: 10 μg/mL, 24 h). (A, B) Ultrasound images of mouse lungs. (C, D) miRNA-206-3p expression detected by qRT-PCR after LPS + agomir-206-3p (n = 6/group) and LPS + miRNA-206-3p mimics (n = 3/group) treatment. (E, F) Histological evaluation of lung tissues from each experimental group. (G, H) IL-18 and IL-1β levels in lung tissue were measured after LPS or LPS + agomir-206-3p challenge (n = 6/group). Data represent the mean ± SD of three independent experiments. **P < 0.01, ***P < 0.001.

To investigate whether enhancing the expression of miRNA-206-3p can effectively ameliorate LPS-induced ALI, 1 nmol/mouse agomiR-206-3p was administered via tail vein injection to the mice before LPS treatment. At the endpoint of the experiment, the lungs were examined via ultrasonography. The condition of the lungs in the LPS + agomiR-206-3p group was notably better than that of mice in the LPS + agomiR-206-3p NC group (Fig. 2A,B).

Lentivirus transfection was used to construct a miRNA-206-3p mimic model of RAW264.7 cells. We constructed the model by evaluating the miRNA-206-3p expression using qRT-PCR, which showed that miRNA-206-3p levels increased in the lung tissues of mice in the LPS + agomiR-206-3p group of mice (Fig. 2C); the same results were observed in LPS + miRNA-206-3p mimics group of RAW264.7 cells (Fig. 2D).

Furthermore, compared to the LPS + agomir-NC group, pathological analysis indicated that the severity of lung lesions in the LPS + agomiRNA-206-3p group was relieved (Fig. 2E,F). Similarly, a comparison of the lung W/D ratios between the two groups confirmed improvement in the W/D ratio (Fig. 1G). Considered together, these results suggest that miRNA-206-3p attenuates LPS-induced ALI.

### Overexpression of miRNA-206-3p inhibits the inflammatory response and pyroptosis

Given the positive protective effects observed in prior animal trials concerning miRNA-206-3p, we investigated how augmenting miRNA-206-3p expression affects LPS-induced inflammatory reactions. Initially, pro-inflammatory cytokines in the homogenized lung tissue, including TNF-α and IL-6, were significantly elevated in the LPS group compared to the control group. However, the release of these cytokines was markedly suppressed with the overexpression of miRNA-206-3p (Fig. 1F). Interestingly, secretion of pyroptosis-related cytokines IL-18 and IL-1β was significantly elevated when detected in ALI lung homogenate. Meanwhile, their levels decreased with the increase in miRNA-206-3p expression (Fig. 2G,H).

**Figure 3:**
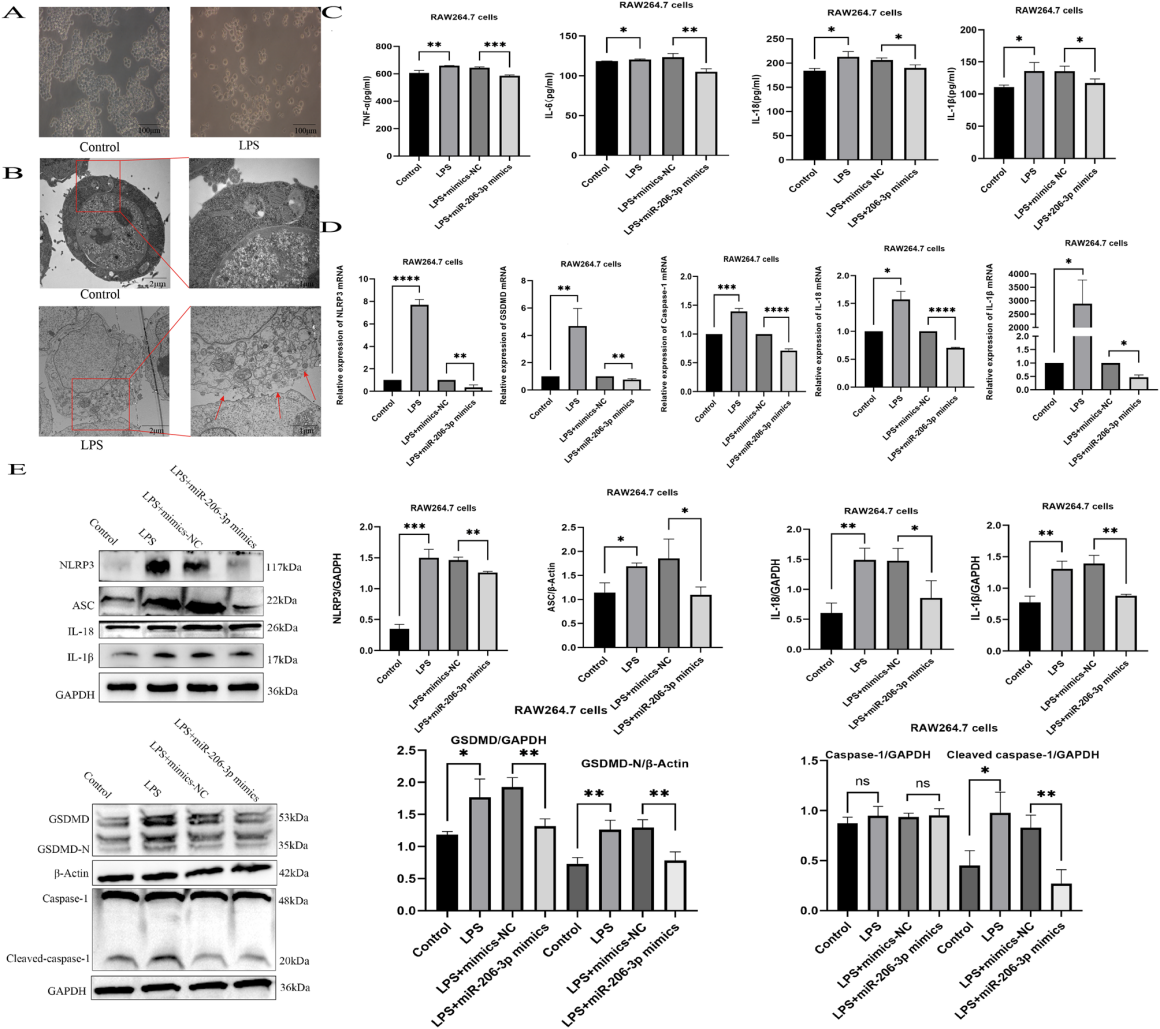
Overexpression of miRNA-206-3p inhibits LPS-induced inflammatory response and pyroptosis. (A) RAW264.7 cells morphology after treatment with 10 μg/mL LPS for 24 h. (B) Transmission electron microscopy analysis of the ultrastructure of RAW264.7 cells treated with 10 μg/mL LPS for 24 h; red arrow perforated cell membrane. (C) TNF-α, IL-6, IL-18 and IL-1β levels in RAW264.7 cells were measured after LPS challenge (n = 3/group). (D) mRNA levels of *NLRP3*, *GSDMD*, *caspase-1*, *IL-18*, and *IL-1β* in RAW264.7 cells challenged with LPS (n = 3/group). (E) Abundance of NLRP3, ASC, caspase-1, cleaved caspase-1, GSDMD, GSDMD-N, IL-18, and IL-1β proteins in RAW264.7 cells challenged with LPS (n = 3/group). Data represent the mean ± SD of three independent experiments. The blots were cut prior to hybridization with antibodies. *P < 0.05, **P < 0.01, ***P < 0.001, ****P < 0.0001.

Pyroptosis is a key pathological feature of ALI^26^. LPS-induced RAW264.7 cells showed morphological abnormalities and cell swelling under an optical microscope (Fig. 3A). Transmission electron microscopy was used to observe the ultrastructure of cells and determine whether miRNA-206-3p inhibited the pyroptosis in LPS-induced RAW264.7 cells. Results show that the RAW264.7 cells in the LPS group exhibited a pyroptotic state with irregular cell morphology, mitochondrial swelling, multiple cell membrane breakage, and release of cell contents into the extracellular space (Fig. 3B). At the same time, we also detected the changes of cytokines TNF-α, IL-6, IL-18, and IL-1β among each group. Compared to the normal group, these cytokines increased significantly in the LPS groups, but these effects were reversed by overexpressing miRNA-206-3p (Fig. 3C).

**Figure 4:**
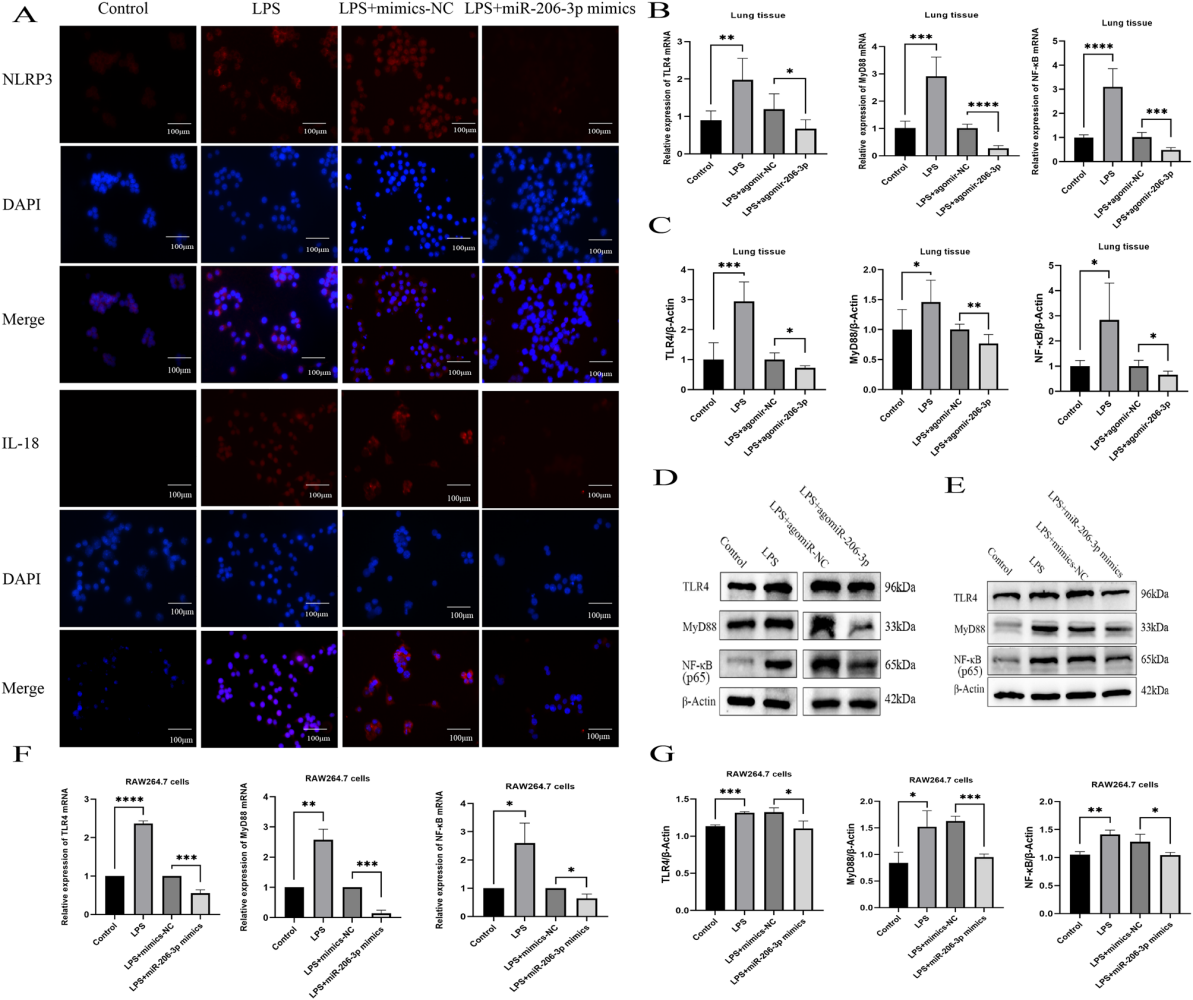
The miRNA-206-3p alleviates inflammation and pyroptosis by regulating the TLR4/MyD88/ NF-κB pathway. (A) Expression of pyroptosis markers (NLRP3 and IL-18) measured by immunofluorescence. (B–D) qRT-PCR and western blotting analysis of TLR4, MyD88, and NF-κB expression in LPS-induced mice (n = 6/group). (E– G) qRT-PCR and western blotting analysis of TLR4, MyD88, and NF-κB expression in LPS-induced RAW264.7 cells (n = 3/group). Data represent the mean ± SD of three independent experiments. The blots were cut prior to hybridization with antibodies. *P < 0.05, **P < 0.01, ***P < 0.001, ****P < 0.0001.

Previous research has established the significant roles of NLRP3, caspase-1, cleaved caspase-1, GSDMD, GSDMD-N, IL-18, and IL-1β in the development and progression of pyroptosis^27^. Thus, we examined their expression at the transcriptional and translational levels using qRT-PCR, immunofluorescence, and western blotting. The expressions of these pyroptosis-related genes increased significantly in the LPS group alone compared with controls (Fig. 3D,E). Immunofluorescence analysis revealed similar results for NLRP3 and IL-18 (Fig. 4A). These findings indicate that upregulating miRNA-206-3p mitigates LPS-induced pyroptosis in RAW264.7 cells.

### miRNA-206-3p mitigates LPS-induced ALI via blocking the TLR4/NF-κB/NLRP3 pathway

The TLR4/MyD88/NF-κB pathway has been identified as a pivotal regulator of the inflammatory process in inflammation-related diseases^28,29^. Hence, we further explored the regulatory effect of miRNA206-3p on the activation of this pathway in LPS-induced ALI models. In vivo, the LPS group exhibited significantly elevated TLR4, MyD88, and NF-κB expression levels compared to the control group (Fig. 4B–D). In contrast, their expression decreased in the LPS + agomir-206-3p group. Similarly, the expression levels of *TLR4*, *MyD88*, and *NF-κB* were higher in LPS-induced RAW264.7 cells; this effect was reversed following miRNA-206-3p mimic treatment (Fig. 4E–G).

Combined with the NLRP3 results, these findings suggest that the TLR4/MyD88/NF-κB/NLRP3 pathway was activated and contributed to the development of LPS-induced ALI. Meanwhile, the beneficial effects elicited by miRNA-206-3p overexpression regarding improved lung tissue pathological damage, reduced edema, and attenuated release of pro-inflammatory cytokines may be achieved by regulating this pathway.

### miRNA-206-3p directly binds toTLR4

**Figure 5:**
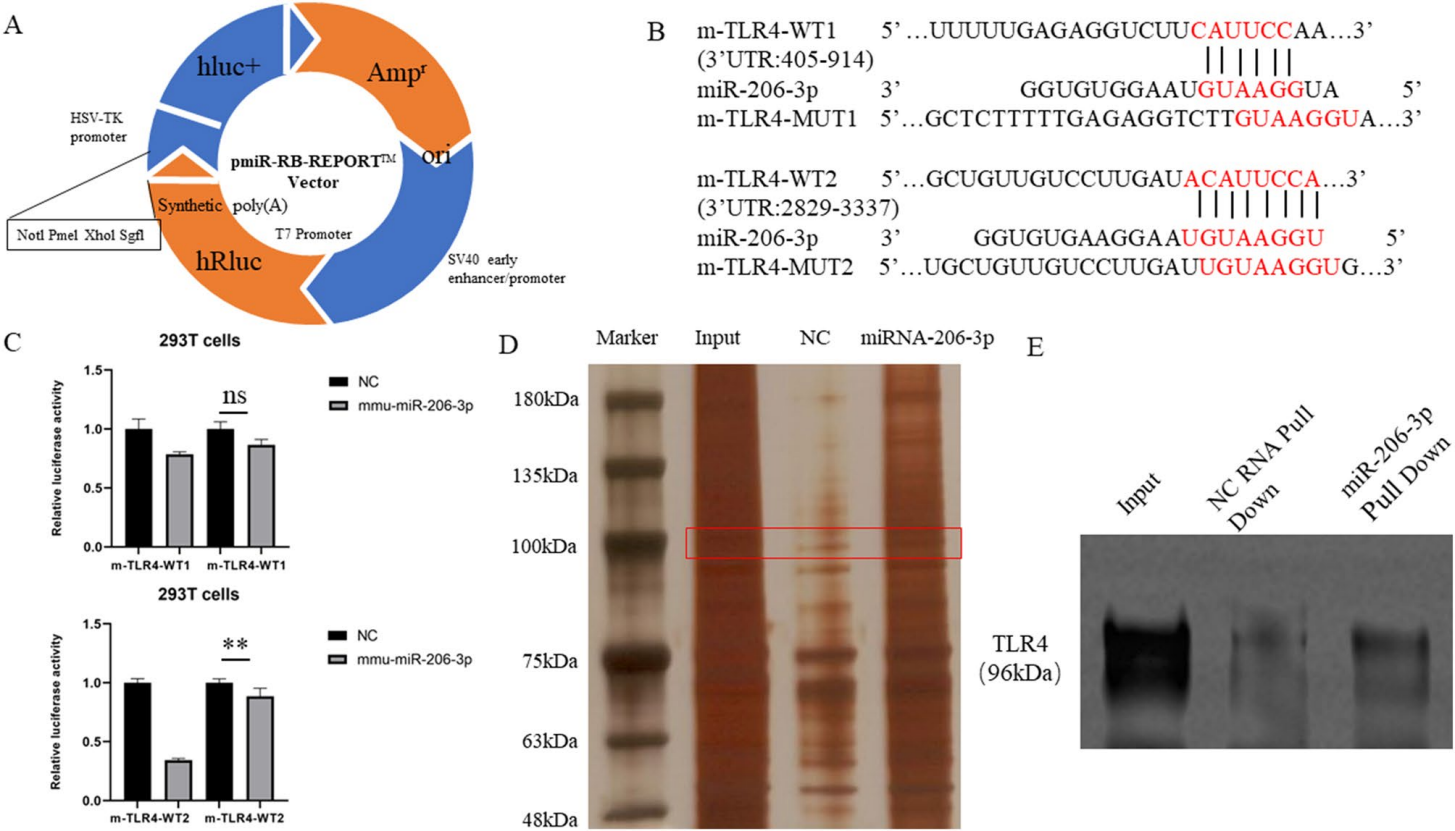
TLR4 is a direct target of miRNA-206-3p. (A, B) The two putative binding sites of miRNA-206-3p and TLR4 were shown. (C) Fluorescence expression of m-TLR4-MUT1 in 293T cells co-transfected with mmu-miRNA-206-3p, m-TLR4-WT1, m-TLR4-MUT2 or m-TLR4-WT2 (n = 3/group). (D) RNA pull-down silver staining results. (E) RNA pull-down Western blotting results. Data represent the mean ± SD of three independent experiments. **P < 0.01.

To characterize the molecular mechanism underlying the effects elicited by miRNA-206-3p, the TargetScan (http://www.targetscan.org) database was used to identify specific target molecules of miR-206-3p. Based on the prediction results, TLR4 was considered a strong candidate (Fig. 5A). TLR4 acts as a mediator in microbial infection, immune responses, and inflammatory reactions and has a key role in ALI development^30^. Additionally, TLR4 promotes pyroptosis in alveolar macrophages and leads to lung inflammation through an autocrine mechanism^31^. Consequently, we explored the novel role of TLR4. Complementary sequences between miRNA-206-3p and the 3'-UTR of TLR4 were detected, with two binding sites identified (Fig. 5B). Therefore, we constructed vectors carrying the 3'-UTR of TLR4 and miRNA-206-3p mimics, which were co-transfected into 293T cells. The dual-luciferase reporter system results confirmed the validity of one of the two miRNA206-3p binding sites on TLR4 (Fig. 5C). RNA pull-down assays confirmed that TLR4 was the direct target gene of miRNA-206-3p (Fig. 5D,E). Indeed, *TLR4* expression was closely related to that of miRNA-206-3p. Moreover, TLR4 expression was elevated in the LPS-induced groups in vitro and in vivo (Fig. 4B–G). Meanwhile, treatment with agomir-206-3p or miRNA-206-3p mimics downregulated TLR4 expression. These results indicate that miRNA-206-3p exerts anti-inflammatory and antipyroptotic effects by directly targeting TLR4.

## Discussion

The anti-inflammatory and anti-pyroptotic properties of miRNA-206-3p have received particular attention in recent years. In our study, the present results verified the abnormal expression of miRNA-206-3p in LPS-induced ALI. Moreover, the protective effect of miRNA-206-3p overexpression on LPS-induced ALI in mice was confirmed through various experiments, such as lung ultrasound, histopathology, and ELISA. Mechanistically, miRNA-206-3p targets TLR4 to regulate the classical TLR4/MyD88/NF-κB/NLRP3 inflammatory pathway and may have major scientific value as a new therapeutic target for ALI.

**Figure 6:**
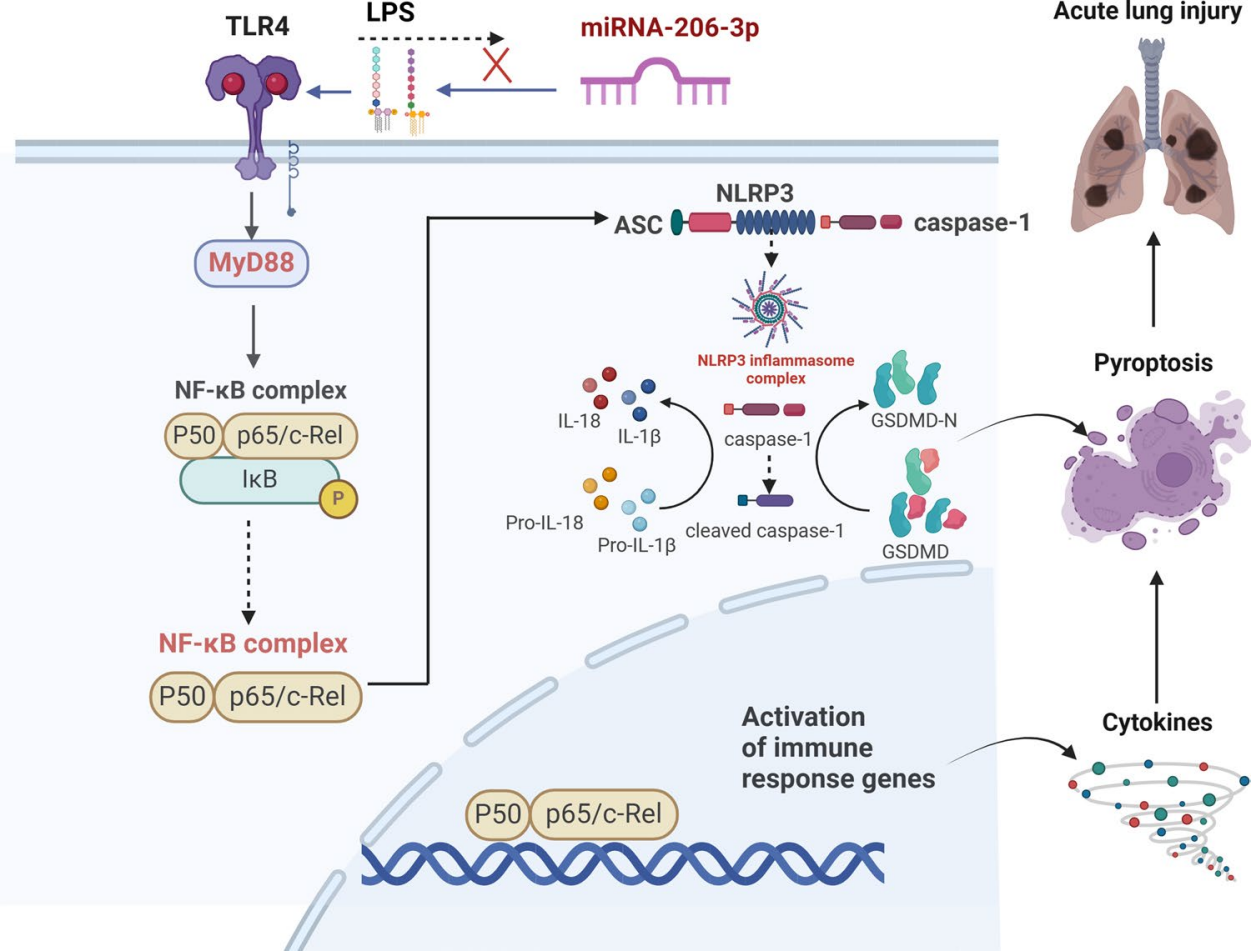
Scheme summarizing the protective effects of miRNA-206-3p on LPS-induced acute lung injury by inhibiting TLR4/NF-κB/NLRP3 activation. LPS can induce NF-κB activation through TLR4/MyD88 signaling. Once the pathway is activated and IκB is degraded, NF-κB translocates to the nucleus and induces the transcription of target genes, including *NLRP3*, *GSDMD*, and *ASC*; Produced NLRP3 assembles with ASC and pro-caspase-1 forming multimeric inflammasome complex that results in the cleavage of pro-caspase-1 to caspase-1. Subsequently, the release of inflammatory factors, including TNF-α, IL-6, IL-18, and IL-1β, is activated, regulating inflammatory responses. However, miRNA-206-3p attenuates the release of pro-inflammatory cytokines and cell pyroptosis by inhibiting TLR4/NF-κB/NLRP3 activation.

MiRNAs can control inflammatory and immune responses by selectively targeting specific molecules that play crucial roles in key processes related to ALI^32^. For example, Li et al. suggested that plasma extracellular vesicles carrying miRNA-210-3p specifically target ATG7 to effectively regulate the activation of inflammatory and autophagic mechanisms in a sepsis-induced ALI model^33^. Similarly, Qiao et al. discovered the protective role of miRNA-145-5p in rats with ALI through active suppression of E26 transformation-specific proto-oncogene 2 (ETS2) expression and deactivation of the transforming growth factor 1 (TGF-1)/Smad signaling pathway^34^. Meanwhile, Khan et al. found that the lungs of an LPS-induced ALI models showed a significant increase in miRNA-34a expression. Overexpression of miRNA-34a worsened the lung injury phenotype, favored the pro-inflammatory M1 phenotype and inhibited M2 polarization. They also found that regulation of Kruppel-like factor 4 and macrophage polarization contributed to these effects. These findings suggest that targeting these factors could be an ideal therapeutic intervention for ALI/ARDS^35^. Several studies have reported that miRNAs may have great potential diagnostic and therapeutic value as biomarkers for certain diseases^36,37^ Multiple experimental models have consistently shown that miRNAs are expressed in a dysregulated manner during LPS-induced ALI and have significant roles in several pathophysiological processes, including inflammation, apoptosis, autophagy, and pyroptosis^38–40^. In the present study, confirming with qRT-PCR, we found that miRNA-206-3p was significantly dysregulated in ALI mice compared to the normal group.

miRNA-206-3p has been extensively studied due to its close association with tumorigenesis and tumor development. However, there is ongoing discourse regarding its impact on cancer formation, including its potential as a cancer promoter or inhibitor^41^. Additionally, the aberrant expression of miRNA-206-3p has recently garnered significant attention in research on inflammatory disease. Liang et al. identified miRNA206 as a potential biomarker for sepsis severity with a positive correlation detected between sepsis severity and serum miRNA-206 levels^42^. Dong et al. reported a protective role for miRNA-206 against myocardial inflammatory injuries induced by targeting the USP33, which may be a reliable therapeutic target^43^. Meanwhile, Zhou et al. found that the effects of miRNA-206-3p on connexin, ultimately improving lung permeability in sepsis-induced ALI^44^.Some widely used medications, including remifentanil, protect the heart against the damage caused by myocardial ischemia/reperfusion (I/R) by modulating the miRNA-206-3p^45^.

Herein, we found that enhanced expression of miRNA-206-3p improved LPS-induced ALI, as demonstrated by improved pulmonary ultrasound images and histopathological changes, as well as the reduction of the W/D ratio and inflammatory cytokines levels in the murine model. TNF-α and IL-6 are considered reliable and objective indicators of ARDS/ALI^46^. The absence of TNF-α in the local lung tissue substantially decreases lung tissue injury following hemorrhage priming for ALI^47^. Moreover, a study investigating human metapneumovirus infection in children, it was found that assessing the severity and prognosis of infection may be facilitated by examining IL-6 and TNF-α expression levels^48^.

Increasing evidence has shown that macrophages are closely associated with cell pyroptosis and that blocking macrophage pyroptosis may reduce inflammatory responses^49,50^. As a type of phagocytic cell in the innate immune system, macrophages are renowned for their ability to engulf and eliminate foreign invaders efficiently. The crucial and central role macrophages play in maintaining tissue equilibrium and responding to pathogenic stimuli is widely acknowledged in the field of immunology. During inflammation, immune cells produce a wide range of reactive oxygen and nitrogen species as a defense mechanism. However, an excessive response can induce cell damage and potentially lead to cell death^51^. Kang et al. identified typical cell perforation pyrogenic characteristics in LPS-stimulated RAW264.7 cells, indicating the successful development of LPS-induced pyroptosis model *in vitro*^52^. Similarly, in the current study, following with LPS treatment, RAW264.7 cells exhibited typical features of pyroptosis, including cell swelling and membrane perforation. Considering that miRNAs have been broadly reported as participating in pyroptosis regulation, we propose that the abnormal expression of miRNA-206-3p may also be associated with the regulation of pyroptosis.

NLRP3, triggers caspase-1 activation in inflammation-activated macrophages, activating caspase-1 to cleaved-caspase-1, leading to the cleavage of GSDMD and the production of GSDMD-N fragments^53^. Subsequently, GSDMD-N molecules assemble into the plasma membrane to form pores, which enhance membrane permeability. This leads to the release of mature IL-18 and IL-1β, as well as the initiation of pyroptosis^54^. Therefore, we verified the expression of NLRP3 and IL-18 by using immunofluorescence and assessed the expression levels of NLRP3, GSDMD, caspase-1, IL-18, and IL-1β through qRT-PCR and western blotting. As expected, mRNA and protein levels of pyroptosis-related molecules were significantly altered. Additionally, we found that miRNA-206-3p overexpression effectively reduced the secretion of pro-inflammatory factors, namely TNF-α, IL-6, IL-18, and IL-1β in RAW264.7 cells. Hence, miRNA-206-3p improves ALI by blocking inflammation and pyroptosis. However, the network by which miRNA-206-3p regulated the inflammatory response and macrophage pyroptosis remained unclear.

In inflammatory diseases, the activation of the TLR4/MyD88/NF-κB pathway mediates inflammatory responses, while loss of TLR4 may confer a benefit in LPS-associated ALI^55,56^. Notably, miRNA-206-3p also affects the expression of TLR4. For example, Zhang et al. found that remifentanil protected myocardial I/R injury by modulating the TLR4/NF-κB signaling pathway. Simultaneously, they found that the defense mechanism involved miRNA-206-3p, which plays a significant role in downregulating the expression of TLR4^45^. Therefore, in the current study, we sought to ascertain the potential protective function of miRNA-206-3p against LPS-induced ALI by specifically targeting TLR4. Indeed, the present results showed that miRNA-206-3p directly binds to TLR4 by targeting the 3′-UTR in vitro.

However, there are certain limitations in this study. First of all, although other studies have demonstrated the abnormal expression of miRNA-206-3p in sepsis patients^42^, this study did not collect large, multi-center clinical samples for specific verification. Second, miRNA-206-3p has also been found to be related to apoptosis^57^ and autophagy^58^; although pyroptosis is closely related to apoptosis, this study did not explore the exploration of the relationship between these programmed death modes.

In conclusion, our results demonstrated a noteworthy decrease in the expression level of miRNA-206-3p in ALI mice. Moreover, miRNA-206-3p overexpression protects against ALI by suppressing inflammation and pyroptosis. This proactive mechanism involves modulating the TLR4/NF-κB/NLRP3 pathway by directly targeting TLR4 (Fig. 6). Hence, the results of this study demonstrated a previously undisclosed function of miRNA-206-3p in the development of ALI, shedding light on its pathological significance. In the future, an in-depth study of multiple cellular programmed death pathway mechanisms or encapsulation of miRNA and siRNA into nanomaterials may be exploited for new therapeutics. These novel findings can potentially contribute to the development of innovative therapeutic approaches for ALI.

### Supplementary Information

Supplementary Information.

## Data Availability

Online generated or analysed during this study are included in the article, the experimental data section to support the findings is included in a Supplementary information file, and additional experimental data available from the corresponding author on reasonable request.
