# Supplementary material for: miRNA-206-3p alleviates LPS-induced acute lung injury via inhibiting inflammation and pyroptosis through modulating TLR4/NF-κB/NLRP3 pathway
Source: Sci Rep. 2024 May 24;14:11860. doi: 10.1038/s41598-024-62733-5 (PMC11126654; doi:10.1038/s41598-024-62733-5)
Supplement: Supplementary file 1 — Supplementary Information. [file 41598_2024_62733_MOESM1_ESM.pdf]

Instructions

- To provide experimental efficiency, the blots were cut prior to hybridisation with antibodies during blotting.
- The code at the top left of all spots means the corresponding antibody and/or date of incubation, as follows:

Mark abbreviation	Antibody full name	Mark abbreviation	Antibody full name
NL	NLRP3	1b	IL-1β
ASC	ASC	T	TLR4
GS	GSDMD	N	NF-κB
C1	Caspase-1/cleaved caspase-1	M	MyD88
18	IL-18	G	GAPDH
A	β-Actin		

- The red boxes represent the original blots areas used in the manuscript.

NLRP3-117kDa

2023-04-02

Control, LPS, LPS+mimics-NC,LPS+miRNA-206-3p mimics

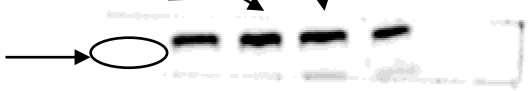

Marker
30kDa

GAPDH

Control, LPS, LPS+mimics-NC,LPS+miRNA-206-3p mimics

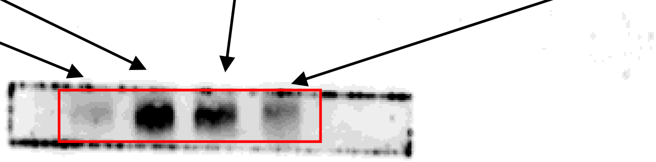

Marker
120kDa

NLRP3

NLRP3-117kDa

2023-04-04

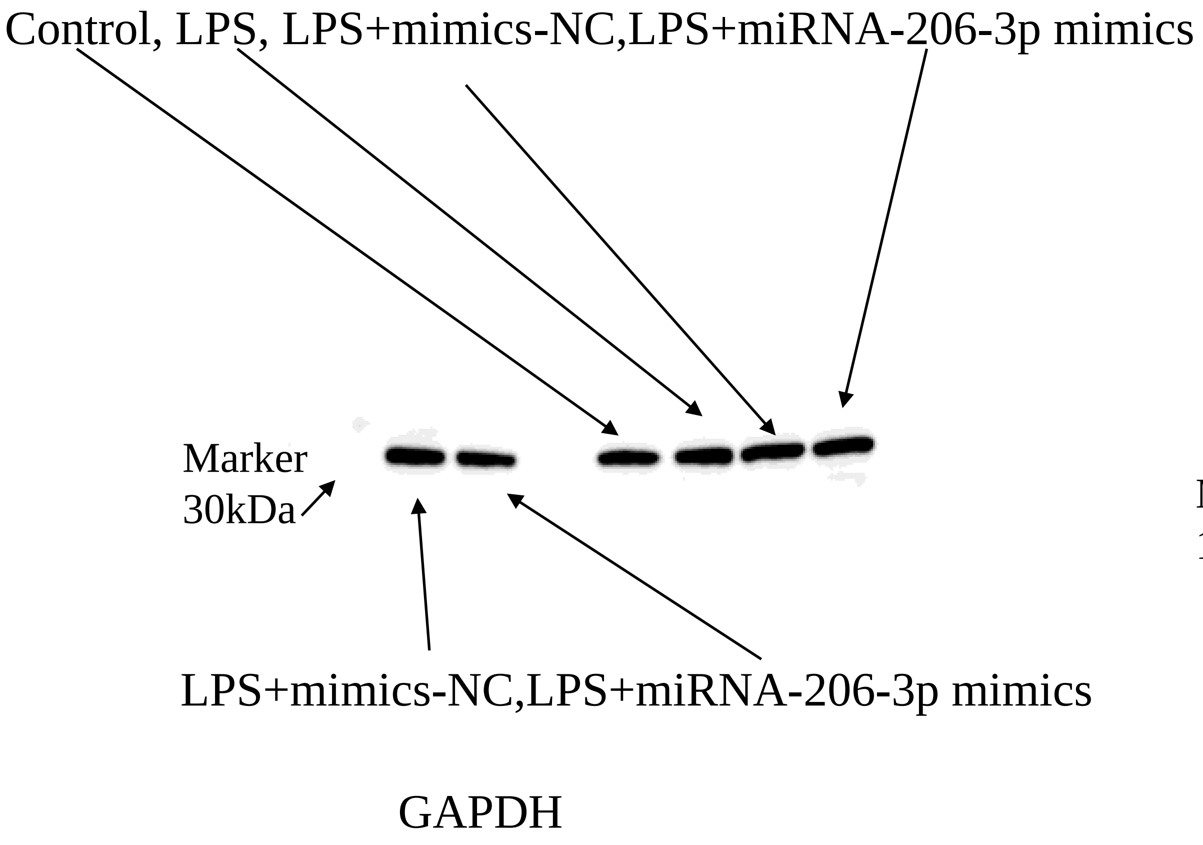

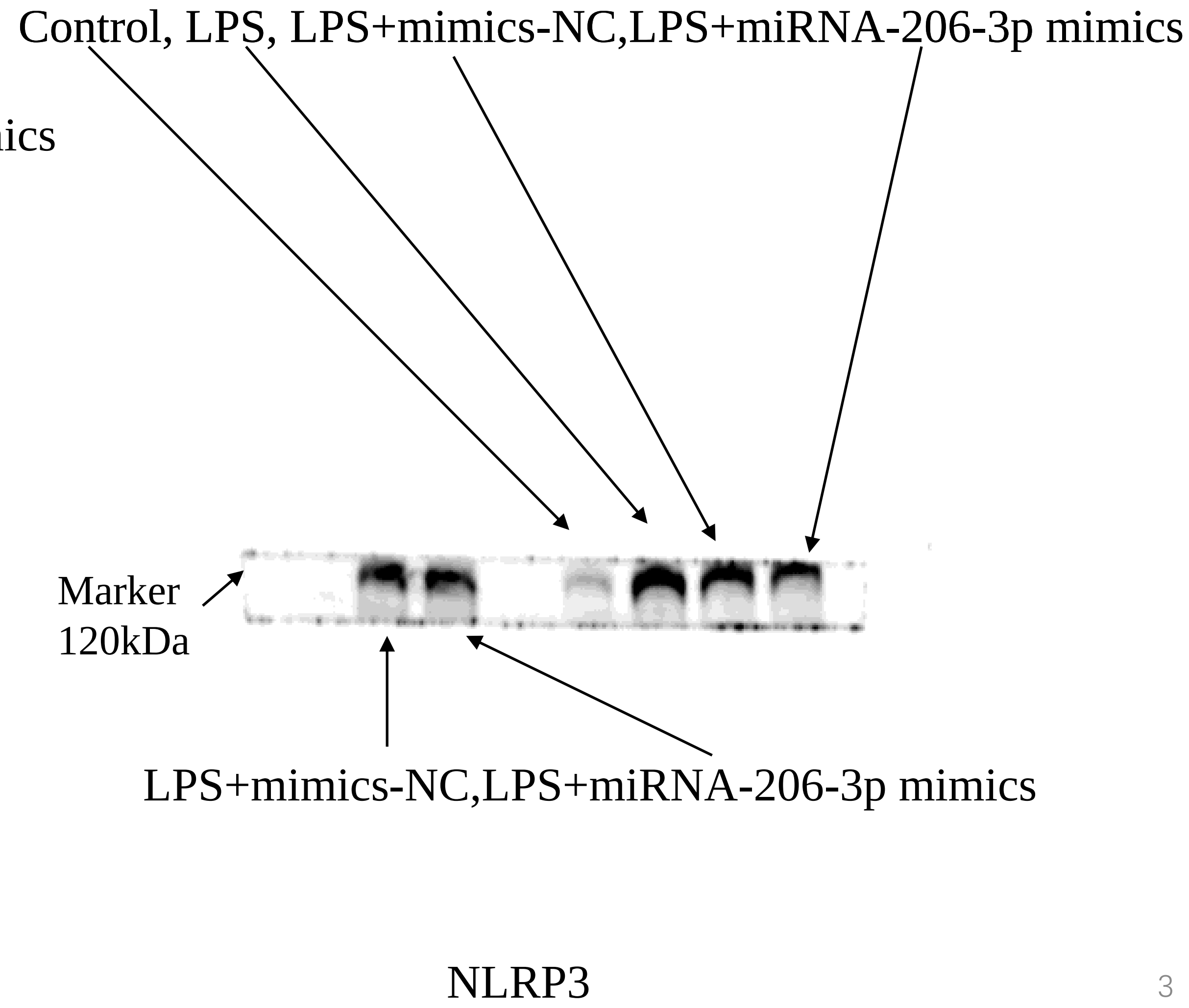

NLRP3-117kDa

2023-04-08

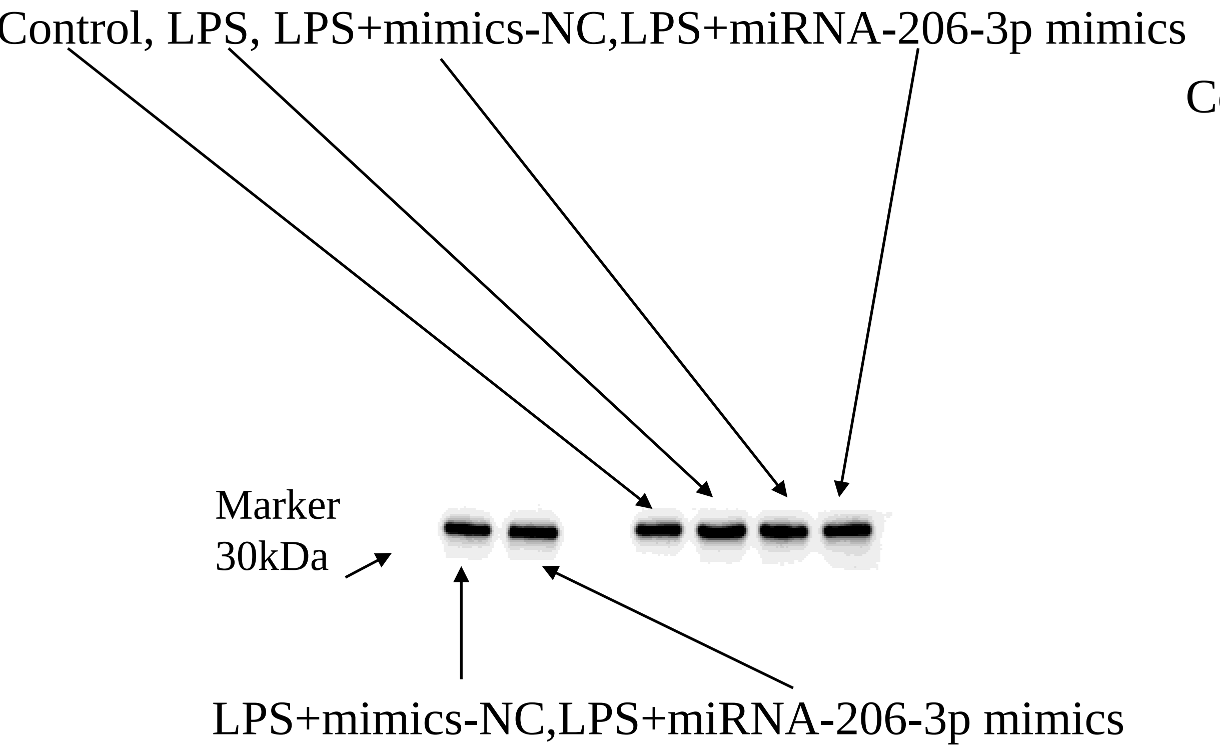

GAPDH

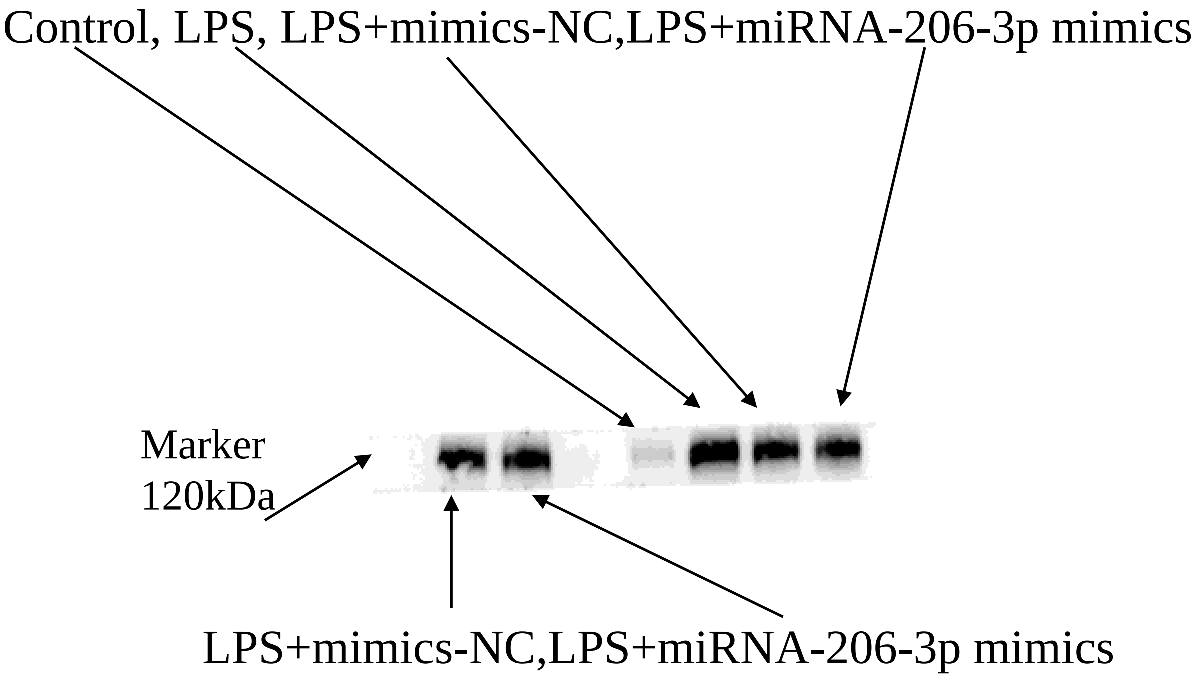

NLRP3

NLRP3-117kDa

2023-10-26

Control, LPS, LPS+mimics-NC,LPS+miRNA-206-3p mimics

120kDa

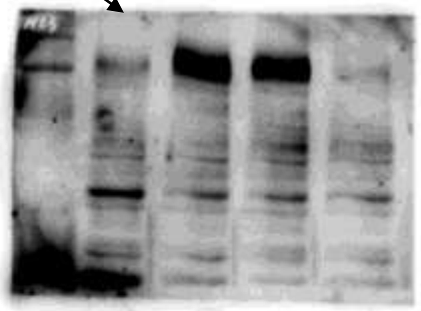

NLRP3

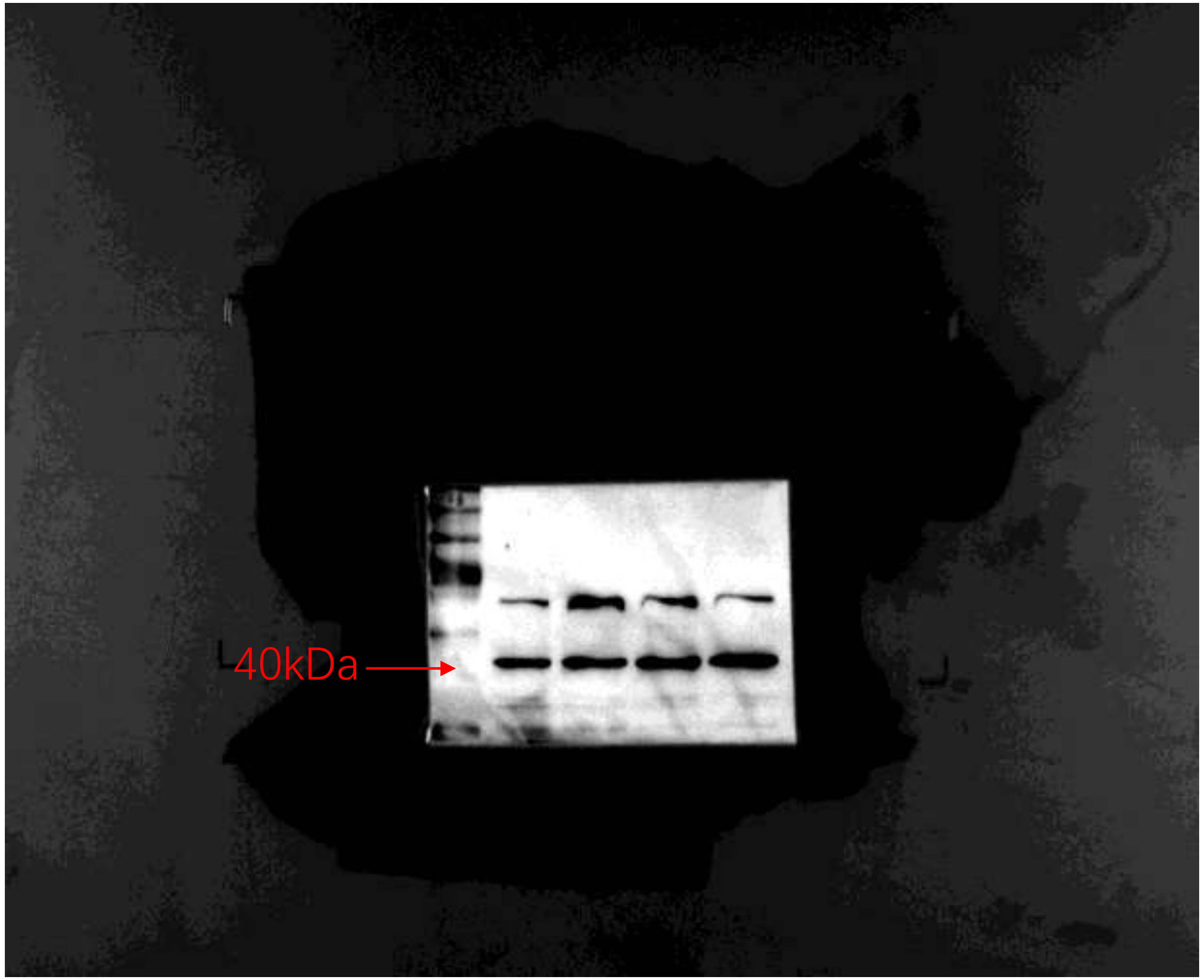

β-Acitin

ASC-22kDa

2023-12-22

Control, LPS, LPS+mimics-NC,LPS+miRNA-206-3p mimics

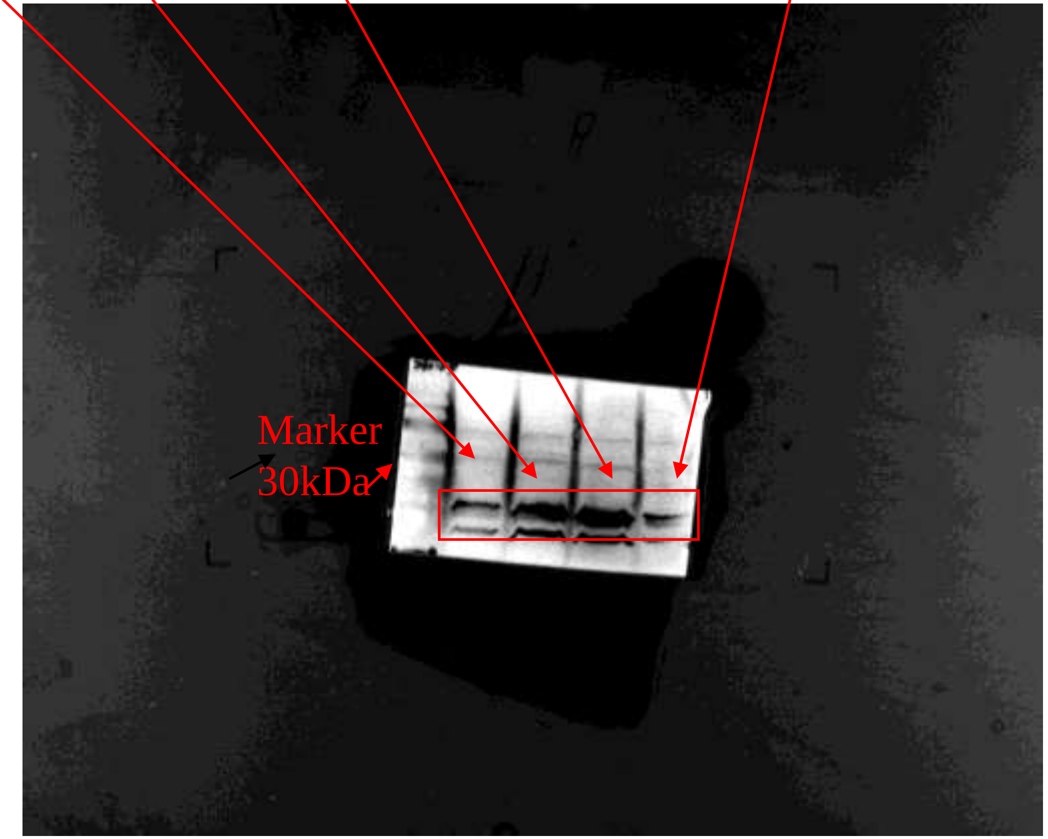

ASC

Control, LPS, LPS+mimics-NC,LPS+miRNA-206-3p mimics

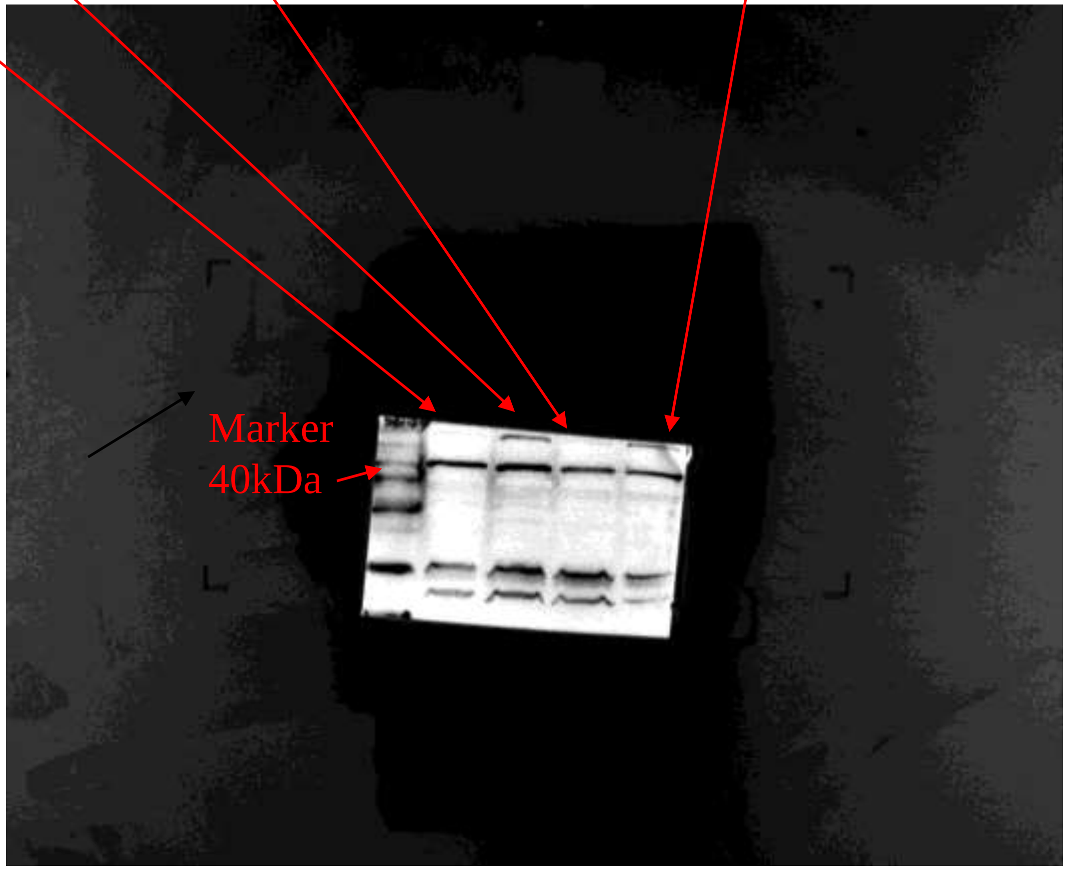

β-Actin

2023-12-22

ASC
22kDa

Control, LPS, LPS+mimics-NC,LPS+miRNA-206-3p mimics

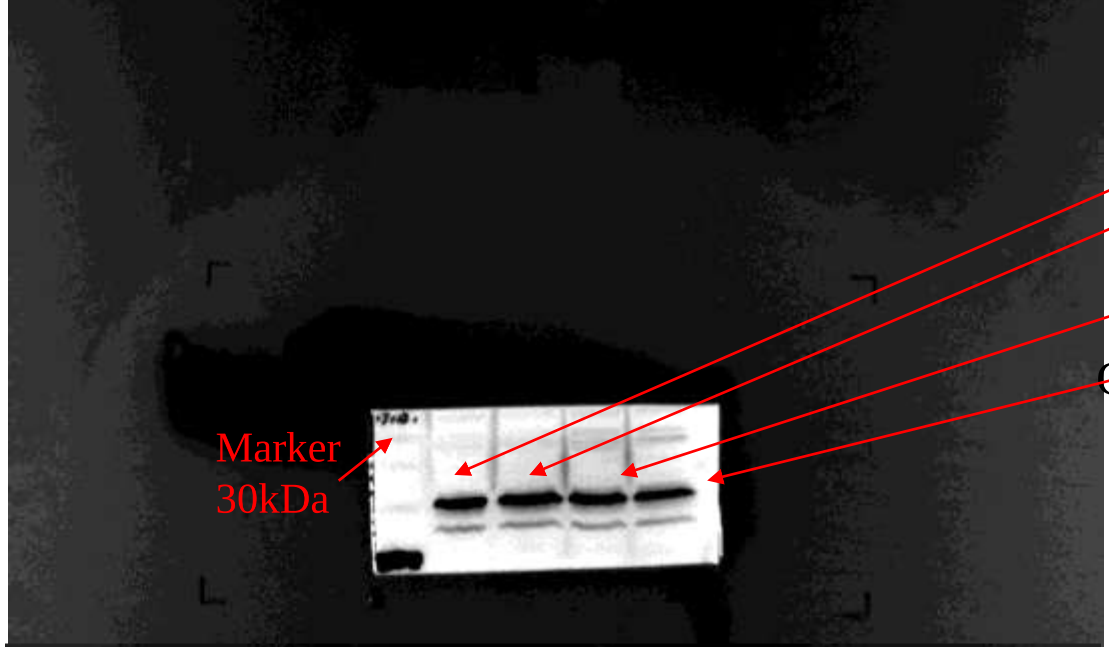

Control, LPS, LPS+mimics-NC,LPS+miRNA-206-3p mimics

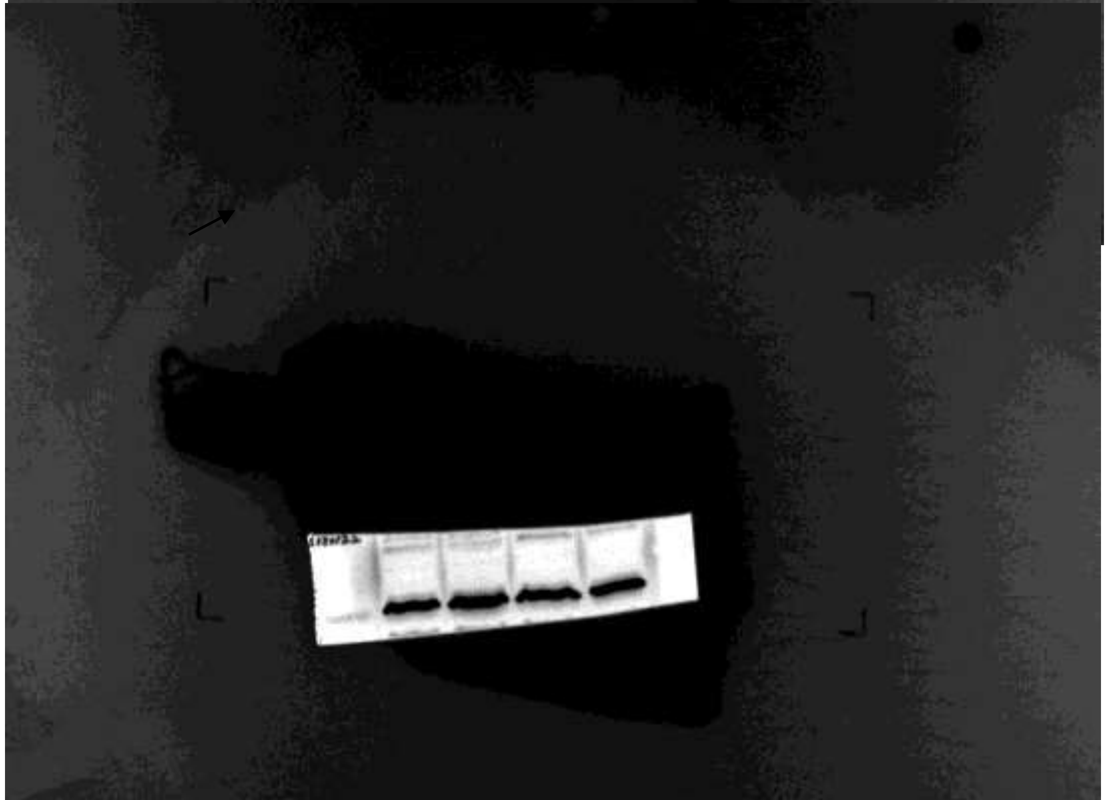

ASC

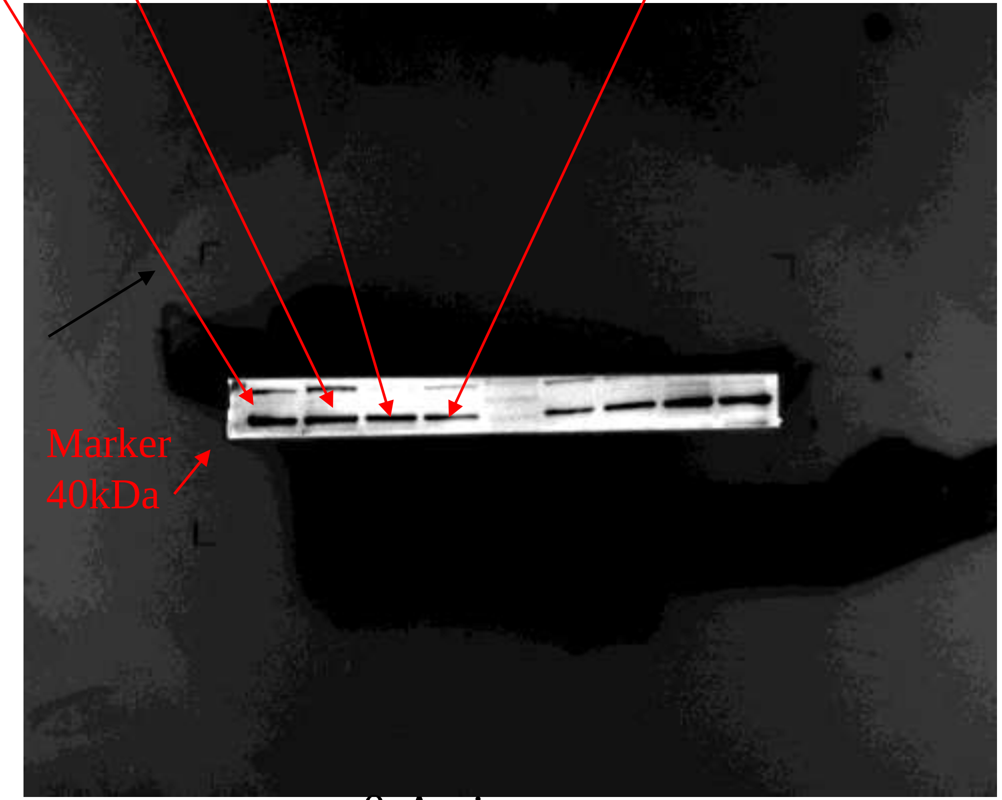

β-Actin

ASC-22kDa 2023-12-22

Control, LPS, LPS+mimics-NC,LPS+miRNA-206-3p mimics

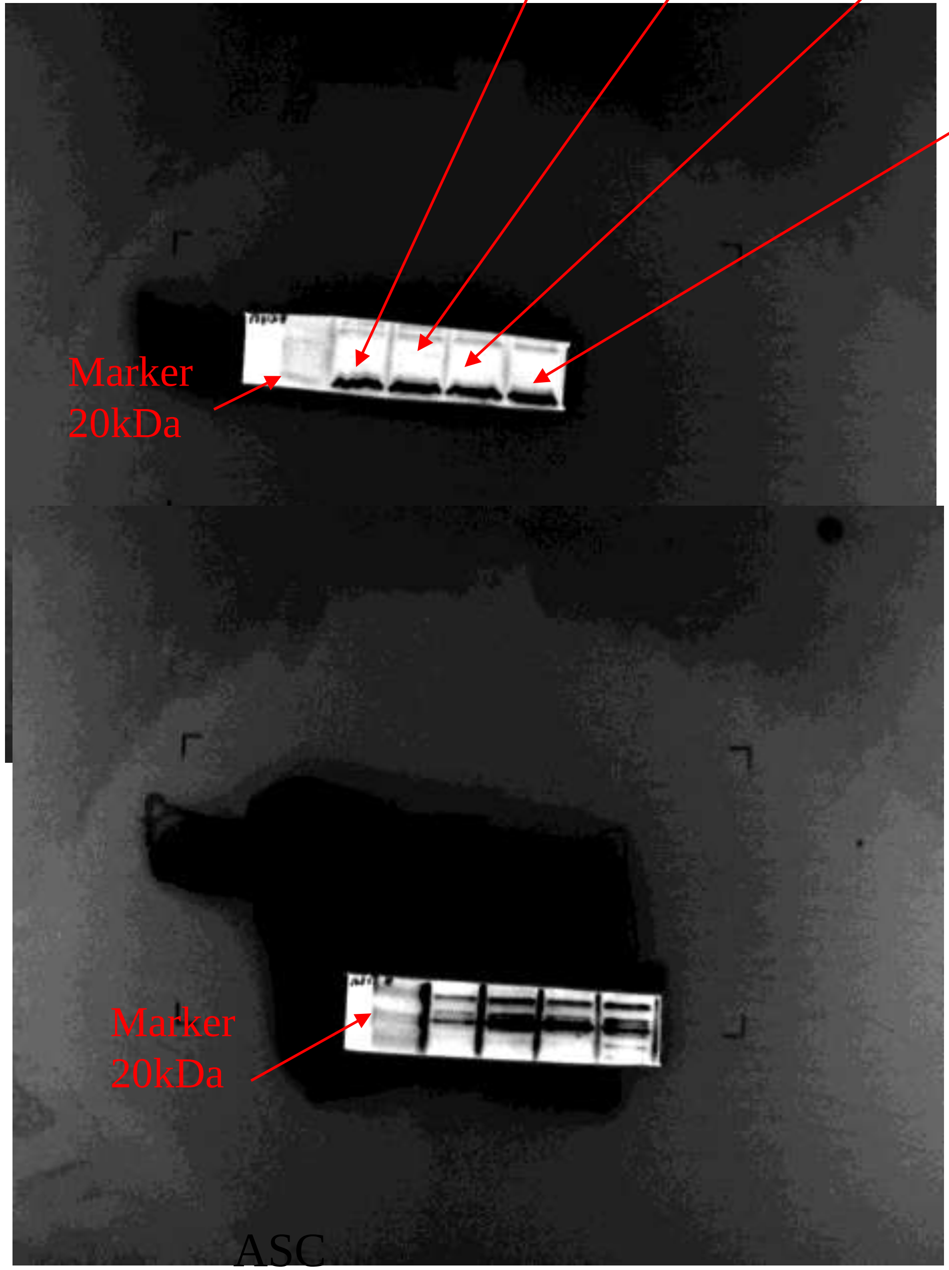

Control, LPS, LPS+mimics-NC,LPS+miRNA-206-3p mimics

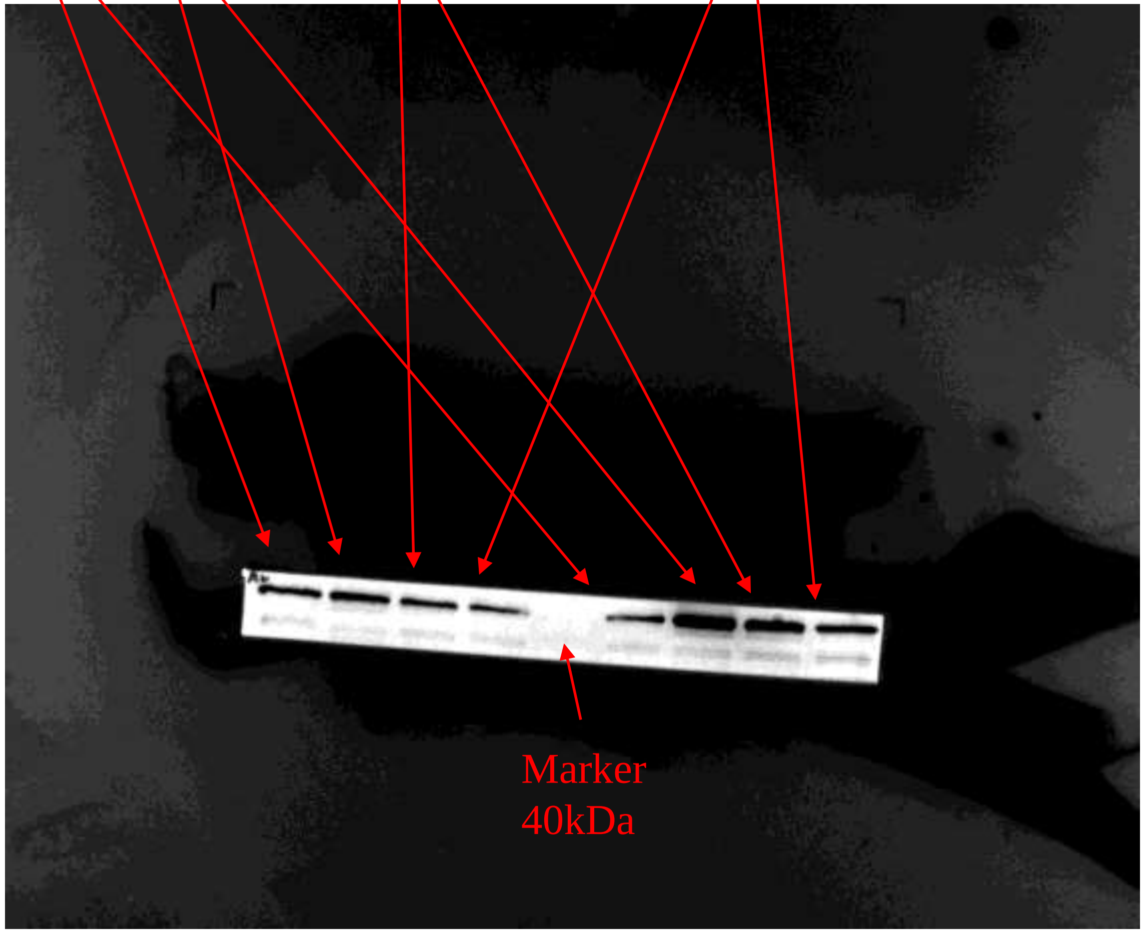

β-Actin

GSDMD/GSDMD-N-53/35kDa

2023-04-15

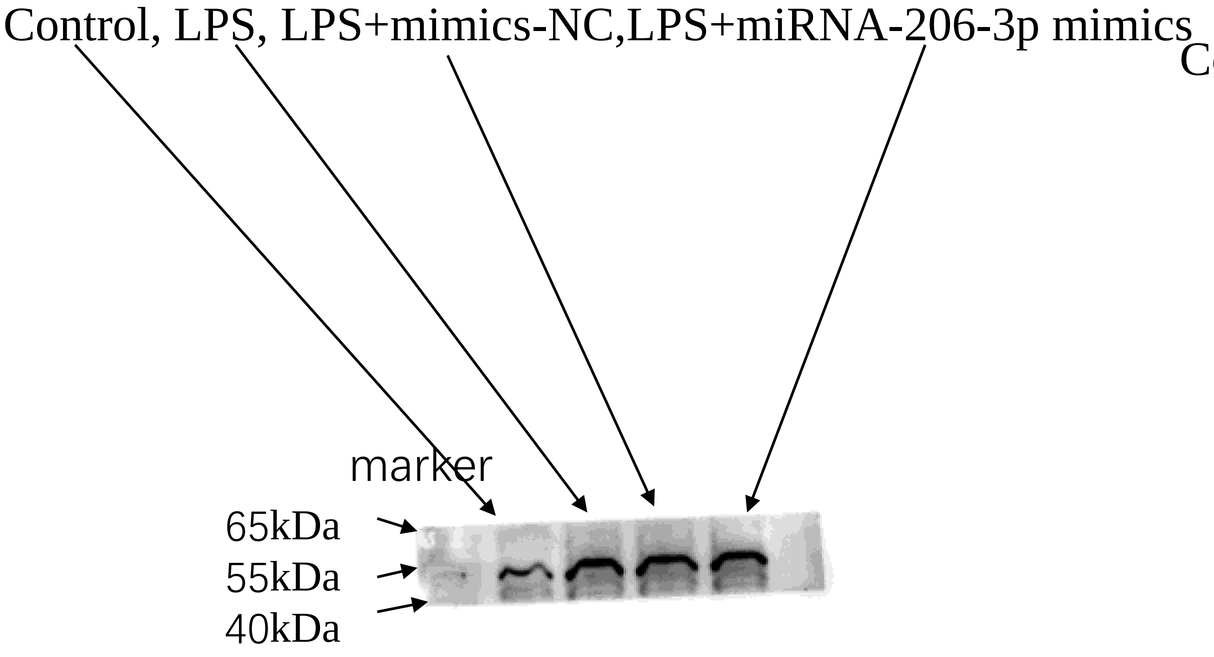

GSDMD 53kDa

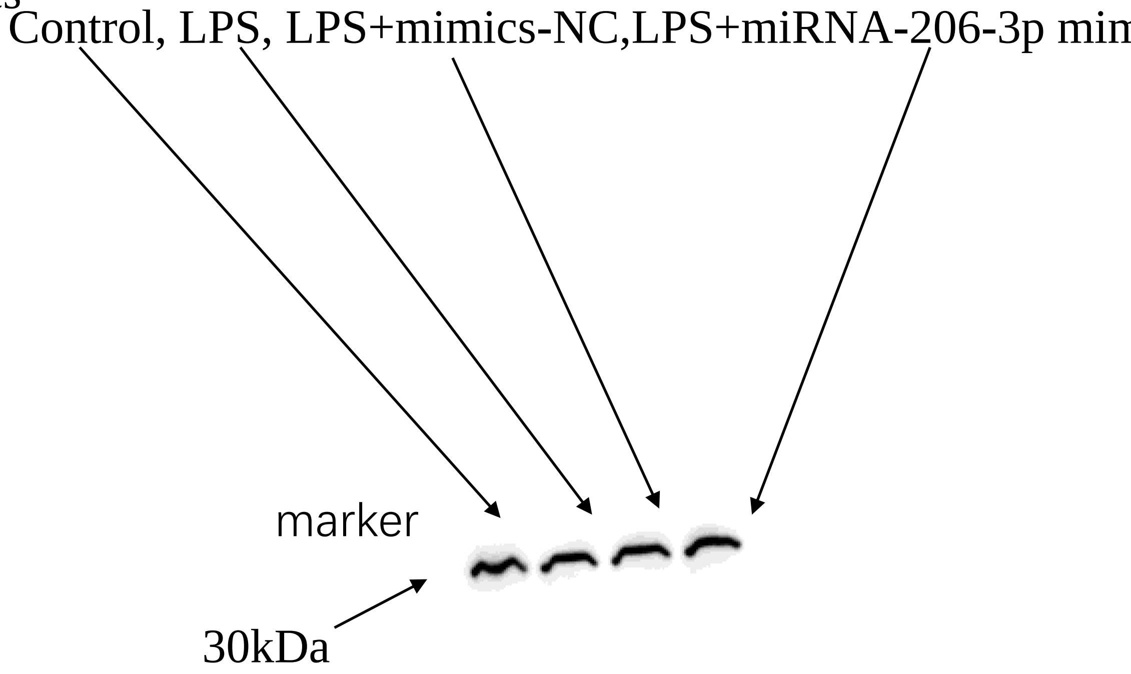

GAPDH

GSDMD/GSDMD-N-53/35kDa

2023-04-21

Control, LPS, LPS+mimics-NC,LPS+miRNA-206-3p mimics

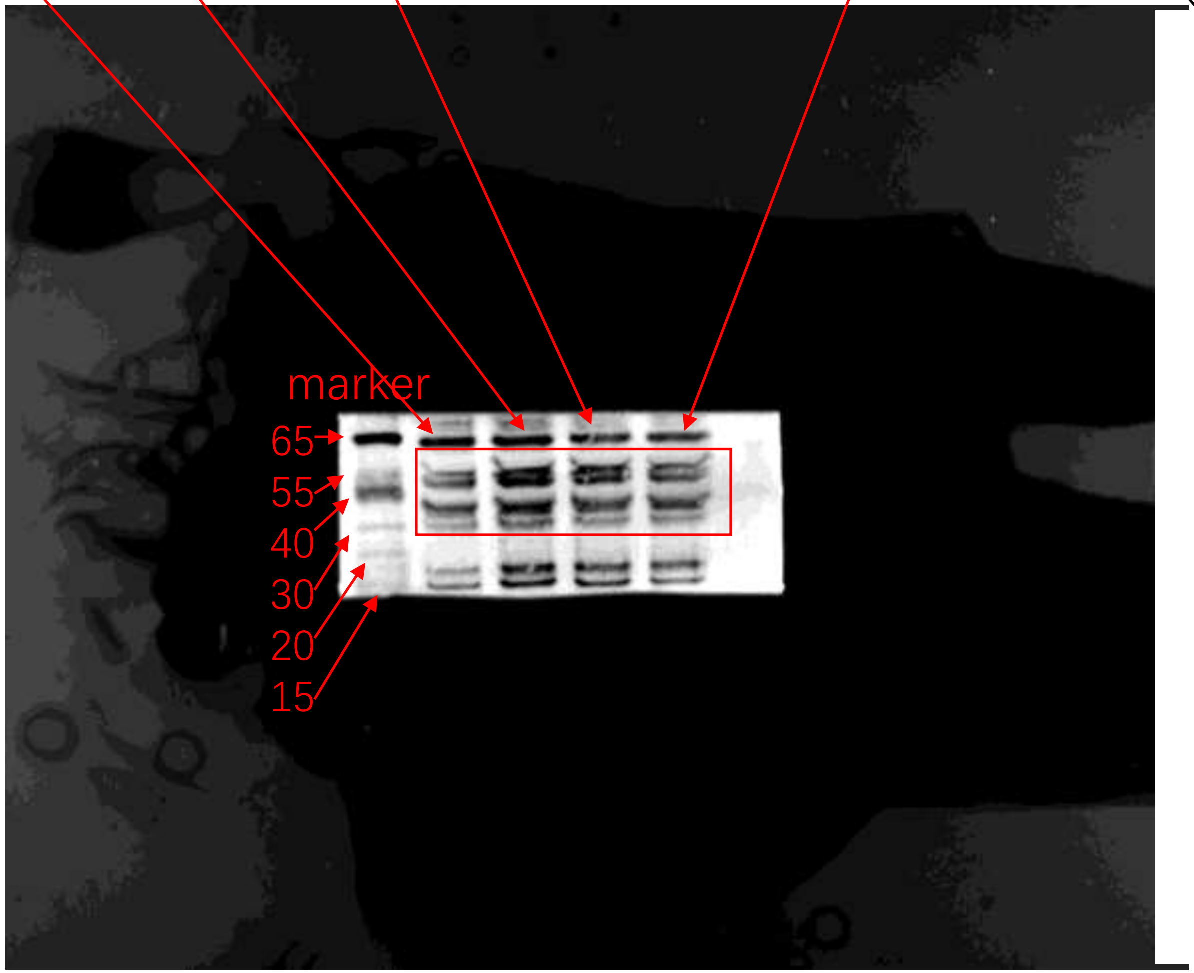

GSDMD/GSDMD-N

Control, LPS, LPS+mimics-NC,LPS+miRNA-206-3p mim

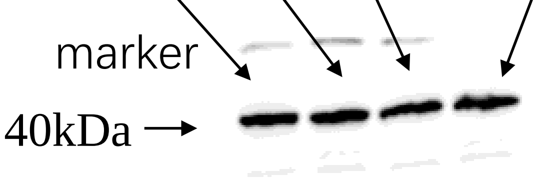

β-Actin

GSDMD/GSDMD-N-53/35kDa

2023-05-18

Control, LPS, LPS+mimics-NC,LPS+miRNA-206-3p mimics

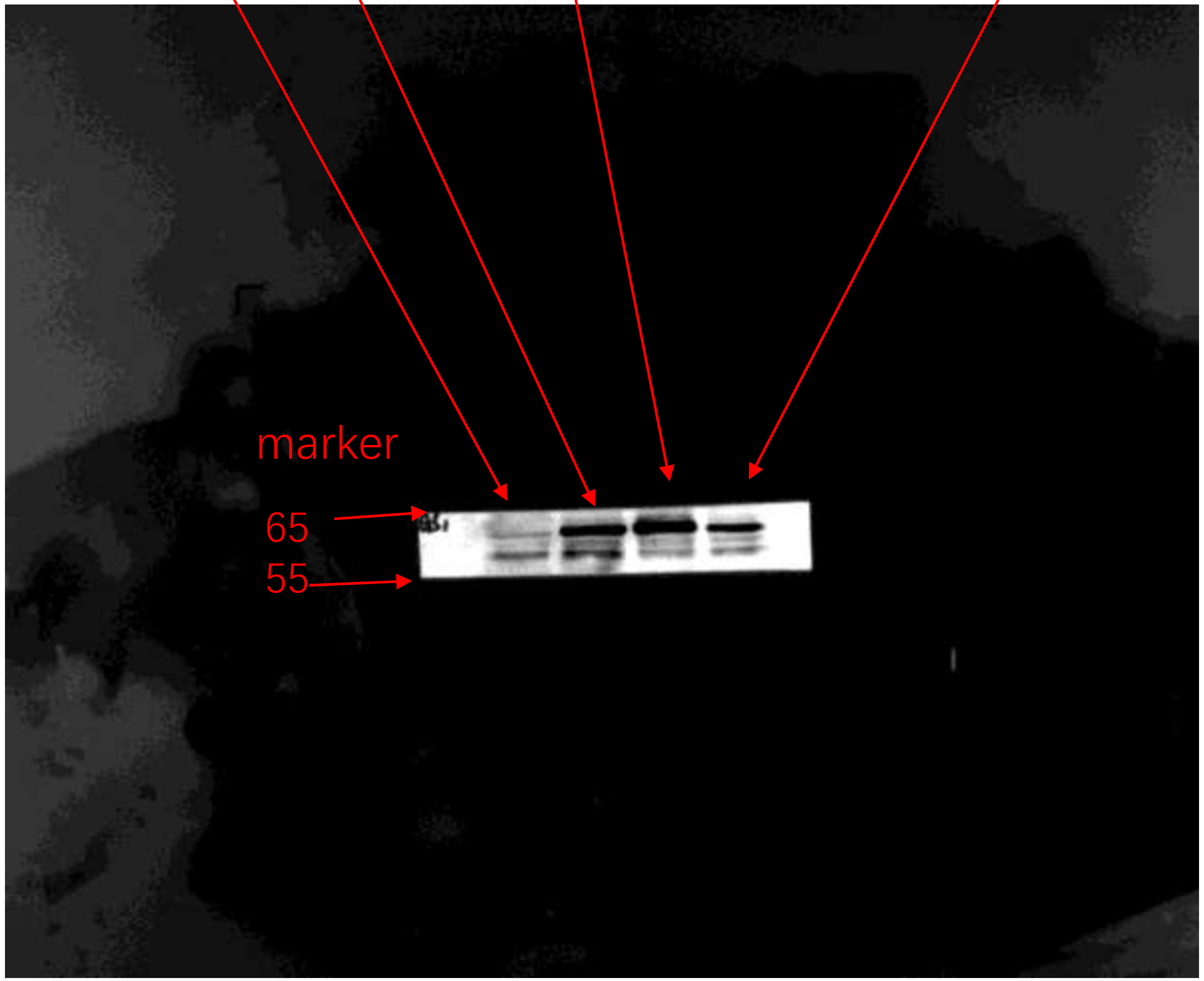

GSDMD 53

Control, LPS, LPS+mimics-NC,LPS+miRNA-206-3p mim

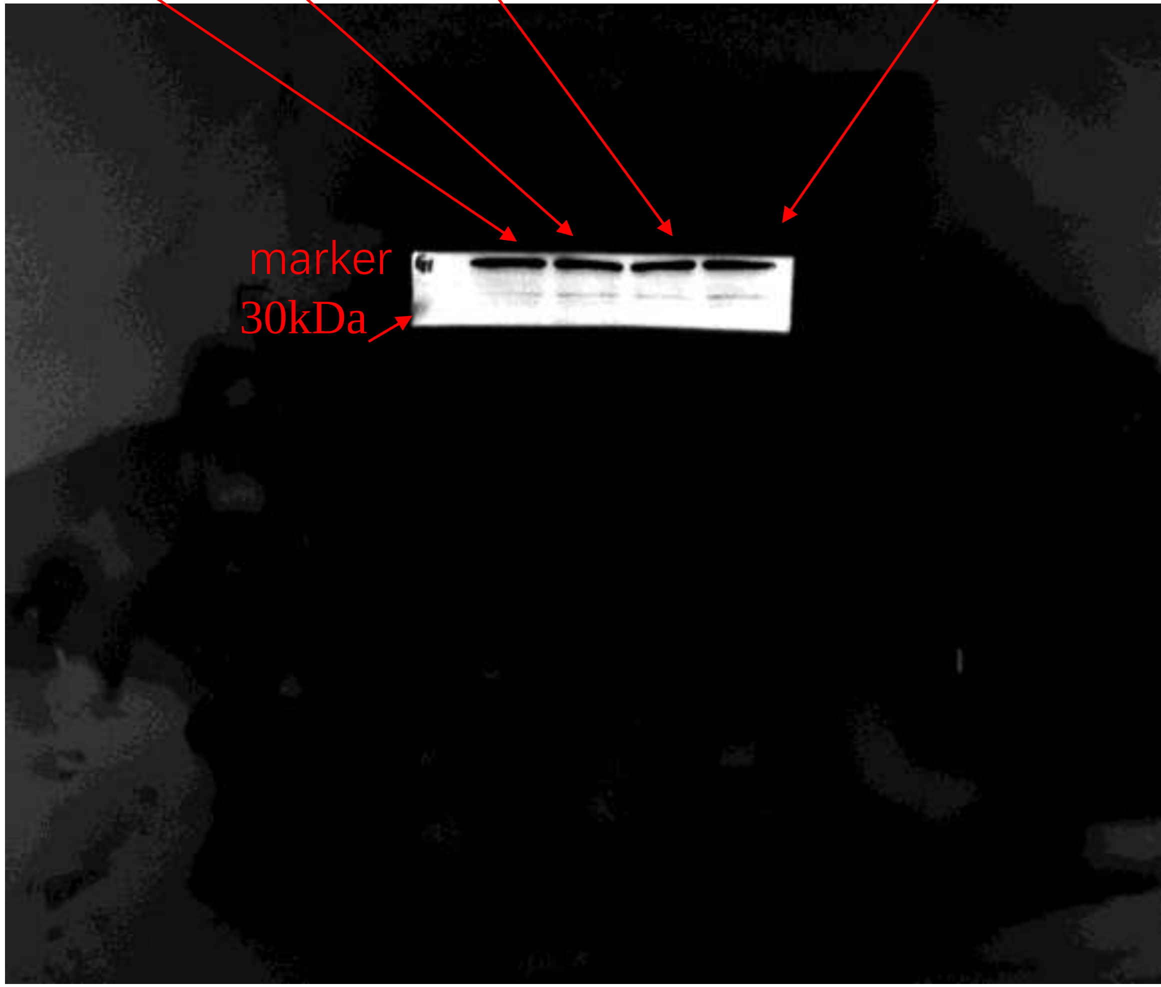

GAPDH

Caspase-1/Cleaved caspase-1:48/20kDa

2023-09-02-No.2

Control, LPS, LPS+mimics-NC,LPS+miRNA-206-3p mimics

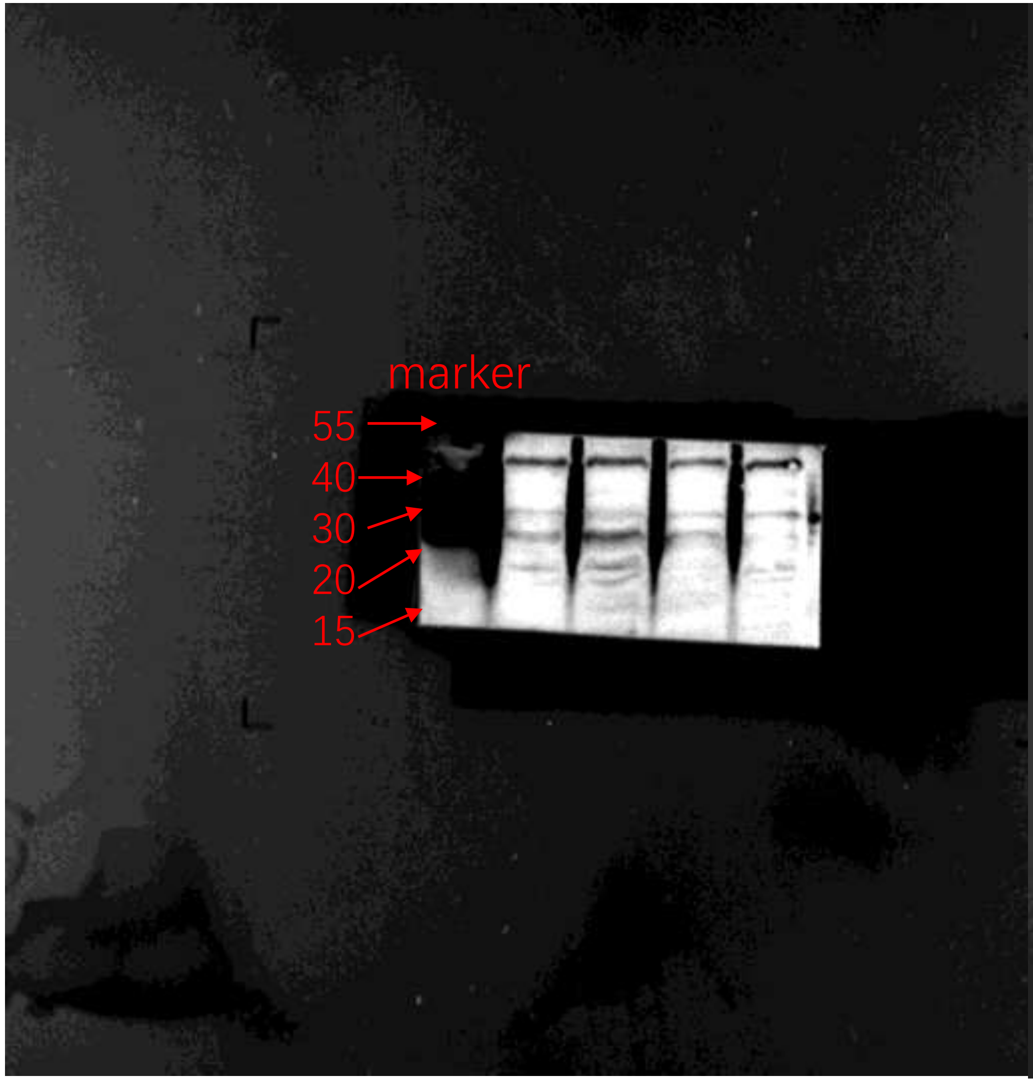

Caspase-1/Cleaved caspase-1

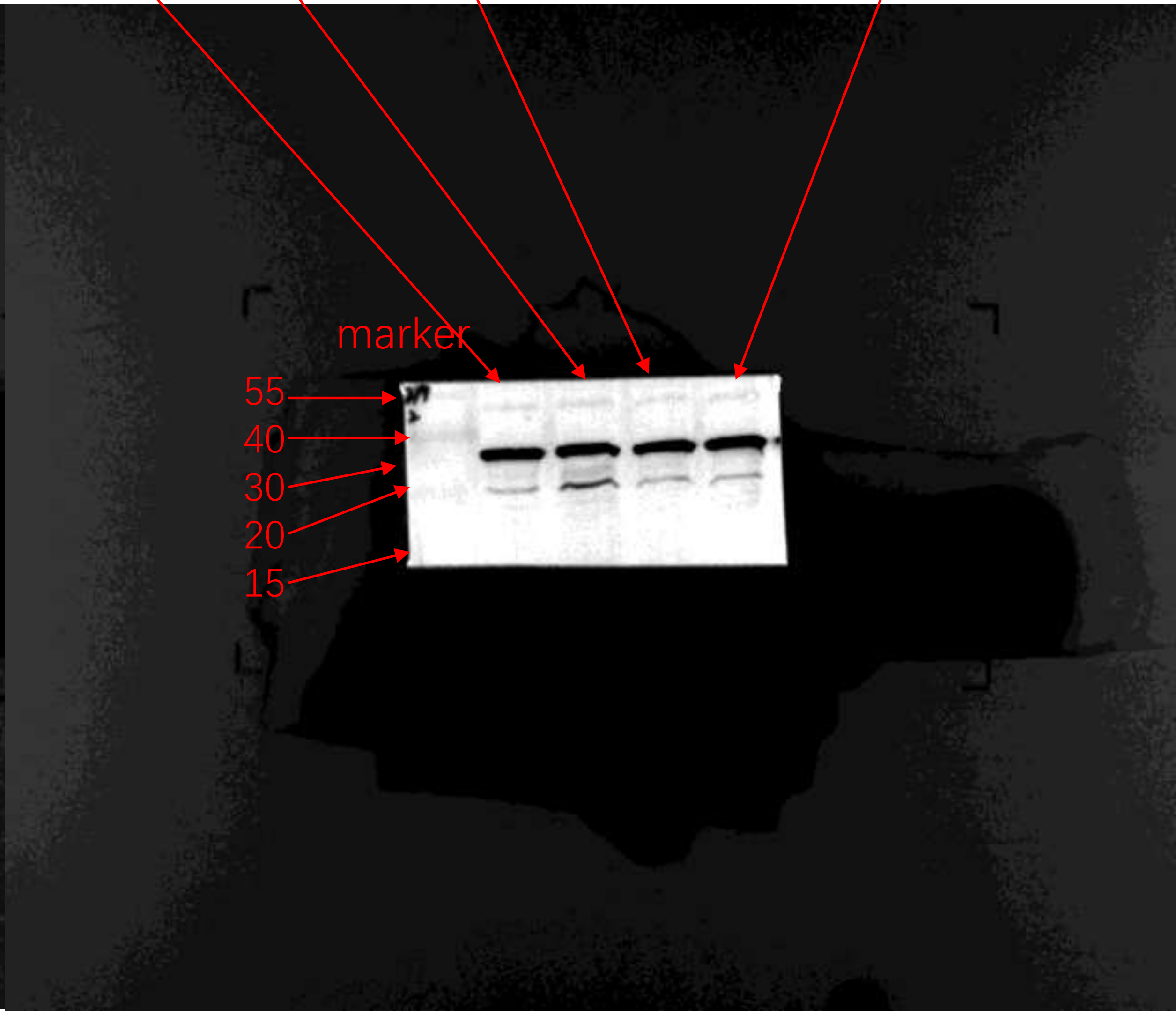

GAPDH

Caspase-1/Cleaved caspase-1:48/20kDa
2023-09-04-No.3

Control, LPS, LPS+mimics-NC,LPS+miRNA-206-3p mimics

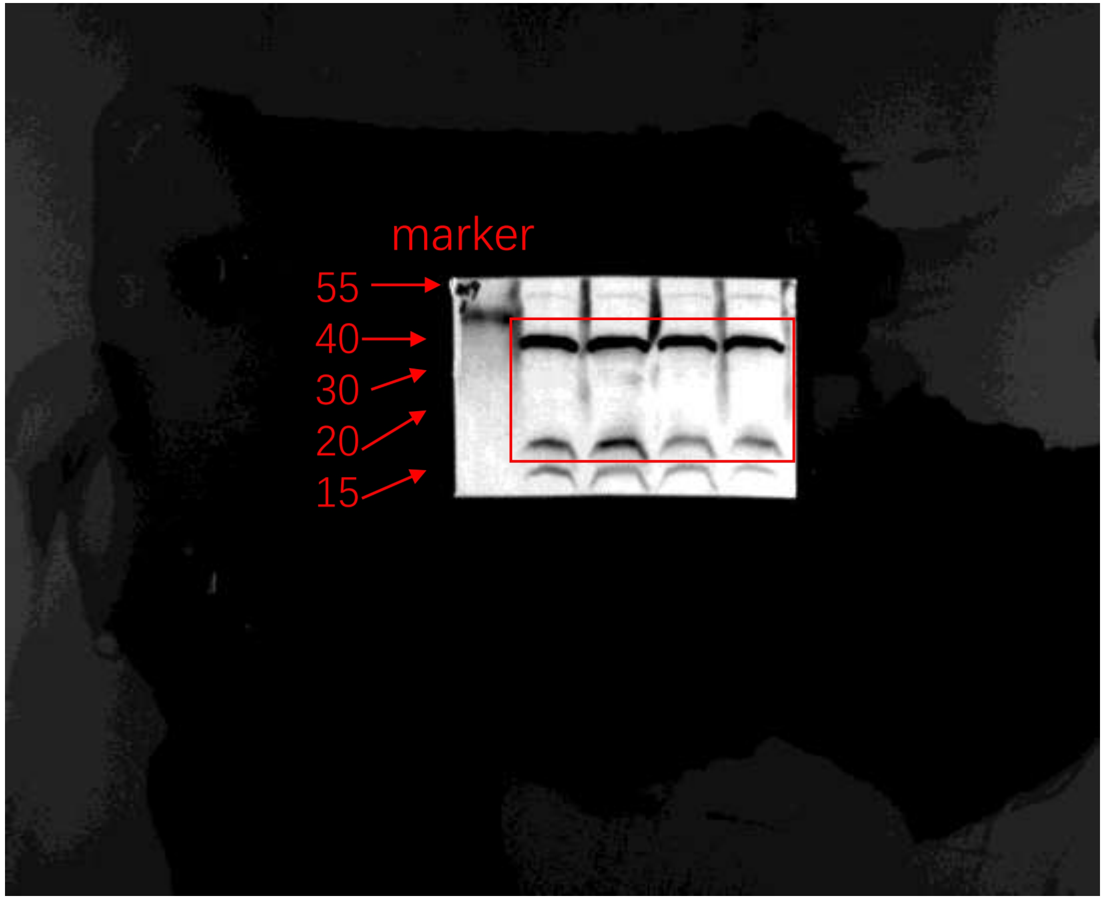

Caspase-1/Cleaved caspase-1

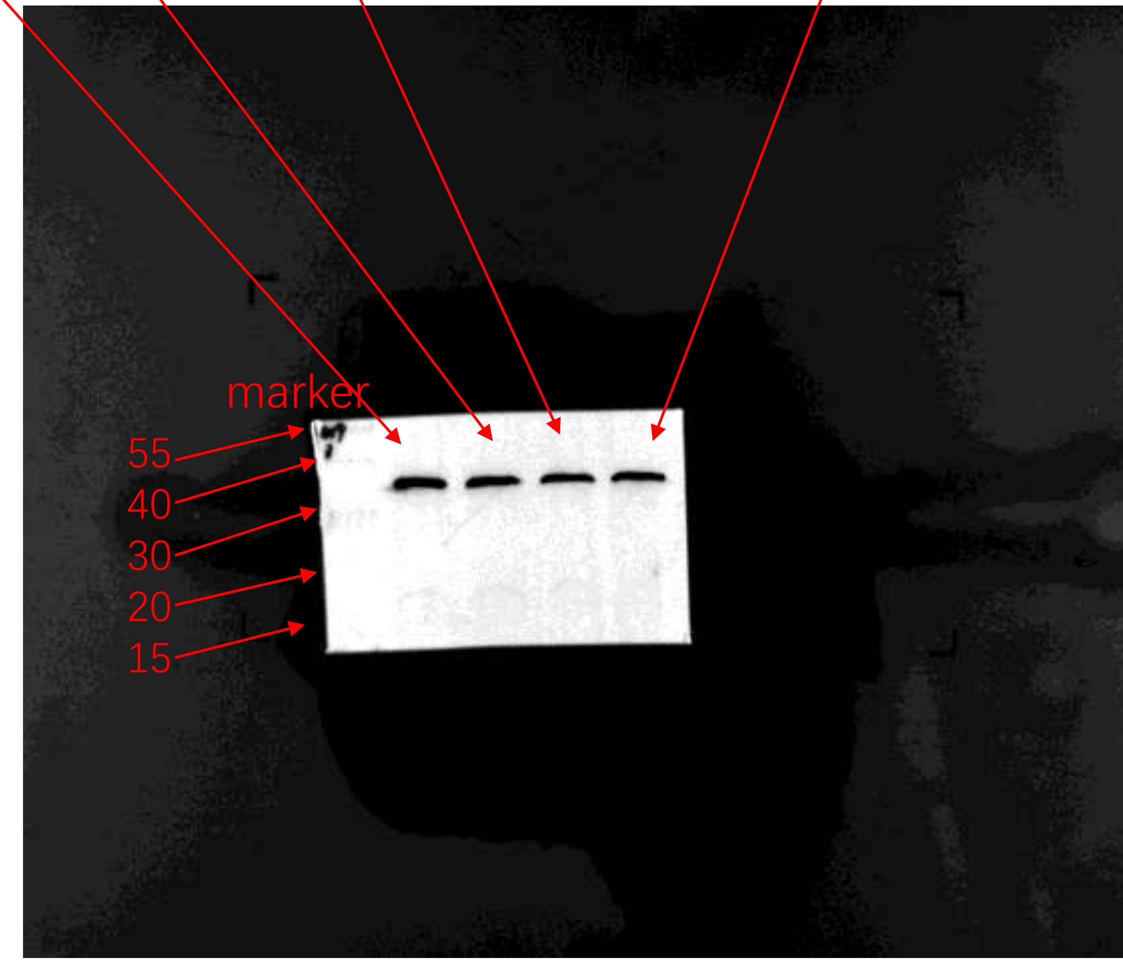

GAPDH

Caspase-1/Cleaved caspase-1:48/20kDa
2023-09-04-No.4

Control, LPS, LPS+mimics-NC,LPS+miRNA-206-3p mimics

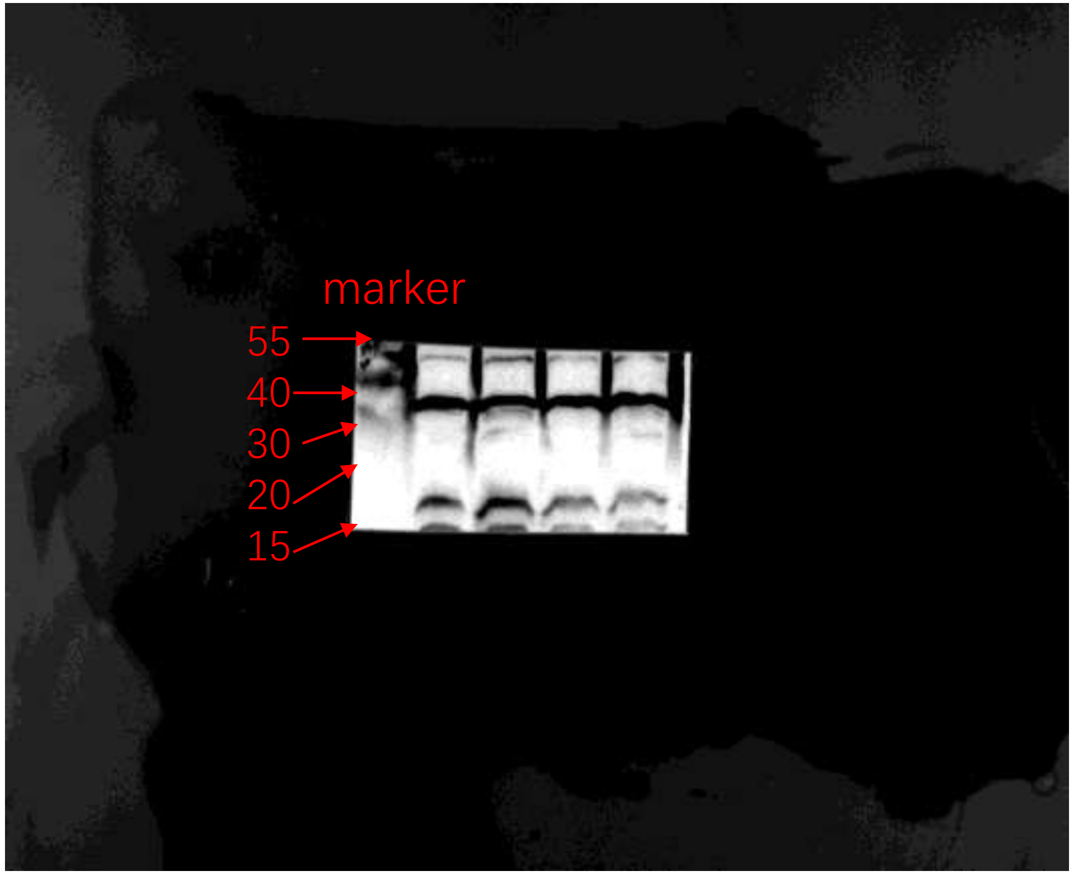

Caspase-1/Cleaved caspase-1

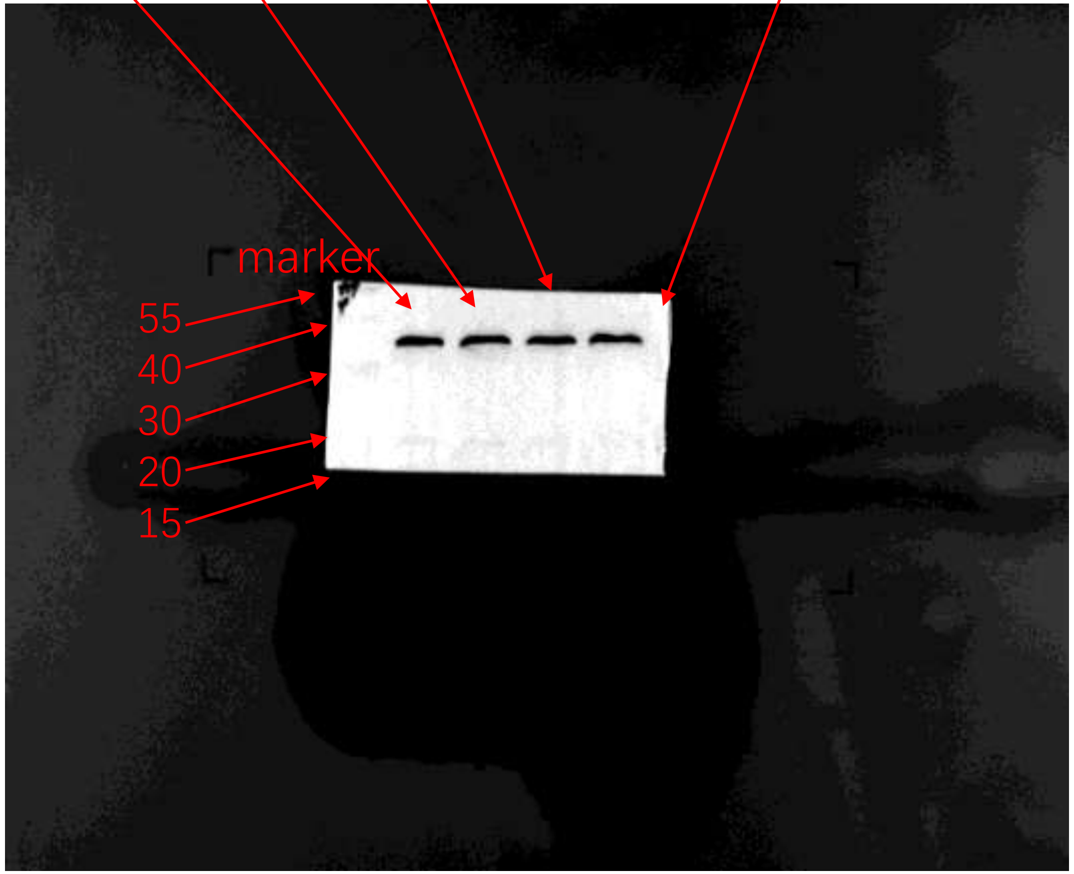

GAPDH

IL-1β-17kDa

2023-04-09

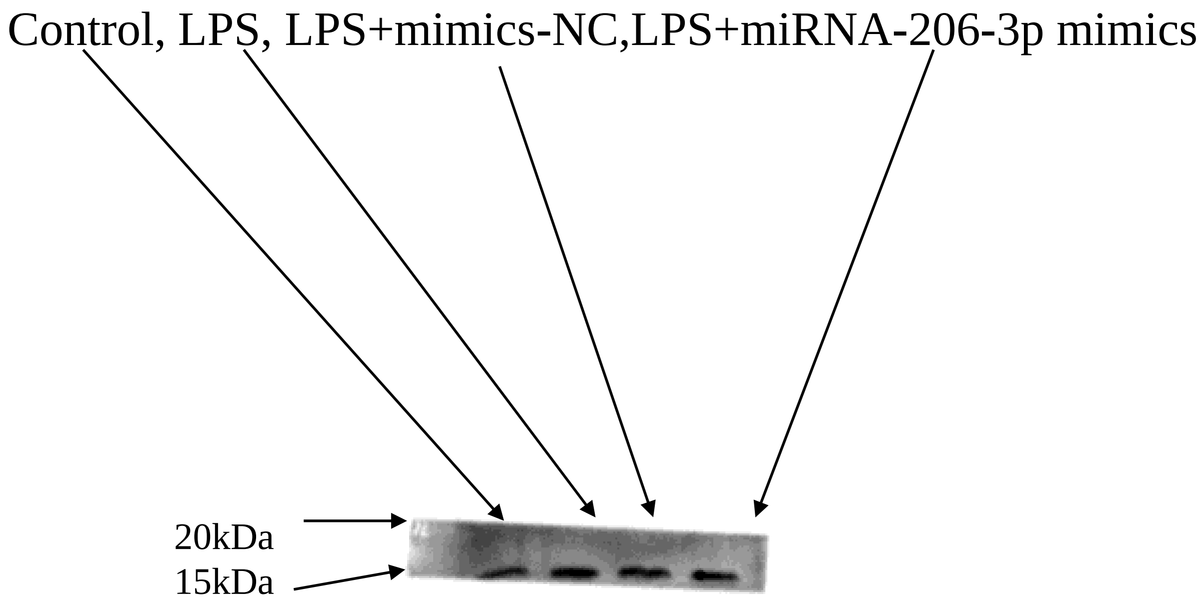

IL-1β

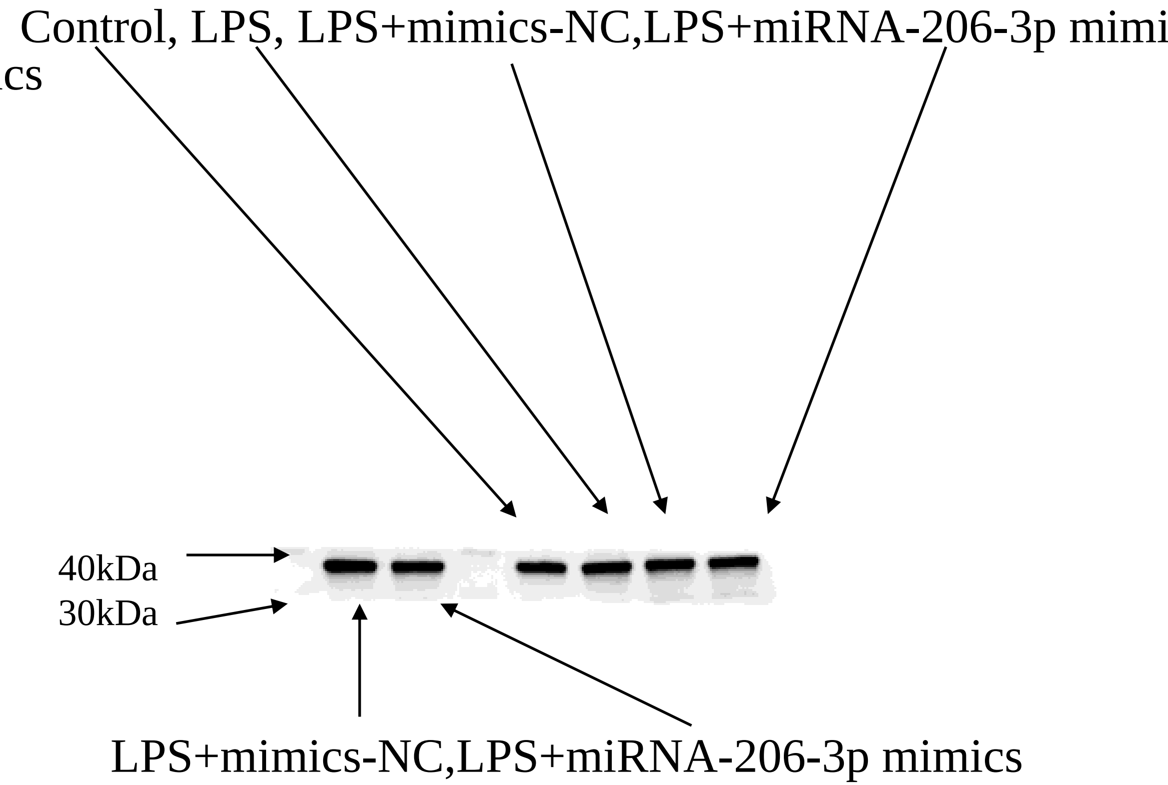

GAPDH

IL-1β-17kDa

2023-04-12

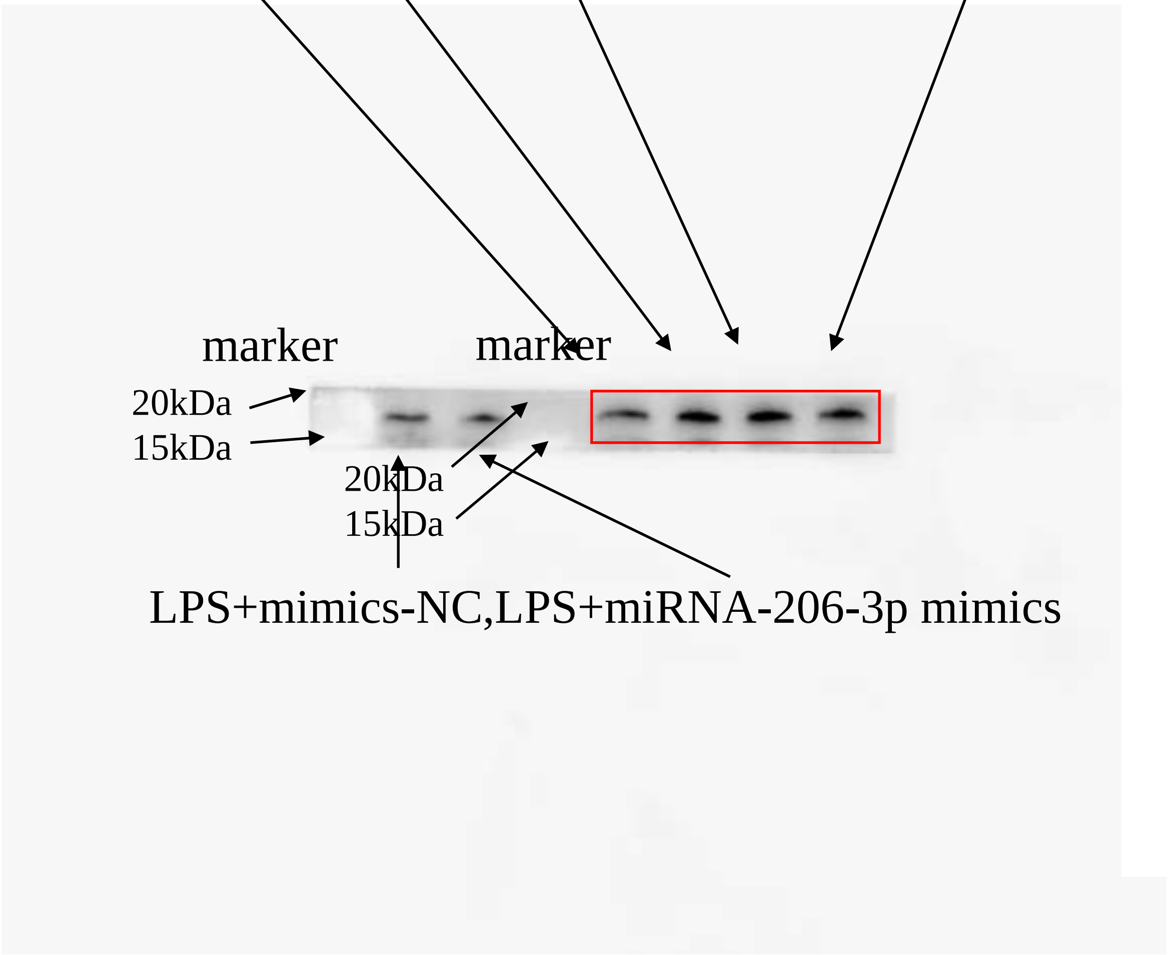

IL-1β

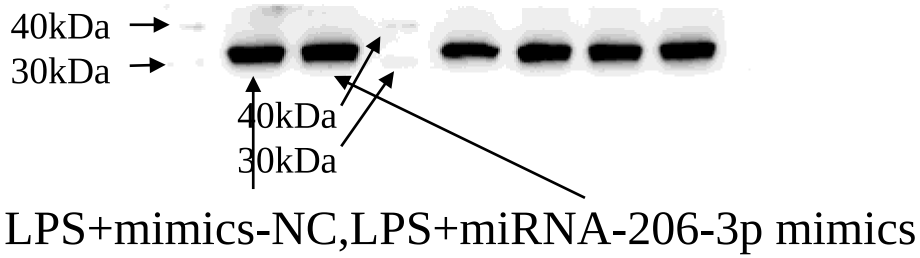

GAPDH

IL-1β-17kDa

2023-04-13

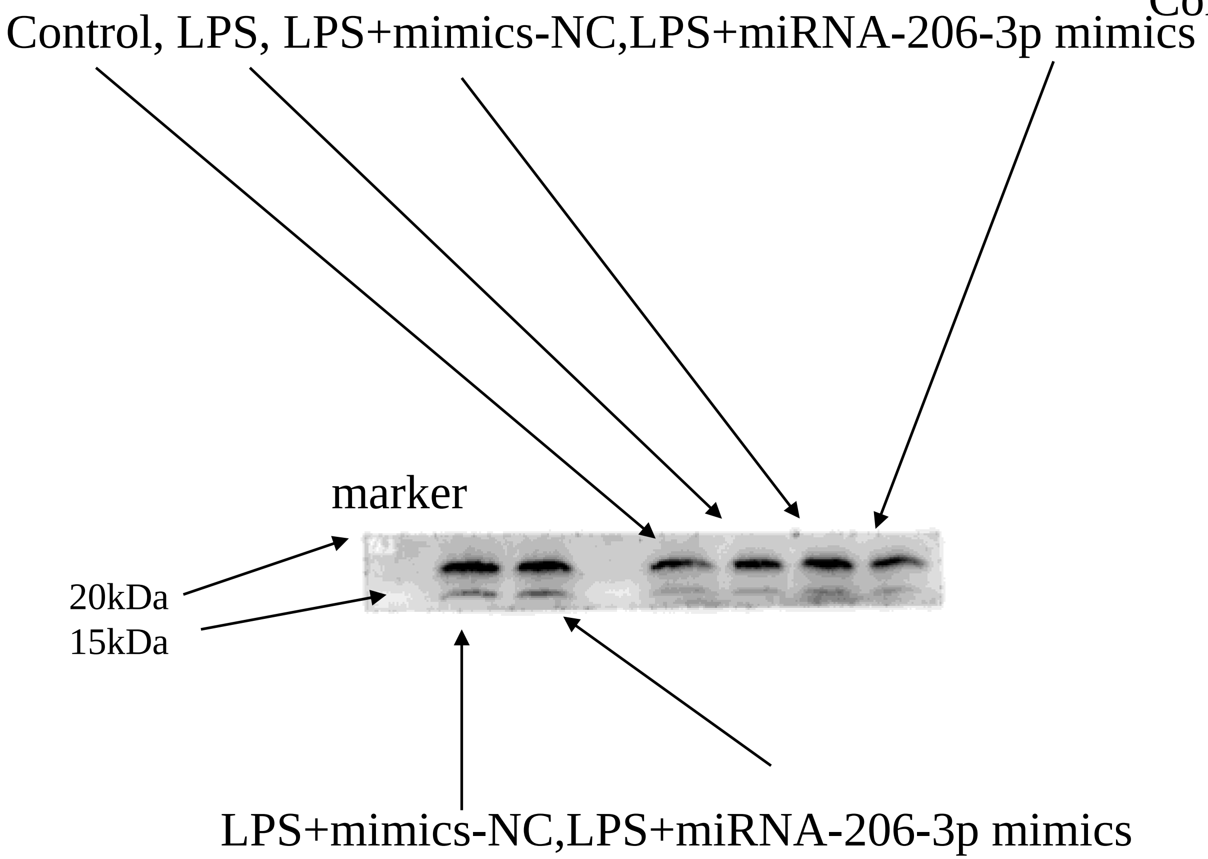

IL-1β

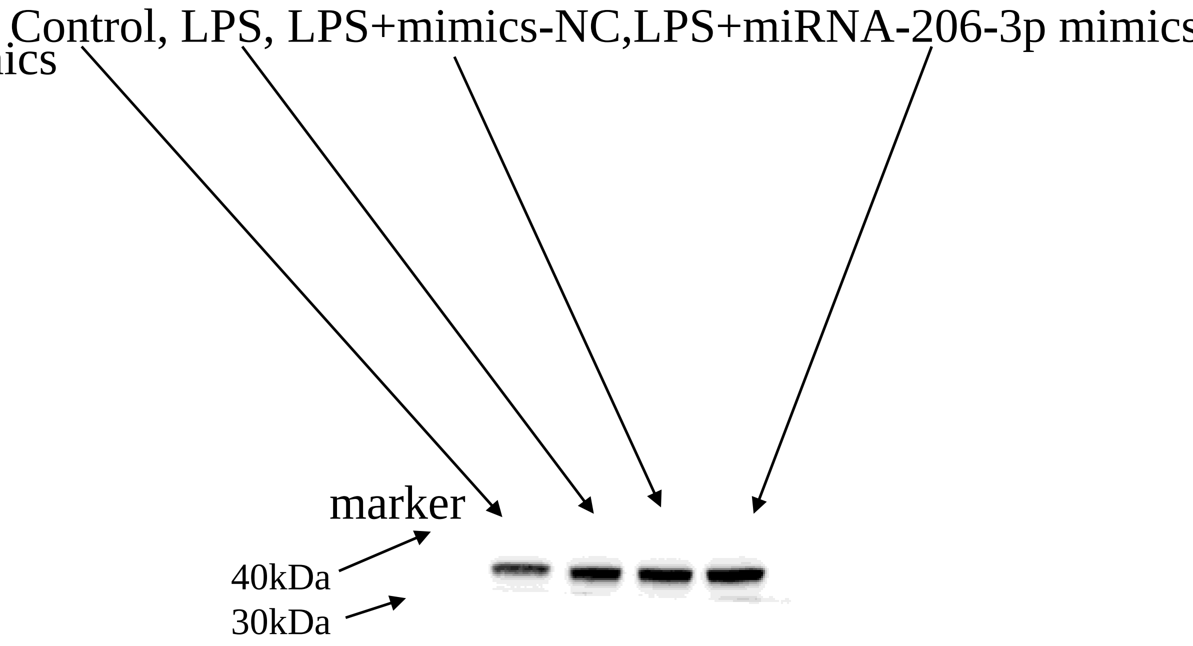

GAPDH

IL-18-26kDa
2023-05-07 No.1

Control, LPS, LPS+mimics-NC,LPS+miRNA-206-3p mimics

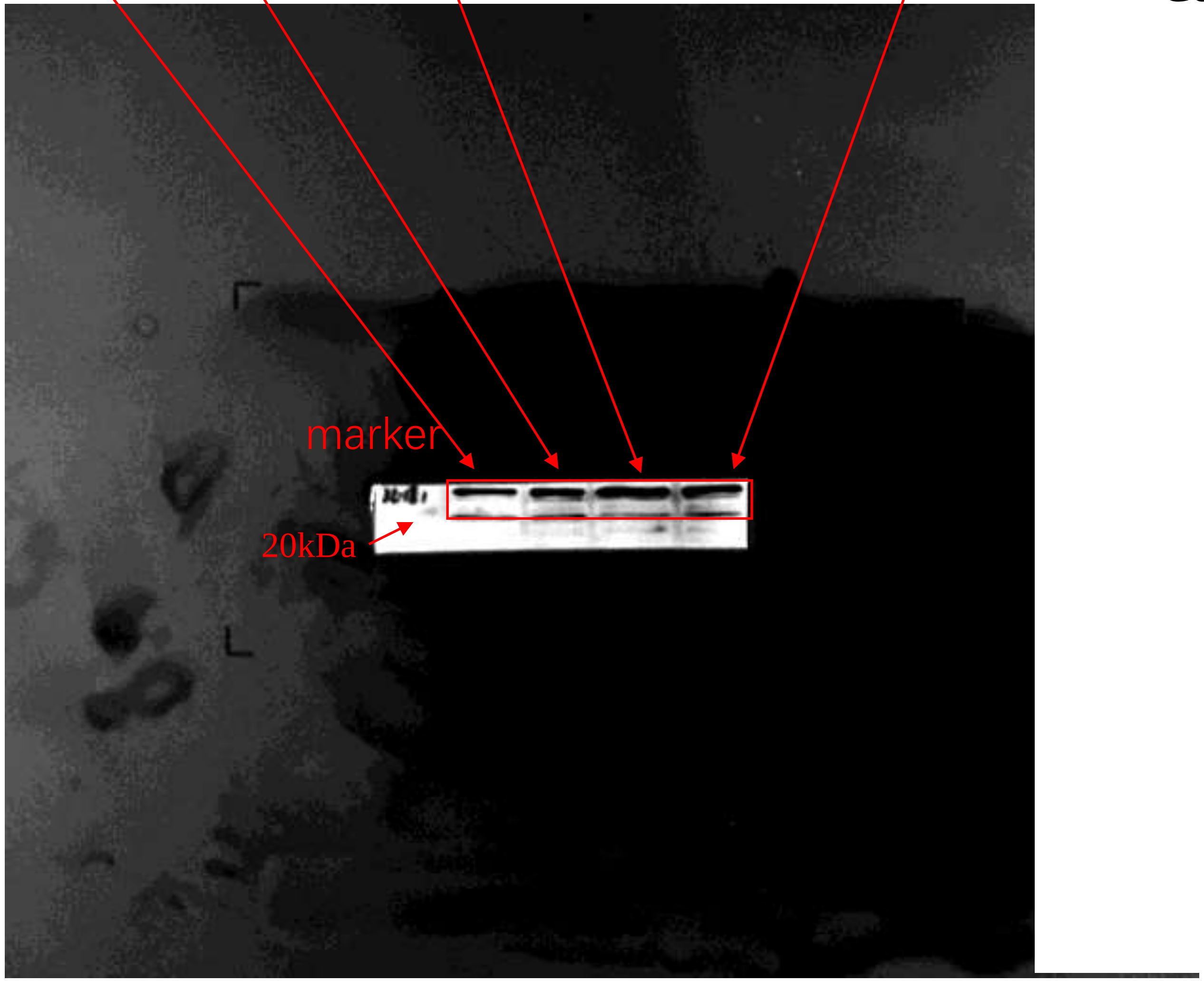

IL-18

Control, LPS, LPS+mimics-NC,LPS+miRNA-206-3p mim

marker

55kDa

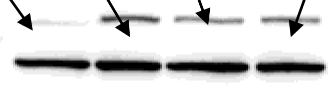

β-Actin

IL-18-26kDa

2023-05-18 No.3

Control, LPS, LPS+mimics-NC,LPS+miRNA-206-3p mimics

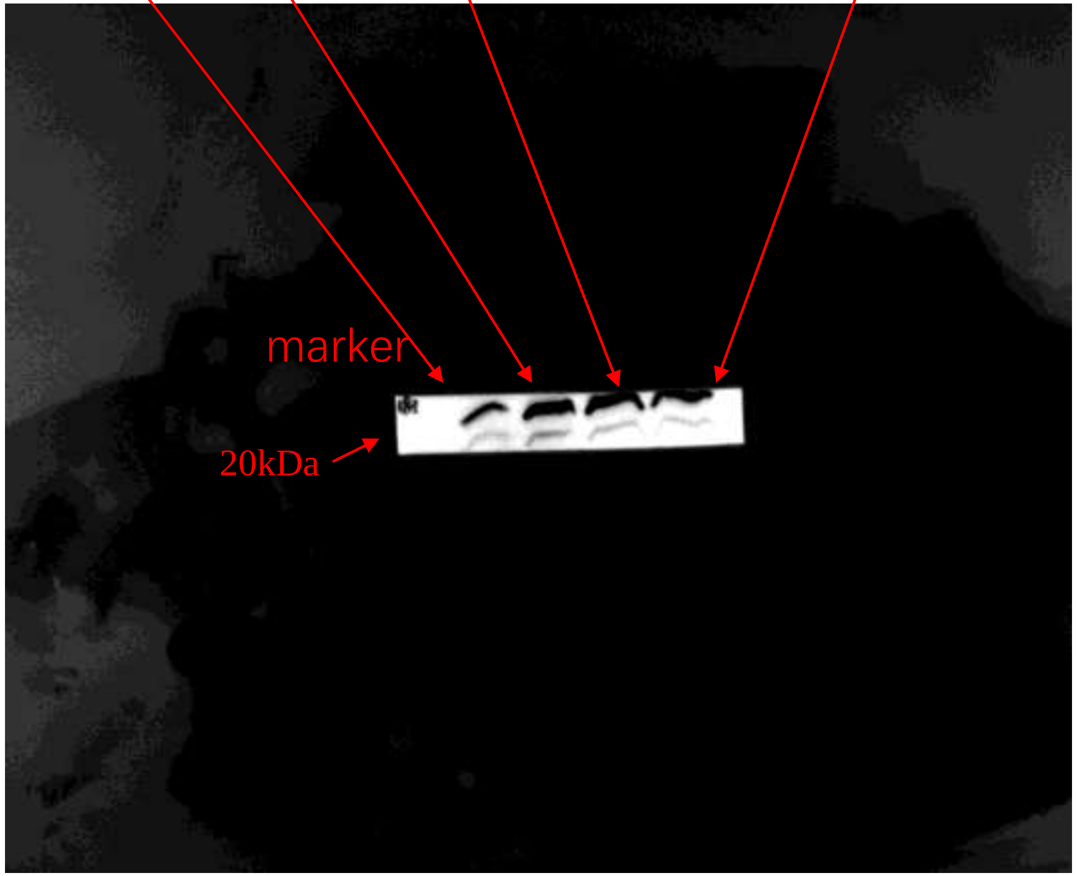

IL-18

Control, LPS, LPS+mimics-NC,LPS+miRNA-206-3p mim

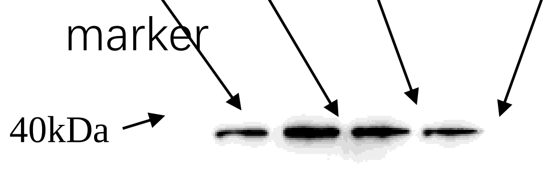

GAPDH

IL-18-26kDa

2023-05-31 No.1

Control, LPS, LPS+mimics-NC,LPS+miRNA-206-3p mimics

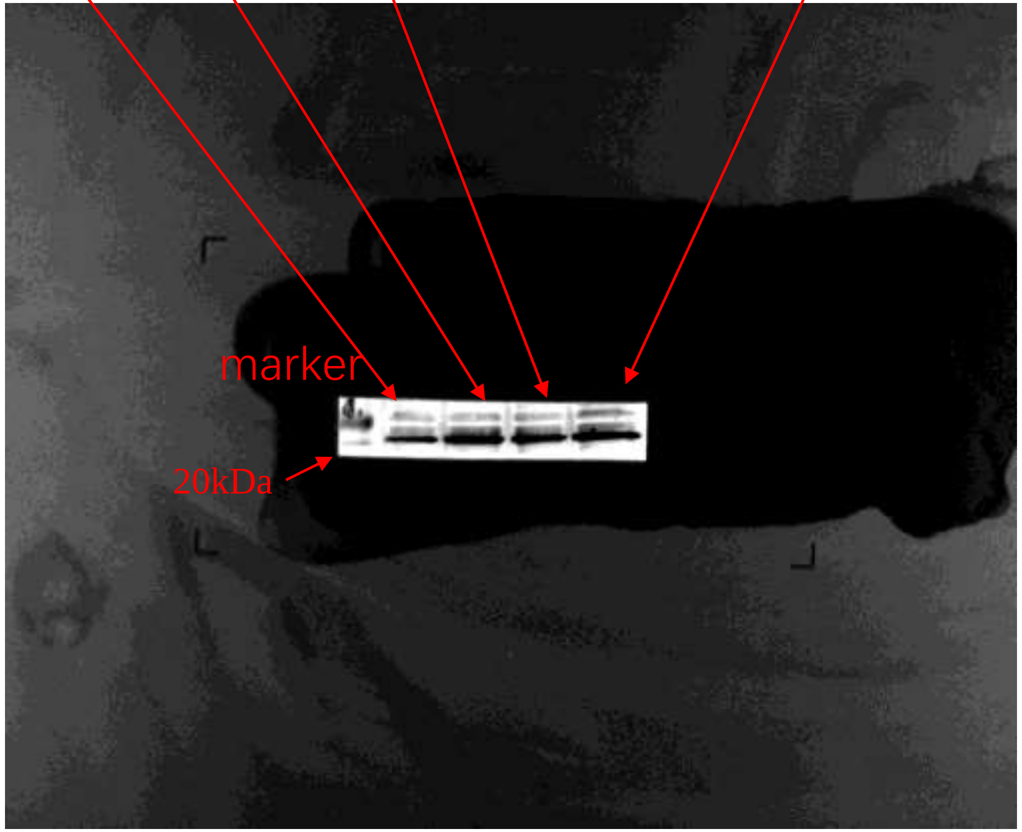

IL-18

Control, LPS, LPS+mimics-NC,LPS+miRNA-206-3p mim

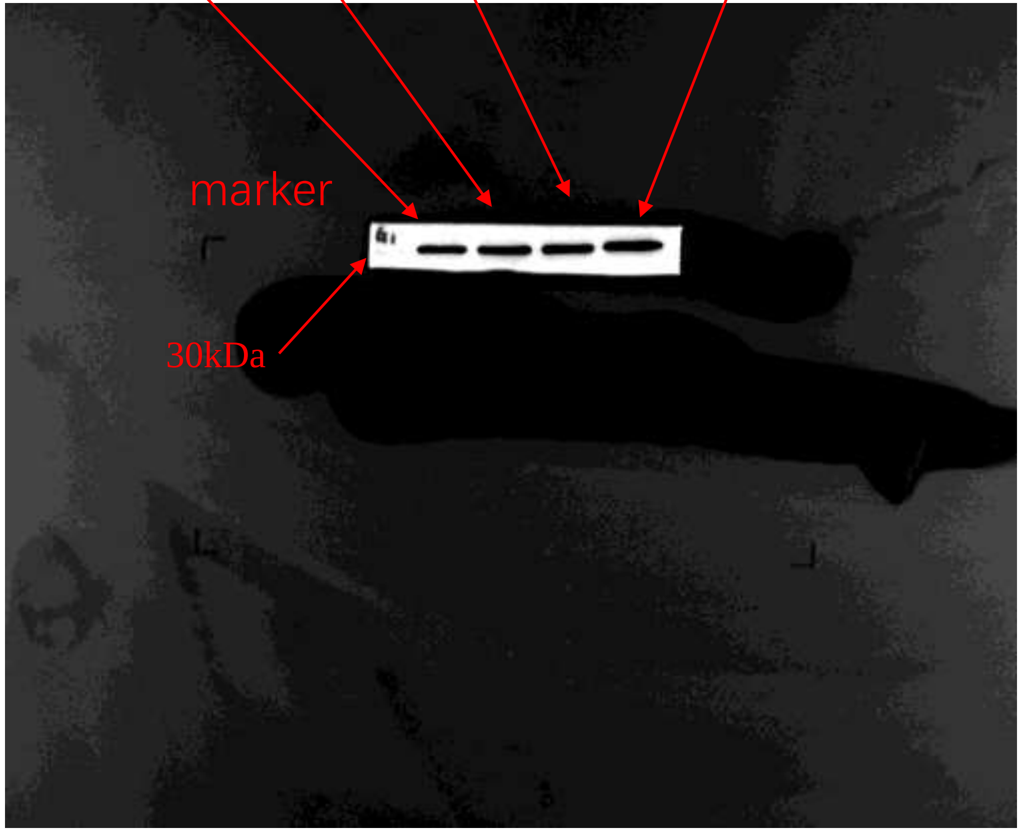

GAPDH

TLR4-96kDa

2023-04-06

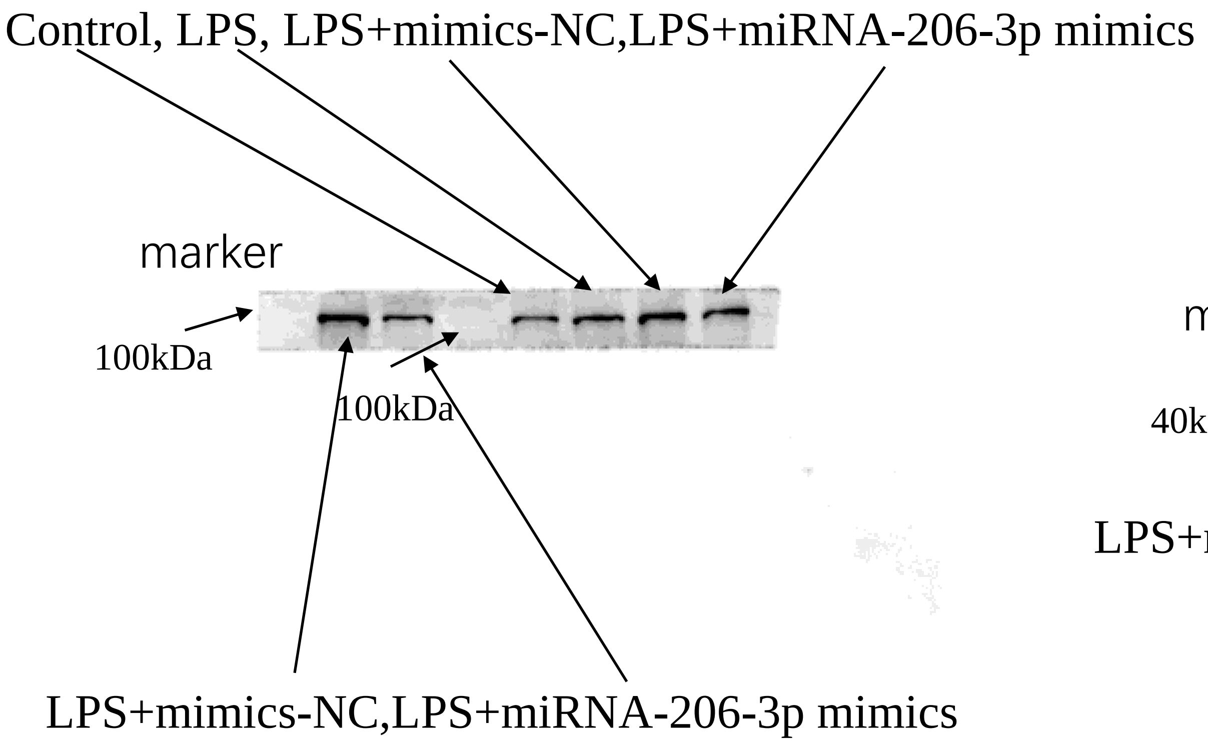

TLR4

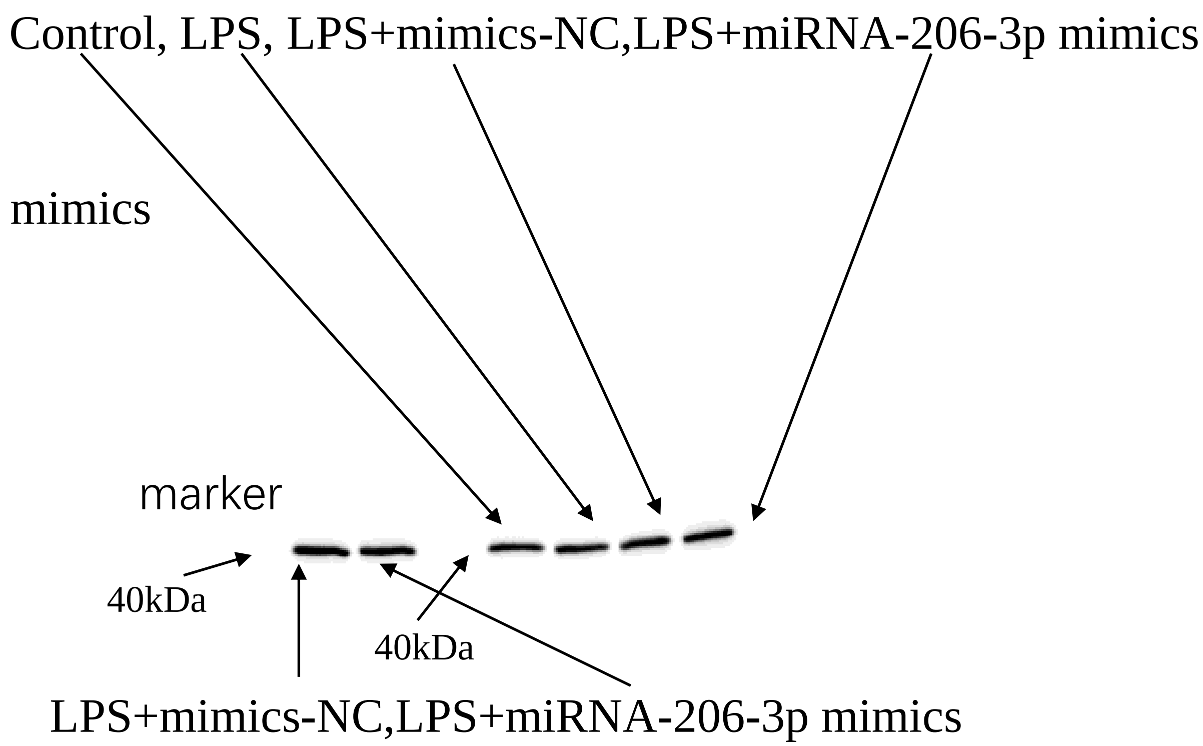

β-Actin

TLR4-96kDa

2023-04-07

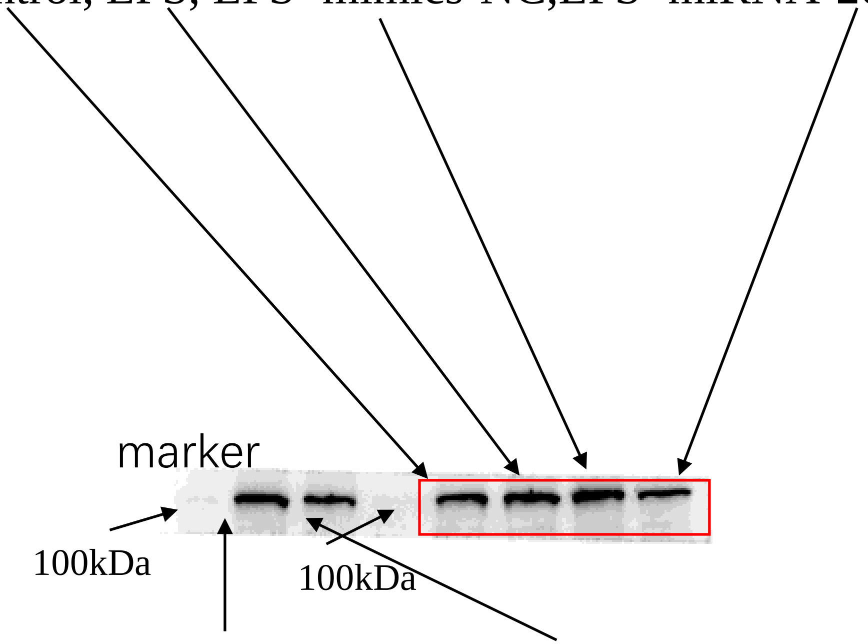

TLR4

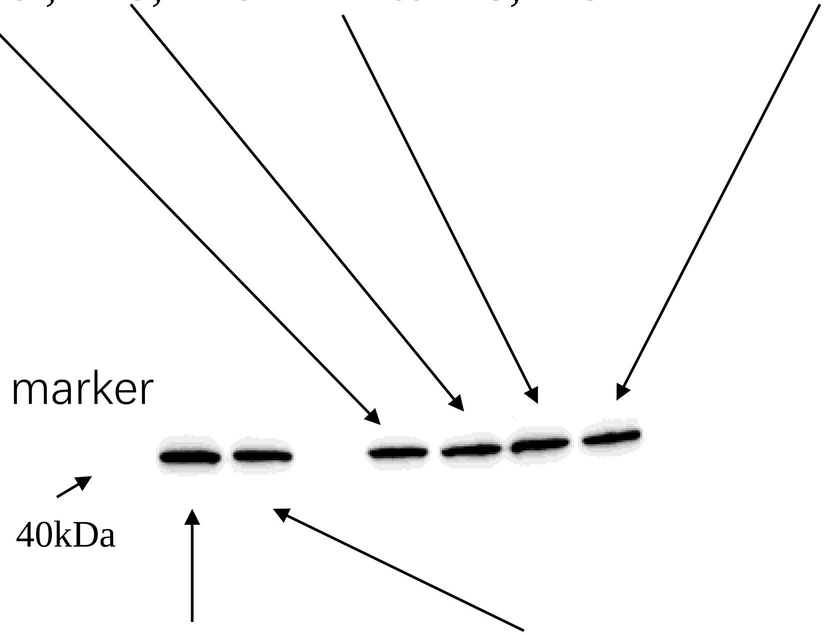

β-Actin

TLR4-96kDa

2023-04-08

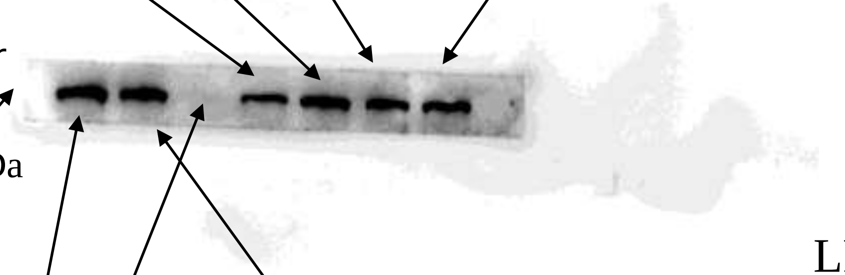

TLR4

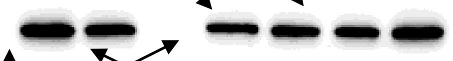

β-Actin

MyD88-33kDa

2023-04-06

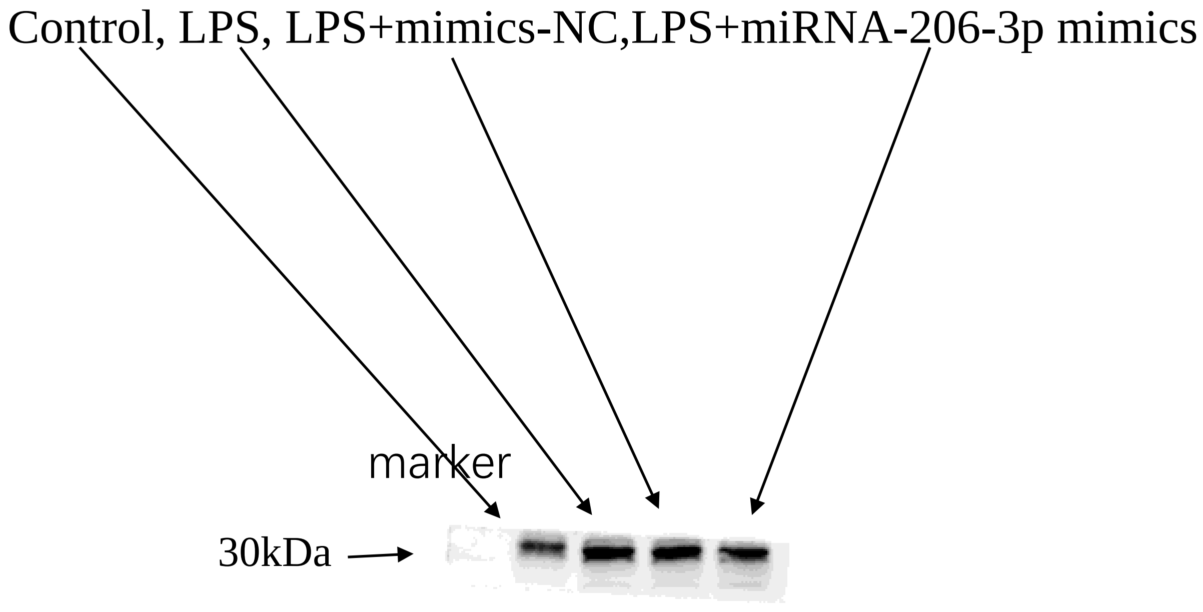

MyD88

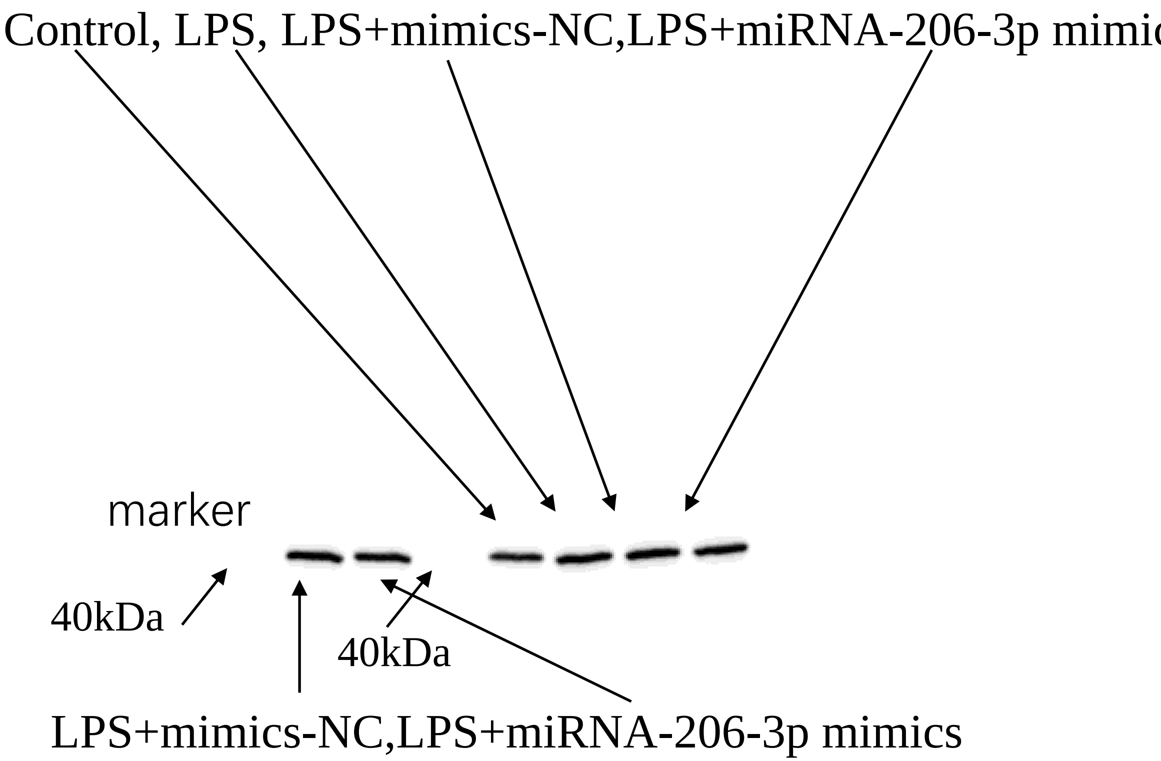

β-Actin

MyD88-33kDa
2023-04-07 No.1

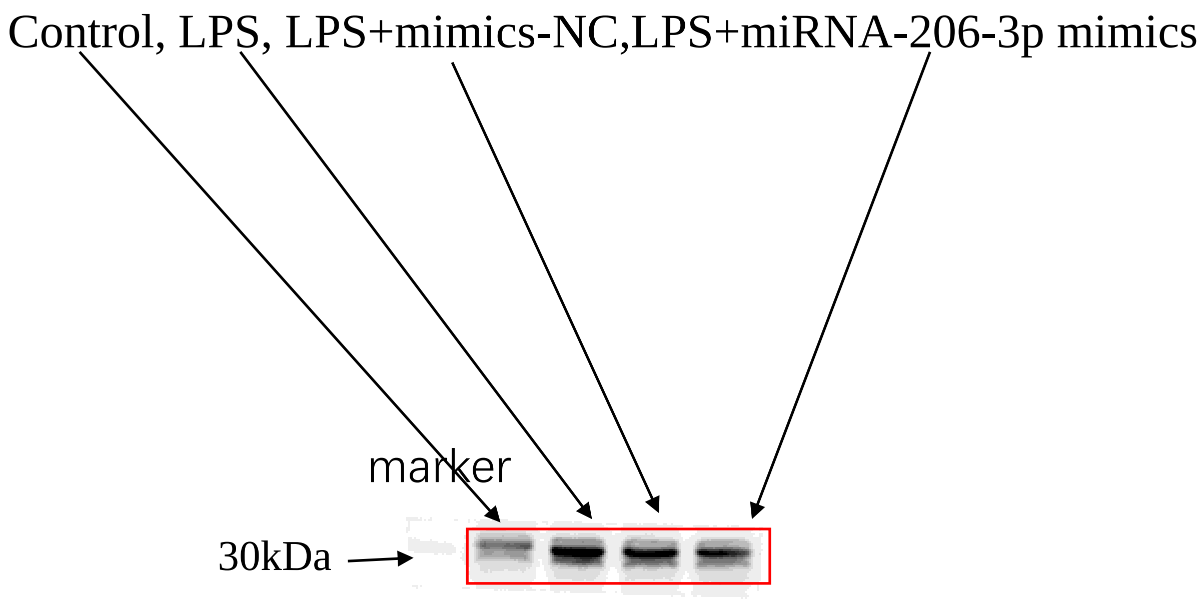

MyD88

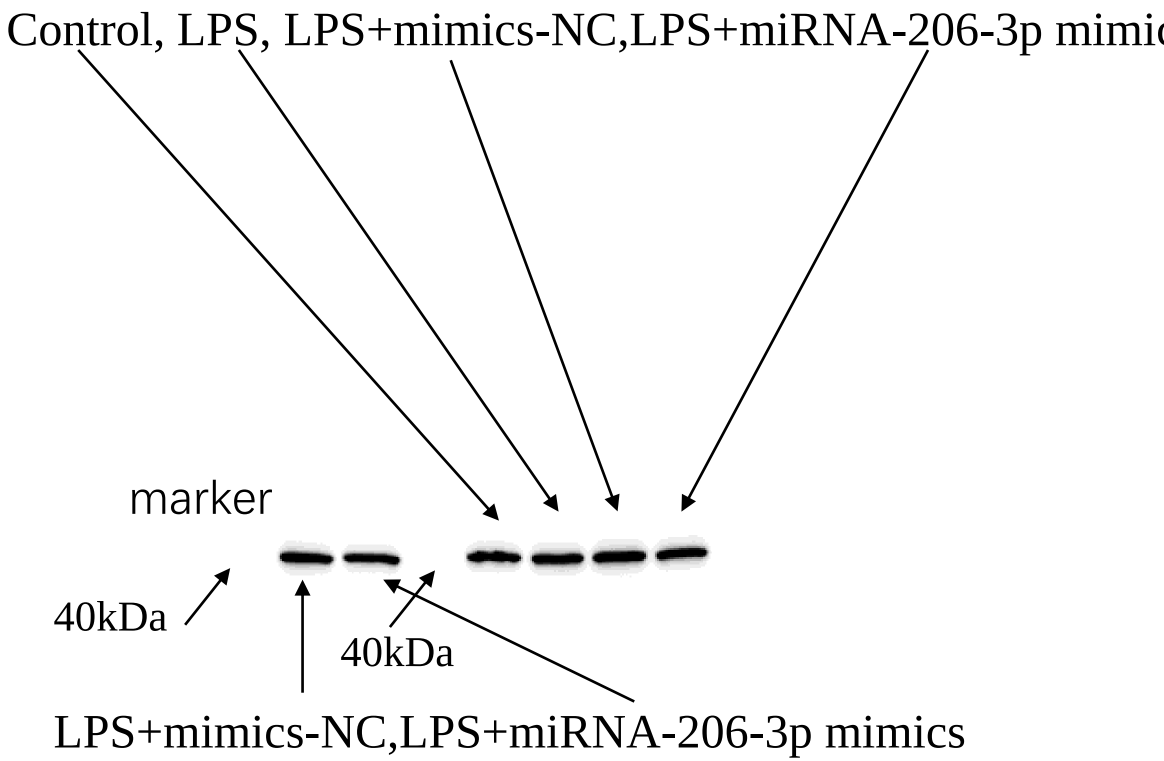

β-Actin

MyD88-33kDa

2023-04-07 No.2

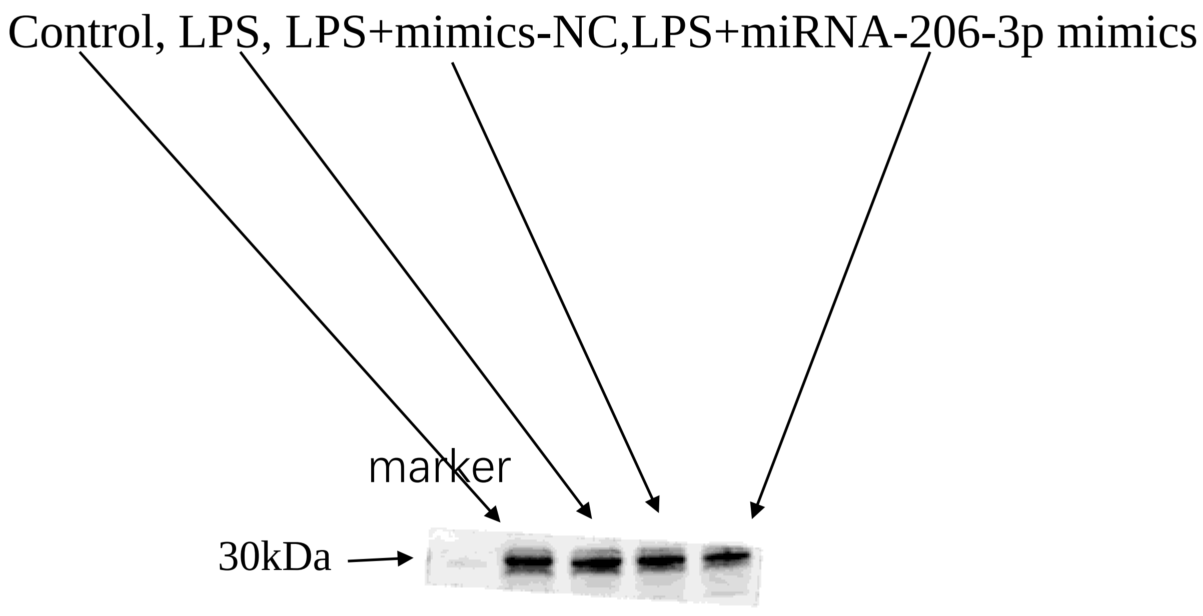

MyD88

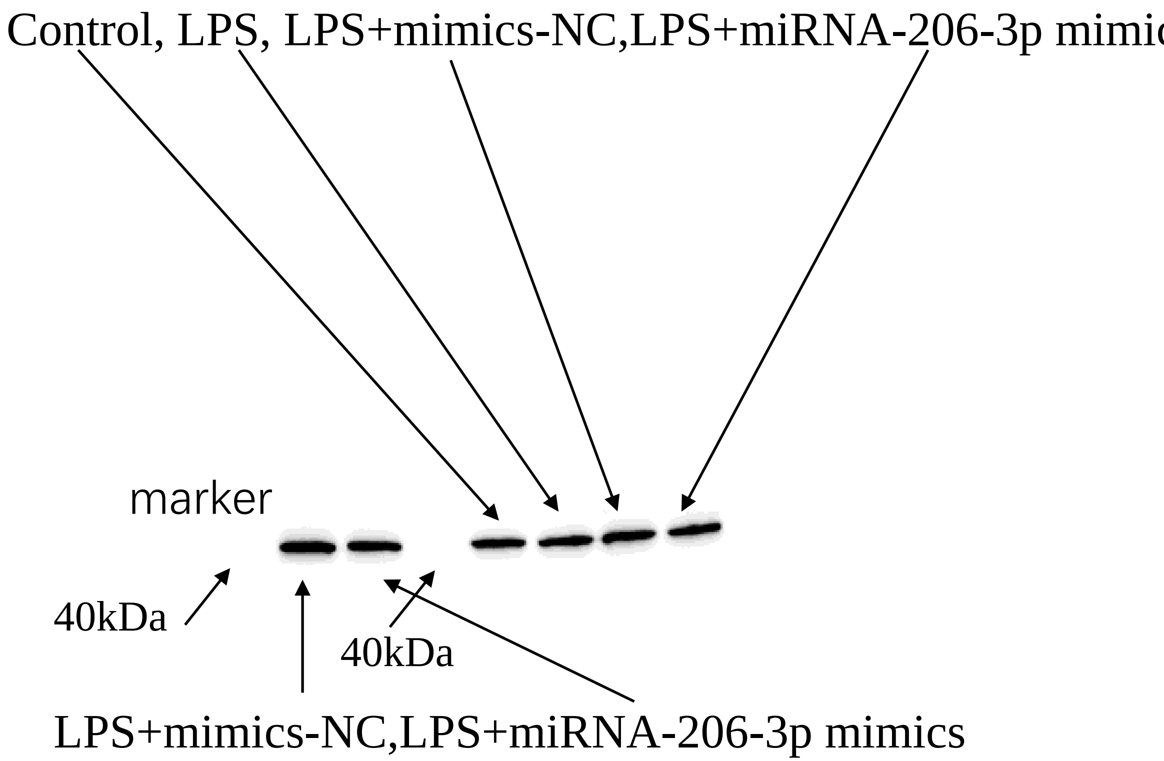

β-Actin

NF-κB-65kDa

2023-04-06

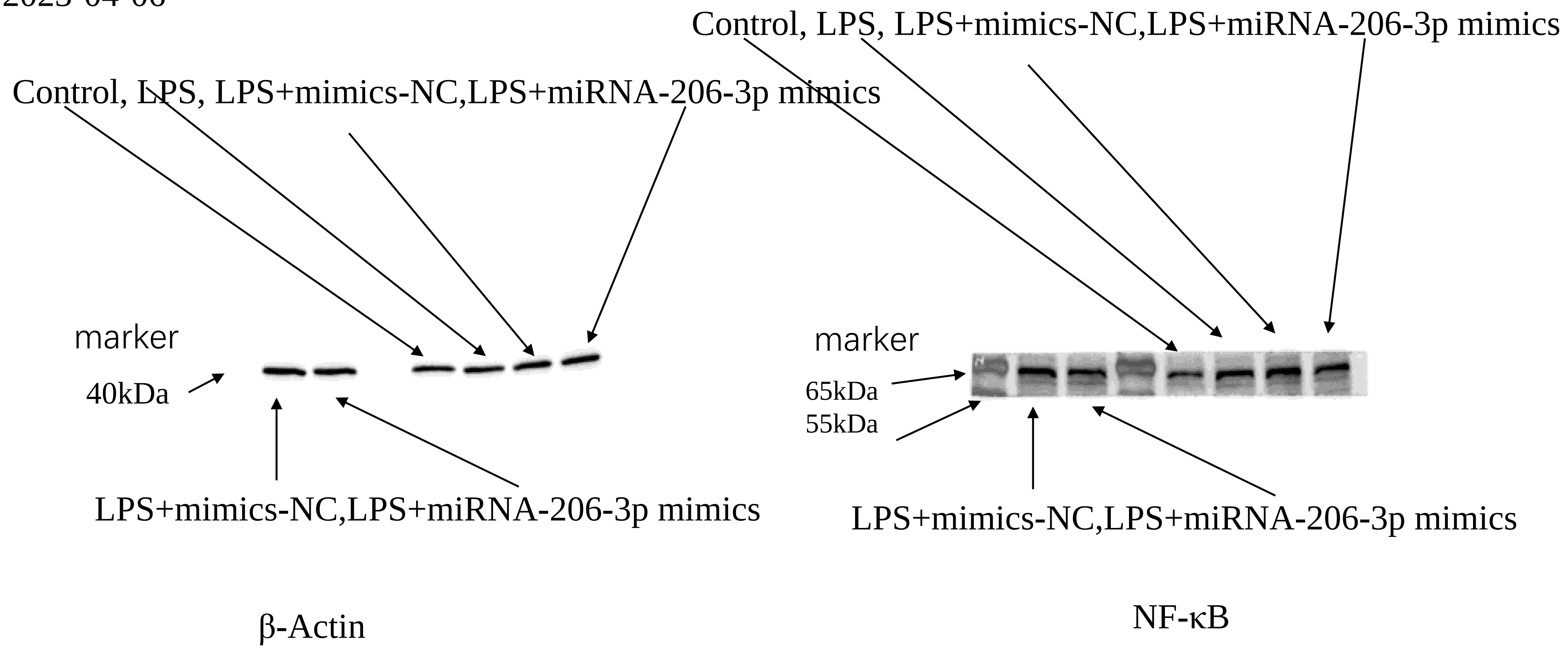

NF-κB-65kDa

2023-04-07

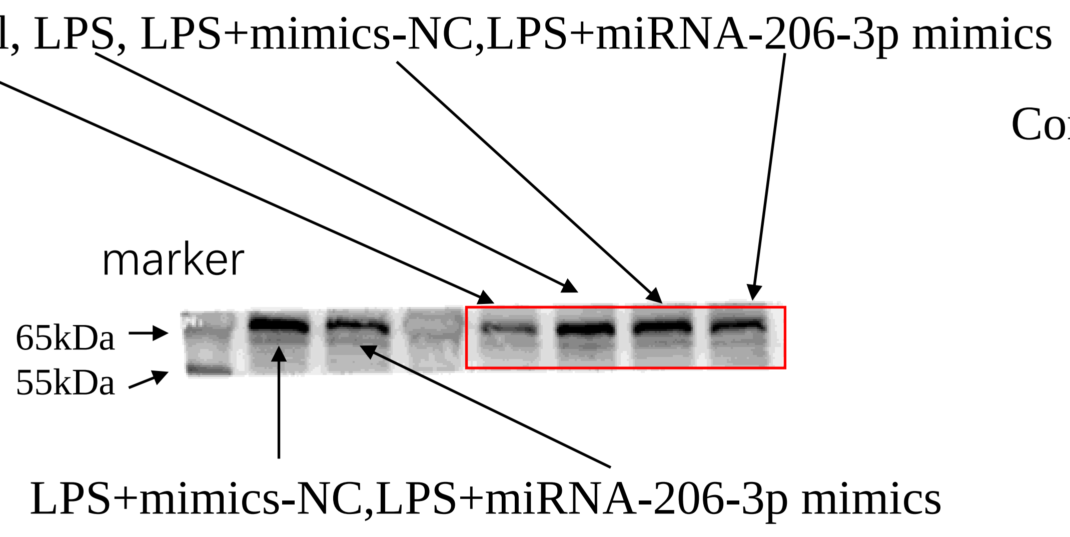

NF-κB

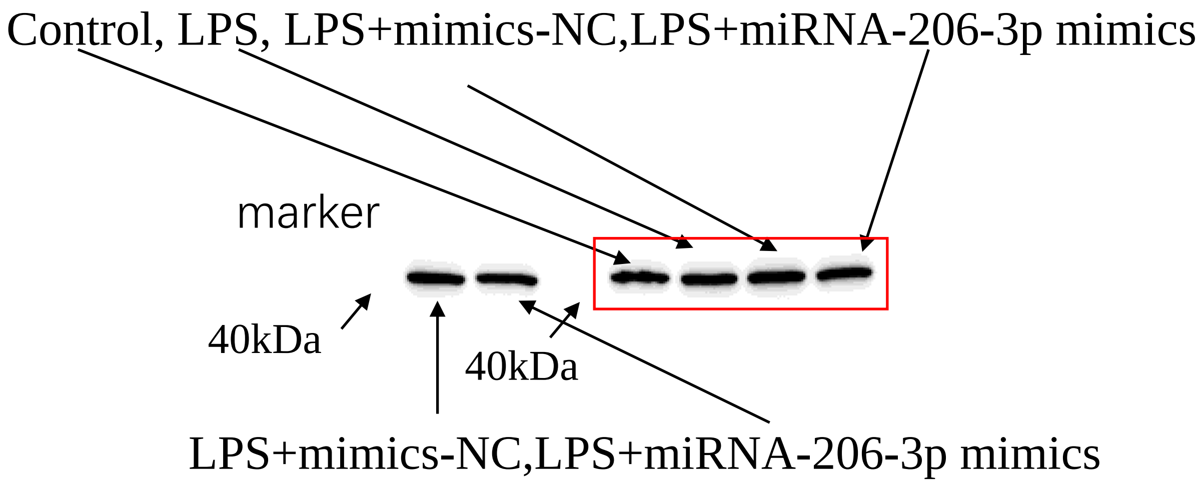

β-Actin

NF-κB-65kDa

2023-04-08

Control, LPS, LPS+mimics-NC,LPS+miRNA-206-3p mimics

marker

40kDa

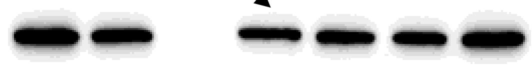

LPS+mimics-NC,LPS+miRNA-206-3p mimics

β-Actin

Control, LPS, LPS+mimics-NC,LPS+miRNA-206-3p mimics

marker

65kDa

55kDa

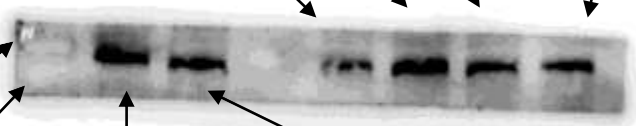

LPS+mimics-NC,LPS+miRNA-206-3p mimics

NF-κB

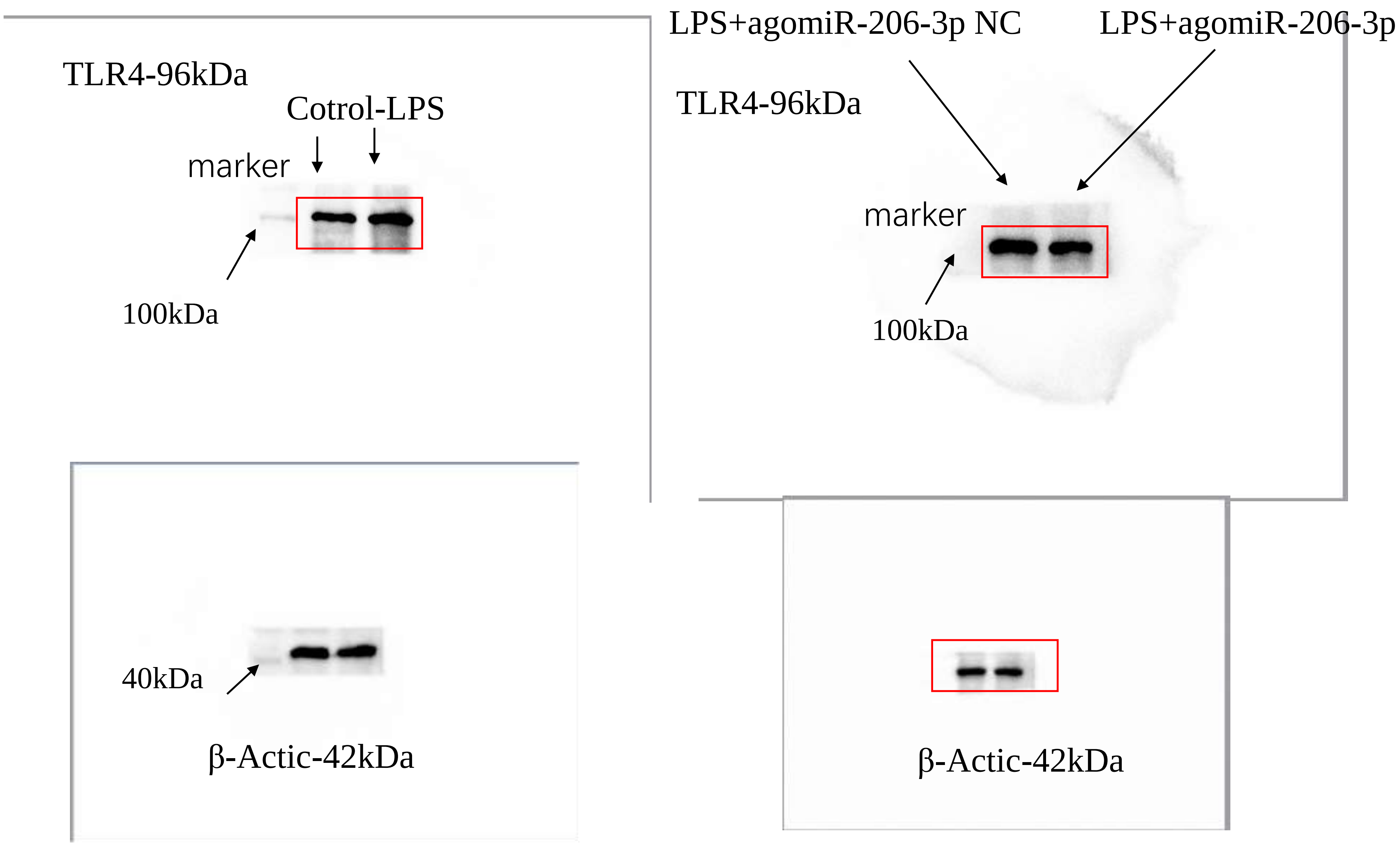

2022-12-15

2022-12-25

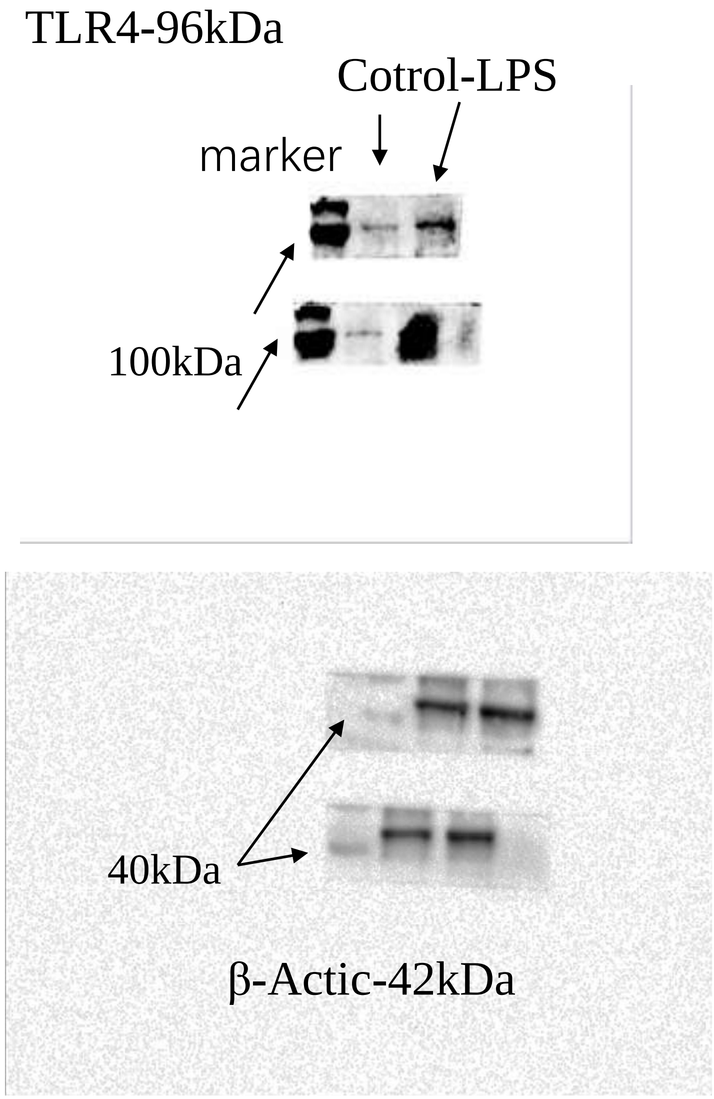

2022-08-25

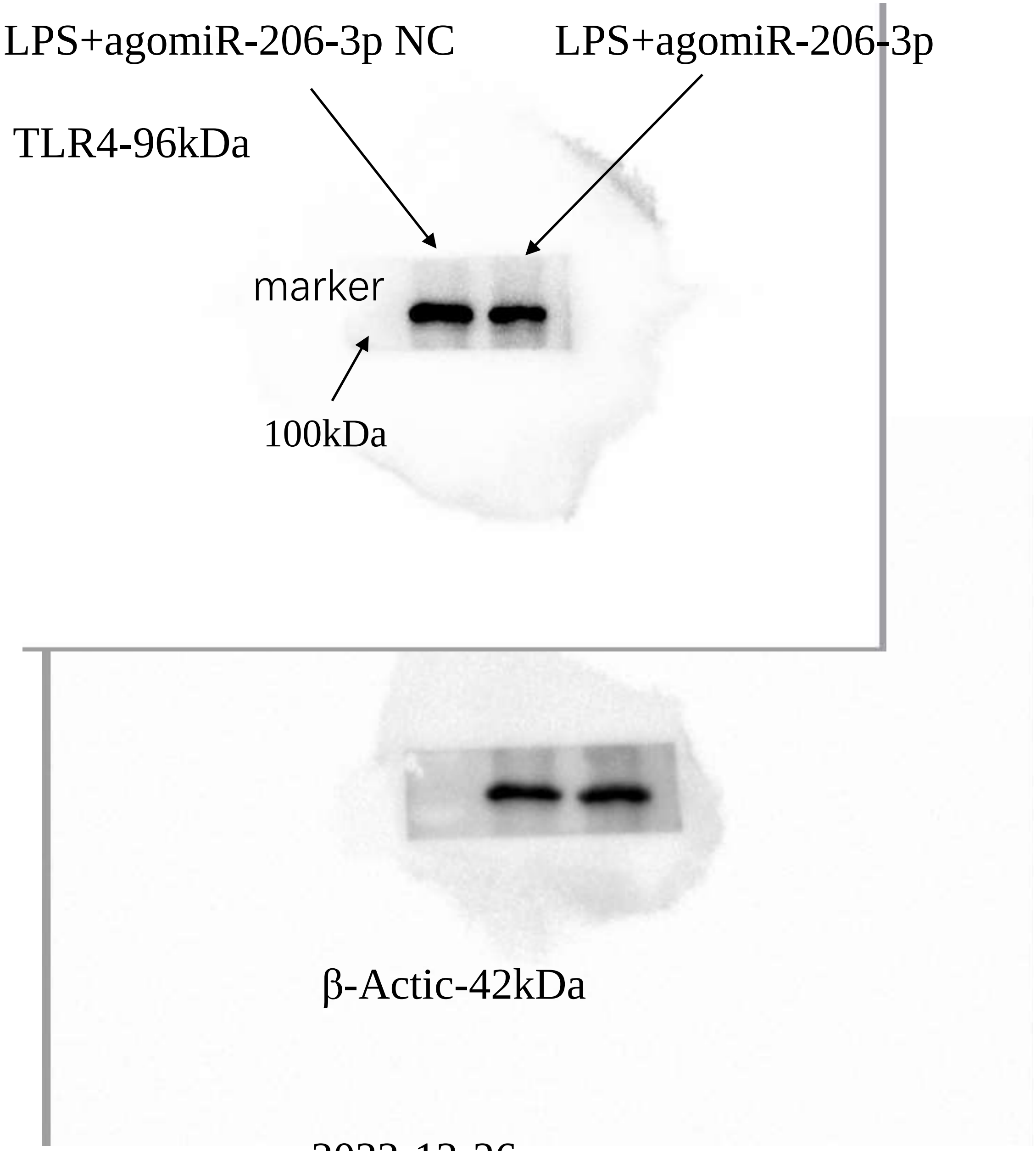

2022-12-26

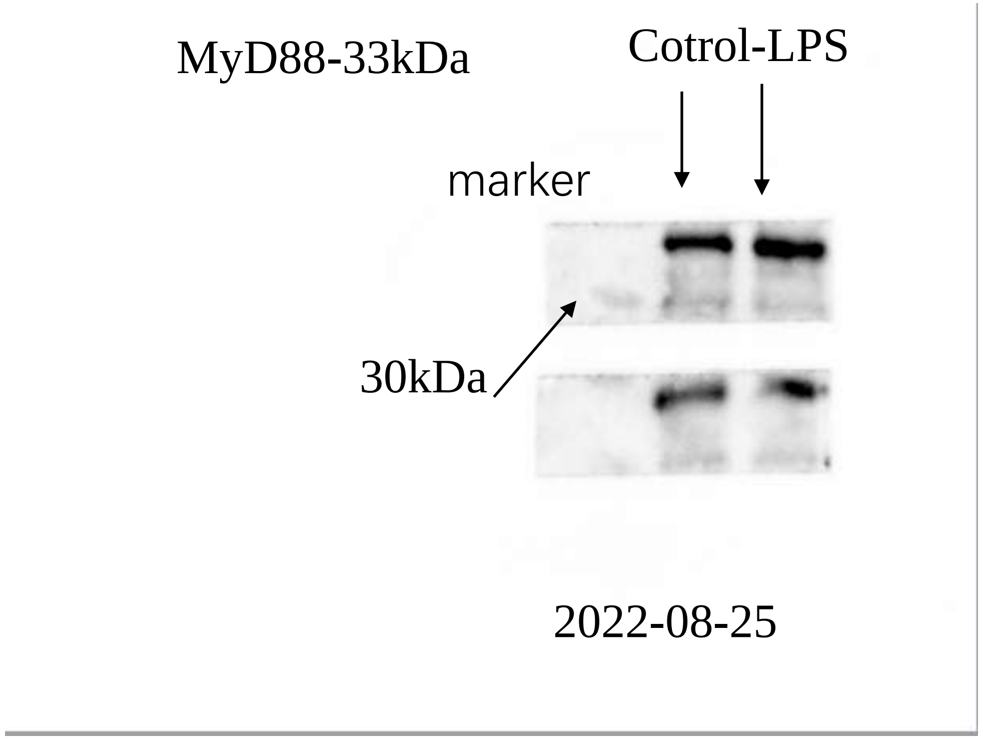
MyD88-33kDa
Cotrol-LPS
marker
30kDa
2022-08-25

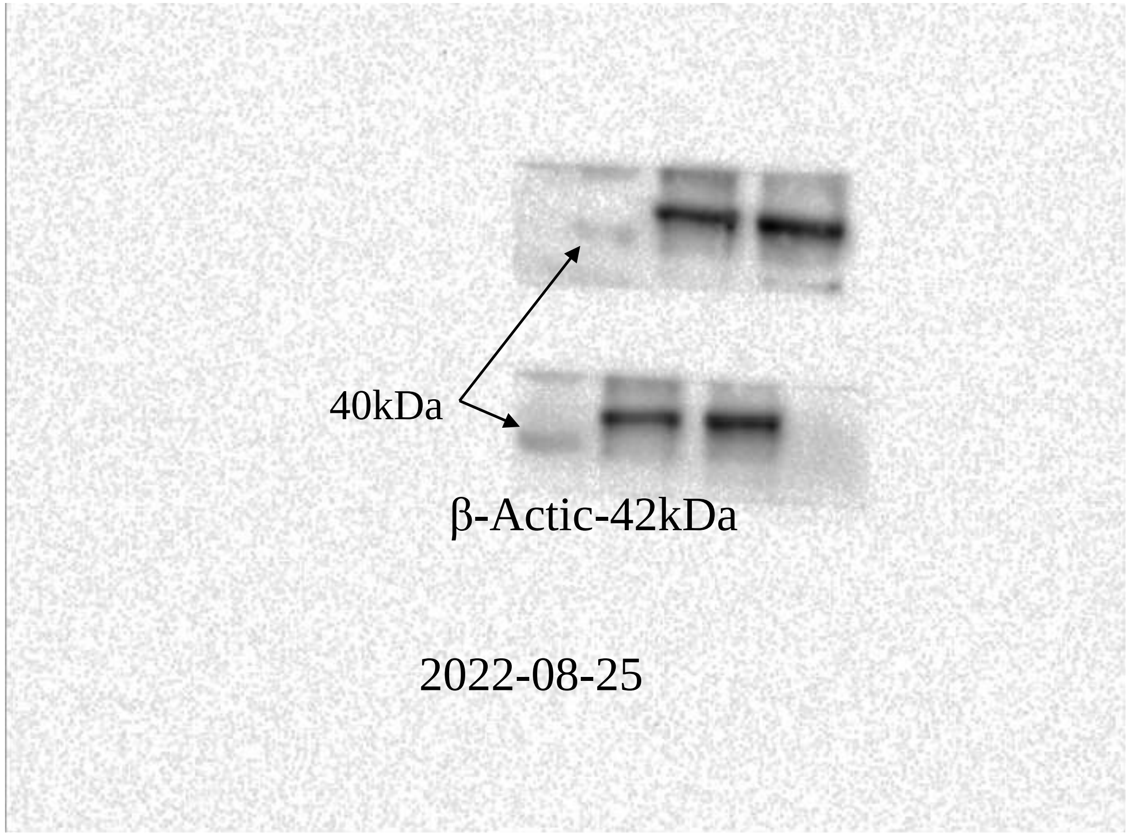
40kDa
β-Actic-42kDa
2022-08-25

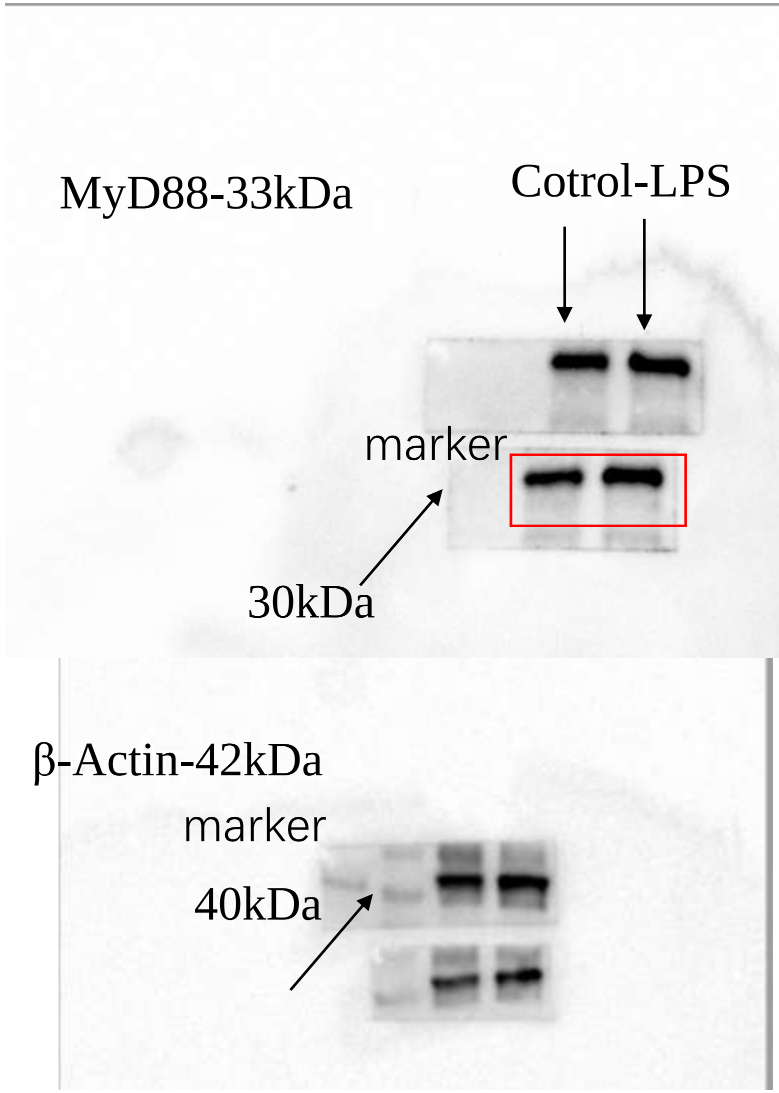

2022-08-24

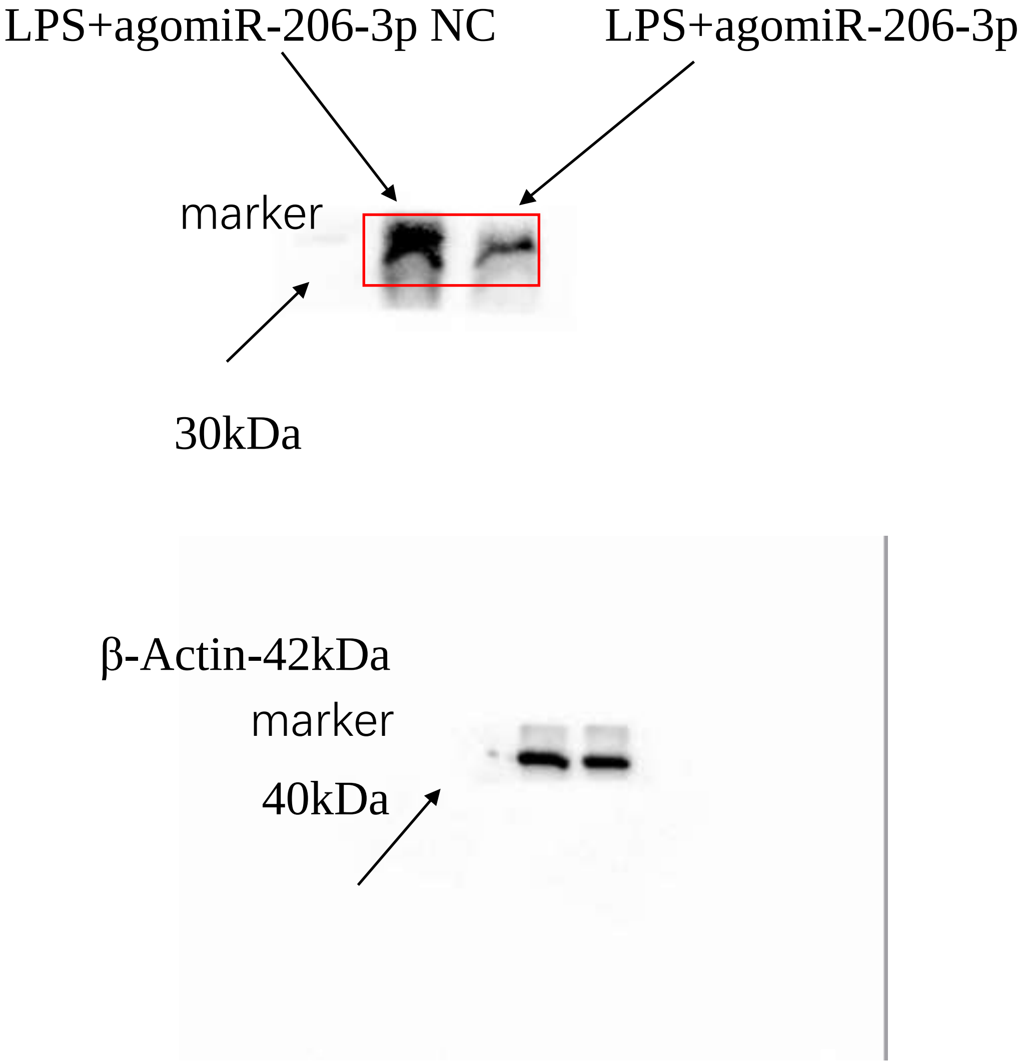

2023-01-05

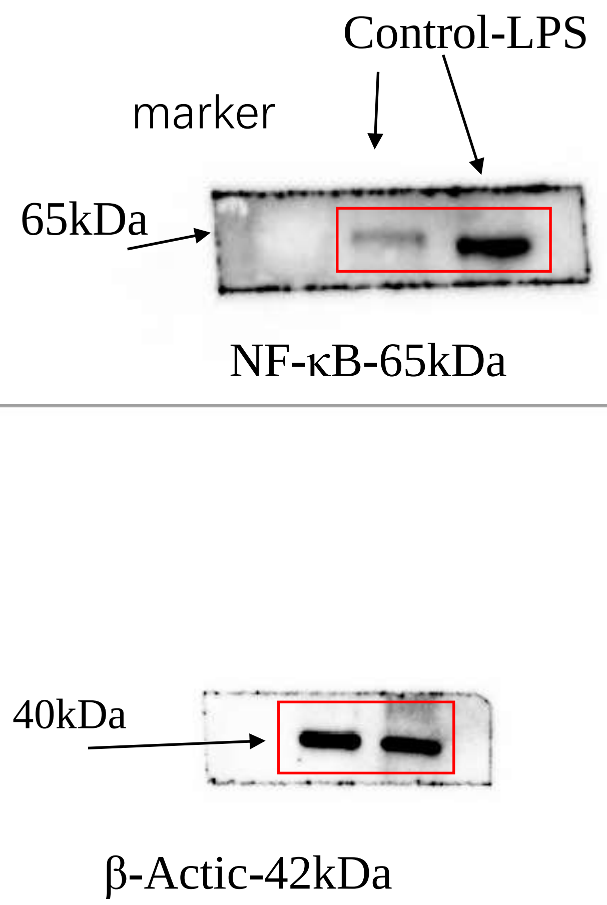

2022-09-25

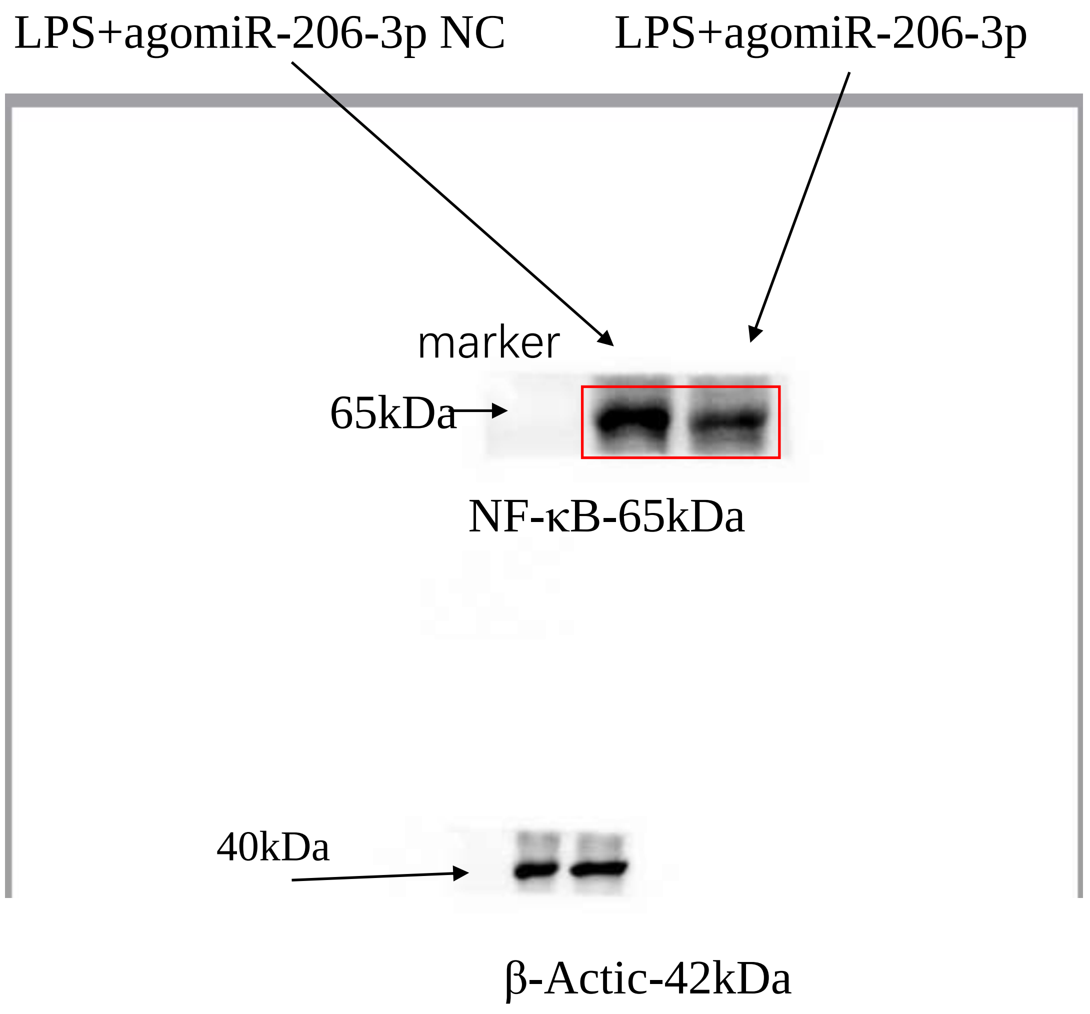

2022-12-27No.3

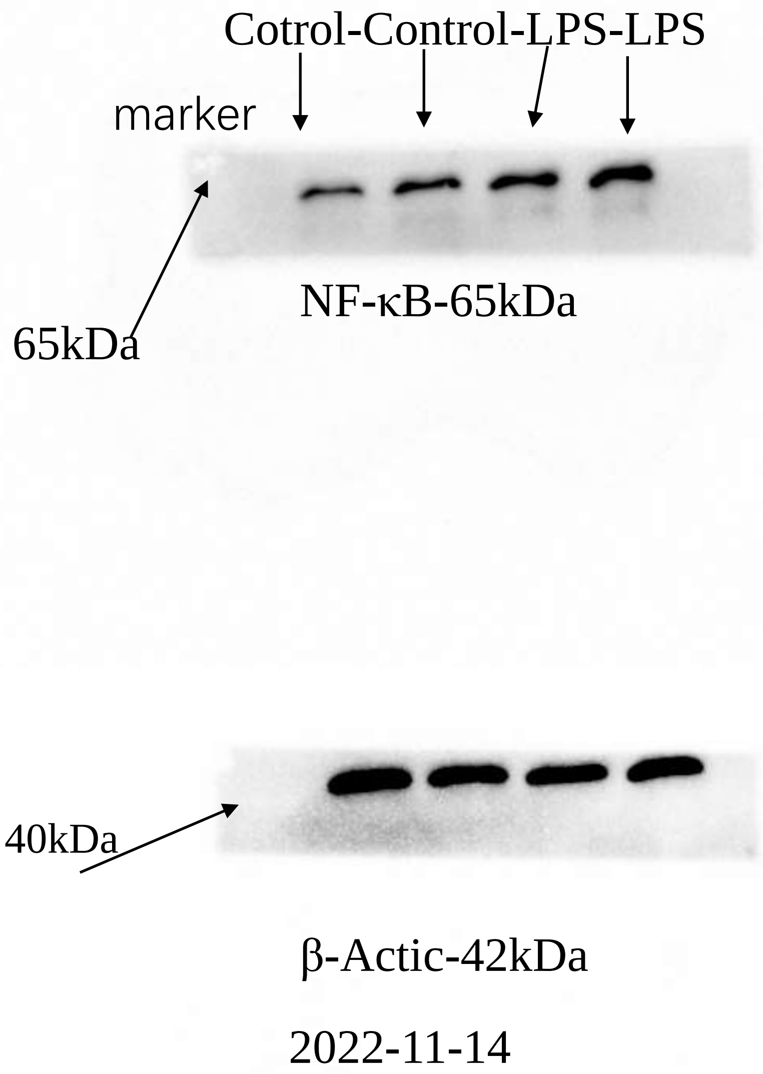
Cotrol-Control-LPS-LPS
marker
65kDa
NF-κB-65kDa
40kDa
β-Actic-42kDa
2022-11-14

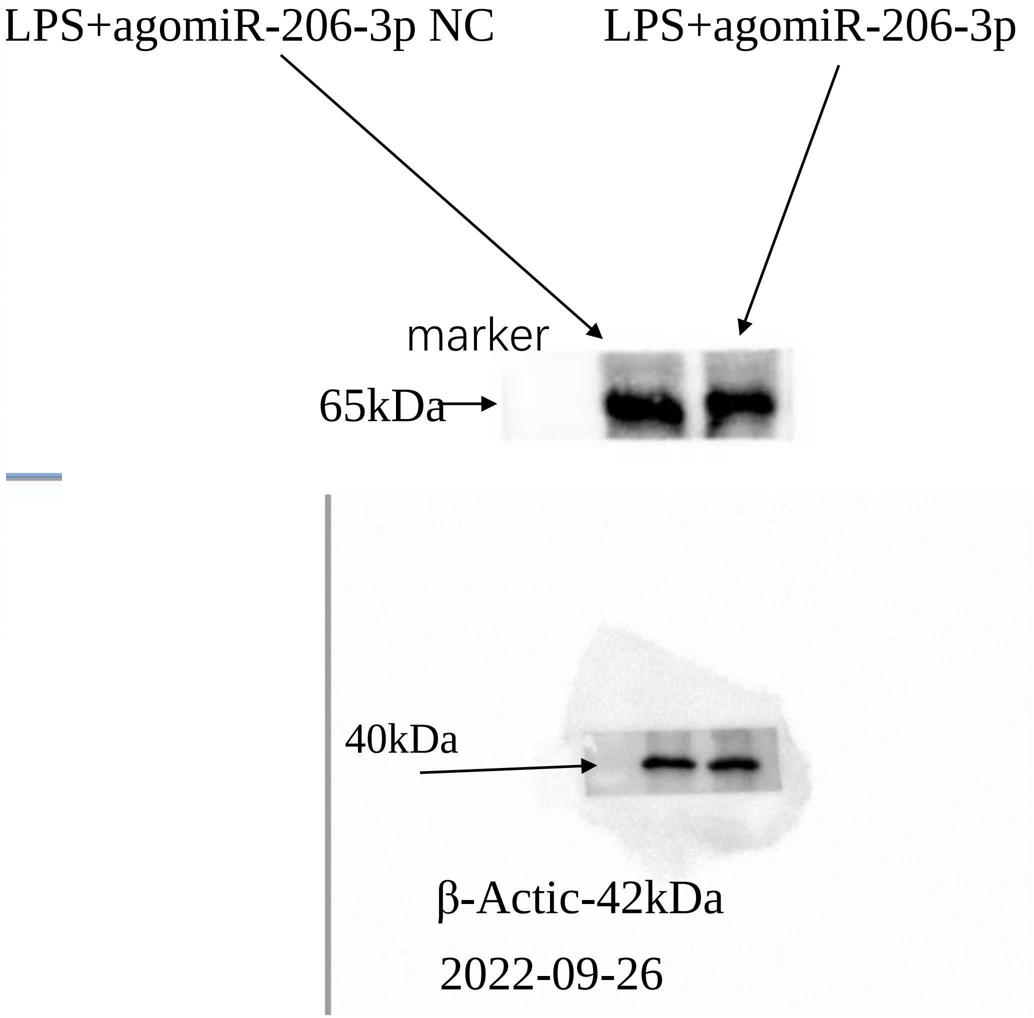
LPS+agomiR-206-3p NC
LPS+agomiR-206-3p
marker
65kDa
40kDa
β-Actic-42kDa
2022-09-26

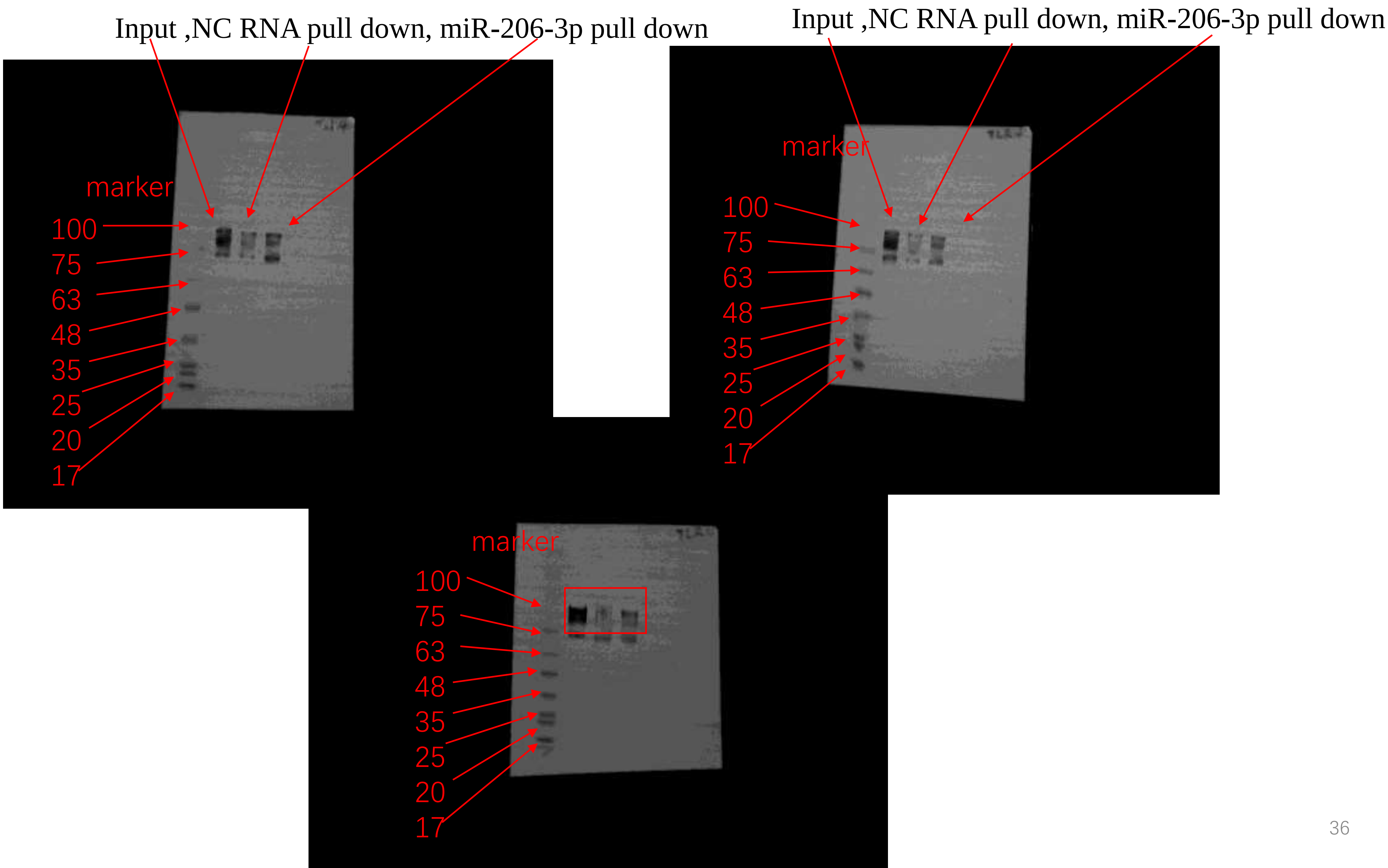
Input ,NC RNA pull down, miR-206-3p pull down
marker
100
75
63
48
35
25
20
17
Input ,NC RNA pull down, miR-206-3p pull down
marker
100
75
63
48
35
25
20
17
marker
100
75
63
48
35
25
20
17

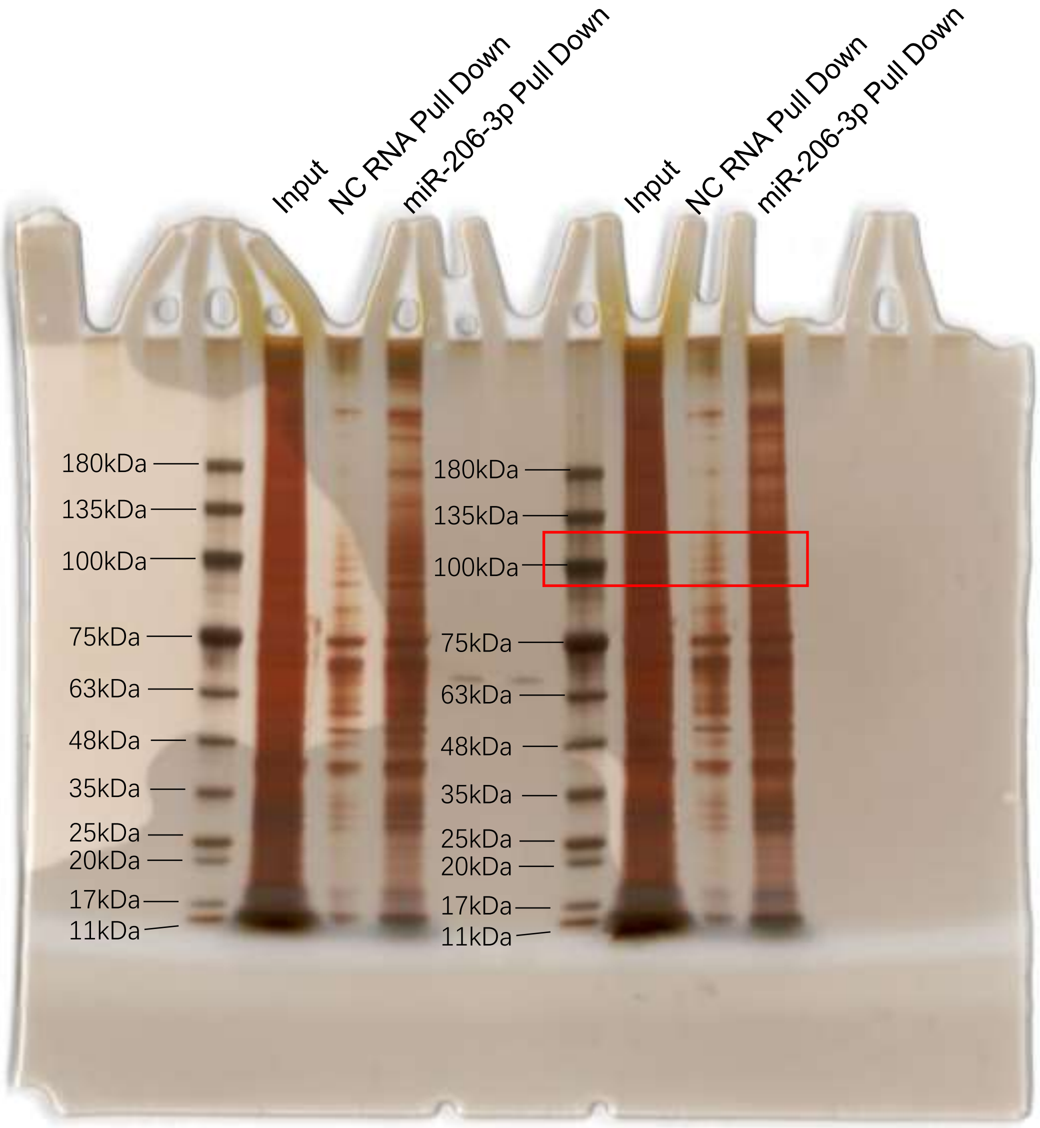
Input
NC RNA Pull Down
miR-206-3p Pull Down
Input
NC RNA Pull Down
miR-206-3p Pull Down
180kDa
135kDa
100kDa
75kDa
63kDa
48kDa
35kDa
25kDa
20kDa
17kDa
11kDa
180kDa
135kDa
100kDa
75kDa
63kDa
48kDa
35kDa
25kDa
20kDa
17kDa
11kDa
